# Comparative quantitative trait loci analysis framework reveals relationships between salt stress responsive phenotypes and pathways

**DOI:** 10.3389/fpls.2024.1264909

**Published:** 2024-02-23

**Authors:** Sunadda Phosuwan, Noppawan Nounjan, Piyada Theerakulpisut, Meechai Siangliw, Varodom Charoensawan

**Affiliations:** ^1^ Doctor of Philosophy Program in Biochemistry (International Program), Faculty of Science, Mahidol University, Bangkok, Thailand; ^2^ Department of Biochemistry, Faculty of Science, Mahidol University, Bangkok, Thailand; ^3^ Biodiversity and Environmental Management Division, International College, Khon Kaen University, Khon Kaen, Thailand; ^4^ Salt-tolerant Rice Research Group, Department of Biology, Faculty of Science, Khon Kaen University, Khon Kaen, Thailand; ^5^ National Center for Genetic Engineering and Biotechnology (BIOTEC), National Science and Technology Development Agency (NSTDA), Khlong Luang, Thailand; ^6^ Integrative Computational BioScience (ICBS) Center, Mahidol University, Nakhon Pathom, Thailand; ^7^ Division of Medical Bioinformatics, Research Department, Faculty of Medicine Siriraj Hospital, Mahidol University, Bangkok, Thailand; ^8^ Department of Biochemistry, Faculty of Medicine Siriraj Hospital, Mahidol University, Bangkok, Thailand; ^9^ Siriraj Genomics, Faculty of Medicine Siriraj Hospital, Mahidol University, Bangkok, Thailand; ^10^ School of Chemistry, Institute of Science, Suranaree University of Technology, Nakhon Ratchasima, Thailand

**Keywords:** comparative omics, quantitative trait loci (QTL) mapping, chromosome segment substitution line (CSSL), salinity stress, systems biology, *Oryza sativa*

## Abstract

Soil salinity is a complex abiotic stress that involves several biological pathways. Hence, focusing on a specific or a few salt-tolerant phenotypes is unlikely to provide comprehensive insights into the intricate and interwinding mechanisms that regulate salt responsiveness. In this study, we develop a heuristic framework for systematically integrating and comprehensively evaluating quantitative trait loci (QTL) analyses from multiple stress-related traits obtained by different studies. Making use of a combined set of 46 salinity-related traits from three independent studies that were based on the same chromosome segment substitution line (CSSL) population of rice (*Oryza sativa*), we demonstrate how our approach can address technical biases and limitations from different QTL studies and calling methods. This allows us to compile a comprehensive list of trait-specific and multi-trait QTLs, as well as salinity-related candidate genes. In doing so, we discover several novel relationships between traits that demonstrate similar trends of phenotype scores across the CSSLs, as well as the similarities between genomic locations that the traits were mapped to. Finally, we experimentally validate our findings by expression analyses and functional validations of several selected candidate genes from multiple pathways in rice and Arabidopsis orthologous genes, including *OsKS7 (ENT-KAURENE SYNTHASE 7)*, *OsNUC1 (NUCLEOLIN 1)* and *OsFRO1 (FERRIC REDUCTASE OXIDASE 1)* to name a few. This work not only introduces a novel approach for conducting comparative analyses of multiple QTLs, but also provides a list of candidate genes and testable hypotheses for salinity-related mechanisms across several biological pathways.

## Introduction

Global climate changes lead to various abiotic stress conditions that can negatively affect multiple aspects of plant growth and development (Patakas, 2012; Sriden and Charoensawan, 2022; Charoensawan et al., 2022). This, in turn, results in fluctuation of major crop production worldwide, causing severe damages to global food security and economy (Ray et al., 2015; Colin et al., 2022). Among the abiotic stresses linked to climate changes, salinity is one of the most concerning problems for the world’s agricultural industry, as over 1,000 million hectares of land is estimated to be salt-affected (Wicke et al., 2011), and this infertile area is predicted to increase by 1.5 million hectares per year (Hossain, 2019). Thus, development of salt-tolerant crops and smart farming technology that alleviate salinity of arable land will contribute towards the solutions of the global food problem.

One of the key strategies implemented to reduce the detrimental effects of salinity and other abiotic stresses on plant growth and development is breeding of climate-proof crops that are resilient to the extreme environments. This process can be greatly assisted and expedited by in-depth understanding of the molecular mechanisms and pathways that plants employ to sense and respond to those environmental changes (Ashraf and Foolad, 2013; Bernardo, 2020). Advances in parallel DNA sequencing technologies have enabled high-throughput screenings of genetic markers of plant abiotic stress responses, as seen in several Quantitative Trait Loci (QTL) and Genome-Wide Association (GWA) studies, e.g (Zhao et al., 2011; Gupta et al., 2019; Tibbs Cortes et al., 2021; Yoshida et al., 2022). However, for abiotic stresses that affect numerous aspects of plant morphology and physiology such as salinity, identification of genomic markers that comprehensively capture several overlapping salt-responsive mechanisms in plants is a challenge (Qin et al., 2020).

Salinity has deleterious influences on several mechanisms of plant development, including impaired photosynthesis (Wang and Nii, 2000; Álvarez and Sánchez-Blanco, 2014), sterility (Abdullah et al., 2001), and losses of biomass and yield (Chartzoulakis and Klapaki, 2000; Maggio et al., 2007) to name a few. Salt stress affects multiple organ systems and tissues, including the root (Julkowska et al., 2017; Arif et al., 2019) as well as leaf and aerial parts (Abbruzzese et al., 2009; He et al., 2022). Numerous measurements and parameters such as the standard evaluation system (SES) of visual salt injury, or salt injury score - SIS herein (IRRI, 2013; Kanjoo et al., 2011; Arif et al., 2019), total chlorophyll content (Do et al., 2019; Tsai et al., 2019), proline content (Ilyas et al., 2020; Pundir et al., 2021), and electrolyte leakage (Julkowska et al., 2016; Zhao et al., 2022) have been used to represent different aspects of these complex traits, leading to genotype-phenotype studies that address specific features of salinity-related traits, as described above. To the best of our knowledge, most studies typically focus on a specific set of related traits of interest, which may not necessarily overlap with one another.

In this study, we aim to establish a framework for integrating physiological and morphological data of rice (*Oryza sativa*) subjected to a variation of salt stresses from multiple QTL studies, and investigate common and unique loci that are linked to different aspects of salinity-related traits. Taking advantage of earlier quantitative salinity-related trait studies performed using the same Chromosome Segment Substitution Line (CSSL) rice populations (Kanjoo et al., 2011; Pamuta et al., 2014; Nounjan et al., 2016), here we highlight genomic loci that appear to be responsible for multiple traits related to salt stress, as well as multiple loci that are linked to overlapping traits. To further refine the list of salt-responsive genes from our comparative QTL analyses, we combined publicly available transcriptomic data of rice grown in a wide range of salt stress conditions, and asked whether or not, and to what extent, the salt-responsive candidates from the QTL studies were differentially expressed in the salinity environments as compared to the non-stressed controls. Finally, we experimentally validated several known and novel salt-responsive candidate genes through analyses of transcription levels in rice and their *Arabidopsis thaliana* orthologues, and examined the phenotypes of the loss-of-function Arabidopsis mutants. Our study provides new insights into salinity-related mechanisms of plants, from the overall relationships between genomic loci and various salinity-related traits, to detailed investigation of salt-responsive candidate genes from diverse biological pathways, which all contribute to the complex salinity responsiveness and tolerance mechanisms in plants.

## Materials and methods

### Chromosome segment substitution line and salinity-related trait datasets

The CSSL populations used as models in this study were first described by Kanjoo et al. (2011). In brief, either of the two “salt-tolerant” lines: DH103 (parental line: IR68586-F2-CA-31) or DH212 (parental line: IR68586-F2-CA-143) were backcrossed into the “salt-susceptible” representative line KDML105 (Khao Dawk Mali 105, *indica* spp.) five times, followed by three selfing generations. This resulted in a total of 135 CSSLs, of which 31 CSSLs have DH103 as the donor parent and 104 CSSLs have DH212 as the donor parent.

The genetic variation data, including single nucleotide polymorphisms (SNPs) of the CSSL populations were described by Shearman et al. (2022). In short, the SNP variant information was generated from the leaf tissues of each CSSL and the parental lines by Genotype-By-Sequencing (GBS) and whole genome sequencing (WGS). Shearman et al. (2022) also identified “informative” 7,714 and 3,171 SNPs in the CSSLs with DH103 and DH212 as the donor parents, respectively. These CSSL populations were distributed and widely used by multiple research groups in Thailand to screen for stress-related lines, and subsequently candidate genes and pathways (Chutimanukul et al., 2013; Kositsup et al., 2013; Homsengchanh et al., 2015; Kulya et al., 2018; Nounjan et al., 2018; Pinta et al., 2018; Chutimanukul et al., 2018a, b; Khrueasan et al., 2019, 2020; Nounjan et al., 2020; Ruangsiri et al., 2021; Shearman et al., 2022). In this study, we gathered salinity-related physiological and phenotypic data from four independent experiments, as illustrated in **Table 1** and **Supplementary Table S1**. From a total of 46 salinity-related traits used this study, we classified them into three major groups: (i) salt injury scores, SISs - the standard evaluation system (SES) of visual salt injury (IRRI, 2013); (ii) tolerant phenotypes (e.g., shoot/root/total dry weight ratio, percentage of survival); (iii) physiological traits (e.g., Na^+^ and K^+^ concentrations, total chlorophyll content, electrolyte leakage, and proline content).

**Table 1:** Summary of physiological and morphological data obtained from the collaborating laboratories (see also **Supplementary Table S1**).

Experiment No.	Traits category**	Short description*	Rice cultivar(s)	Reference
1-15	Salt injury score**	19 DAG* treated with 150 mM NaCl	KDML105, DH103, DH212 and 91 CSSLs	Kanjoo et al. (2011)
16-30	Salt injury score, Tolerant phenotype	16 DAG treated with 100 mM NaCl	KDML105, DH103 and 37 CSSLs	Kanjoo et al. (2011)
31-42	Tolerant phenotype, Physiological trait	21 DAG treated with 175 mM NaCl	KDML105, DH103 and 30 CSSLs	Pamuta et al. (2014)
43-46	Salt injury score	21 DAG treated with 150 mM NaCl	KDML105, DH103 and 30 CSSLs	Nounjan et al. (2016)

*DAG, Days after germination.

**Salt Injury Score (SIS) was determined using the IRRI’s Standard Evaluation System (SES), see Methods.

### Quantitative trait loci identification and comparative QTL analyses

We re-performed QTL analyses for all the CSSLs described above, using three different methods as described below:

(1) Interval mapping: linkage mapping of QTL was carried out using the Haley-Knott regression method with a single-QTL model through the R/qtl package (Broman et al., 2003). The ‘logarithm-of-odds’ (LOD values) of SNP_i_ for each trait were calculated from the interval mapping with 1,000 permutation tests.
(2) Wilcoxon signed-rank test: this single marker analysis (SMA)-based, was performed using the ‘wilcox.test’ function in the ‘stats’ package (R Core Team, 2022). The -log_10_(p) values of SNPs between the salt-susceptible KDML105 and the two salt-tolerant parents for each trait were later used to assess statistical significance of the SNPs (Taheri and Hesamian, 2013).
(3) Logistic regression analysis: the method was based on the equation Y = mX + ε, where Y and X are stress-related value/measurements, and presence/absence (as 1 or 0) of SNP_i_, respectively. ‘m’ represents the SNP effect (coefficient) of each SNP and ϵ corresponds to random errors (Hsu and Tung, 2015). The calculation was performed in R (R Core Team, 2022) using the function ‘glm’, and the option family = ‘binomial’. The -log_10_(p) values from this method were used to represent the significance levels of the salt-responsive candidate SNPs since the other two parameters obtained from the analysis, the coefficient of determination (R^2^) and absolute values of SNP effect (coefficient: |SNP effect|) were correlated with the -log_10_(p) values across all the genomic markers (**Supplementary Figures S1**, **S2**).

We next investigated the results of salinity-related SNPs, chromosomal regions and candidate genes identified by the three methods and obtained the consensus results using the following five steps (see also **Supplementary Figure S3**):

(1) QTL identification analysis of each salinity-related trait as described above.
(2) SNPs between salt-susceptible and salt-tolerant lines that were present in fewer than five CSSLs were considered uninformative and hence excluded from further analyses. The minimal number of CSSLs was determined based on the sensitivity and selectivity when compared against the benchmarking genomic markers and genes characterised by earlier studies on the same populations (Kanjoo et al., 2011; Khrueasan et al., 2013; Nounjan et al., 2016; Chutimanukul et al., 2018b; Khrueasan et al., 2020) (see **Supplementary Table S2**; **Supplementary Figures S4**, **S5**, and more details in Results), and the agreement between the three methods.
(3) As the representative QTL significance levels are on different scales and ranges [namely LOD and -log_10_(p)] (**Supplementary Figure S6**), in order to prioritise and compare the candidate SNPs identified using different methods, we transformed these scores into percentile rank (PR) values, “PR-normalised confidence scores’’ herein. This was done using the ‘quantile’ function in R.
(4) Candidate SNPs with the percentile over 80 from each QTL identification method in at least two methods, based on the resulting sensitivity and selectivity when compared against the benchmarking genomic markers and genes were selected as “high-confidence” SNPs (see **Supplementary Table S3**).
(5) For each chromosome, the salt-responsive regions associated with the SNPs passing the QTL analyses were estimated based on the linkage disequilibrium (LD) and SNP density, resulting in the window size and number of SNPs passing the cut-off within that window (**Supplementary Table S4**).

### Data visualisation of the combined QTL results

Visualisation of high-confidence SNPs detected in two or three QTL identification methods of each salinity-related trait were performed using the ‘Heatmap’ function in the ‘ComplexHeatmap’ R package (Gu et al., 2016). Circos plots were used to demonstrate the relationships between chromosomal regions obtained from the QTL identification pipeline and the salinity-related traits, using the ‘circlize’ R package (Gu et al., 2014). Spearman correlation analyses between multiple salt-responsive traits were computed using the ‘rcorr’ function in the ‘corrplot’ R package (Wei and Simko, 2021), to investigate the correlations between the phenotype scores and the PR-normalised confidence scores. Correlation matrices were used to show overall correlations between all the traits and were colour-displayed using the ‘Heatmap’ function in the ‘ComplexHeatmap’ R package (Gu et al., 2016). Hierarchical clustering for the dendrogram was computed using the Euclidean distance and Ward.D methods, with the ‘dist’ and ‘hclust’ functions, with the method = ‘euclidean’ and ‘ward.D’ in the ‘dendextend’ R package (Galili, 2015).

### Comparative analyses of publicly available transcriptomic datasets

One hundred and two (102) microarray transcriptomes from 20 different salt-treatment experimental settings were obtained from gene expression studies performed using the microarray platform GPL2025. All the experiments were performed on rice (*O. sativa*) at the seedling stage. The transcriptomic data were obtained in the .CEL format, which were downloaded from Gene Expression Omnibus (GEO). For RNA-seq, raw sequences of 400 experiments from 97 different settings of salt-stress treatments were gathered from the NCBI Sequence Read Archive (SRA). All the publicly available microarray and RNA-seq datasets used in this study were summarised in **Supplementary Tables S5** and **S6**, respectively.

For the microarray datasets, each GSE accession was analysed using the ‘ReadAffy’ function with the MAS 5.0 function, followed by conversion to expression values and normalisation using the RMA (Robust Multi-array Analysis) method, within the ‘affy’ R package (Bolstad et al., 2003). Genes corresponding to a probe set were characterised by the Affymetrix annotation combined with TIGR definition and the International Rice Genome Sequencing Project (IRGSP build 1.0) (Cao et al., 2012). Differentially expressed gene (DEG) analyses were performed separately for different experimental settings described in **Supplementary Tables S5** and **S6**, using the cut-offs of p-value < 0.05 and |log2 fold-change| ≥ 0.2. The fold-change cut-off was determined from the average fold-change of housekeeping genes (see **Supplementary Figure S7**), where the housekeeping gene list was taken from Narsai et al. (2010). DEGs were included into the following steps if they pass the cut-offs in at least three independent experimental settings.

For the RNA-seq datasets were analysed using a pipeline described by Sriden and Charoensawan (Sriden and Charoensawan, 2022) with minor modifications. In brief, the SRA files obtained from GEO were converted to FASTQ using the ‘fastq-dump’ function in the SRA toolkit from NCBI (https://github.com/ncbi/sratoolkit). The raw reads were checked for the quality using FastQC v0.11.5 (Andrews et al., 2015). Illumina adapters (TruSeq3) and low-quality reads were trimmed using Trimmomatics 0.36 (Bolger et al., 2014). The trimmed reads were then mapped to the *Oryza sativa* cv. Nipponbare IRGSP build 1.0 reference genome (Kawahara et al., 2013) using HISAT2 (Kim et al., 2015). The optical duplicates were marked and removed using Picard v1.139 (http://broadinstitute.github.io/picard/). The remaining reads were sorted by names using Samtools (Li et al., 2009). The transcripts were then quantified for each gene by HTSeq 0.6.1p1 (Anders et al., 2015). DEGs were identified by the ‘DESeq2’ R package (Love et al., 2014) using p-value < 0.05 and |log2 fold-change| ≥ 2, which is the average fold-change of housekeeping genes (see **Supplementary Figure S7**). DEGs were included into the following steps if they pass the cut-offs in at least three independent experimental settings.

### Salinity-related expression in rice and Arabidopsis

All validation experiments carried out in rice were conducted at the Rice Science Center, Kasetsart University, Kamphaeng Saen, Nakhon Pathom, Thailand. The growth protocol and details were as described in Songtoasesakul et al. (2023). In brief, seeds of KDML105, DH103 and DH212 rice cultivars were germinated in plastic trays and placed in the cement tank flooded with the Bangsai nutrient solution (1:100), which contains 50 g/L MgSO_4_, 80 g/L KNO_3_, 12.5 g/L NH_4_H_2_PO_4_, 8.5 g/L KH_2_PO_4_, 0.4g/L Mn-ethylenediaminetetraacetic acid (EDTA), 0.8g/L micronutrients, 100 g/L Ca(NO_3_)_2_ and 3 g/L Fe-EDTA. Rice seedlings at 16-day-old were then grown in either normal nutrient solution or the one supplemented with 100 mM NaCl.

Arabidopsis experiments were conducted at the Faculty of Science, Mahidol University, Bangkok, Thailand. Seeds of Columbia-0 (wild type, Col-0) were sterilised and stratified in 0.1% agar and incubated at 4°C in the dark for 3 days. Stratified seeds were transferred to pots containing a growing mixture containing peat moss, perlite and vermiculite at the ratio of 3:1:1. Seedlings were grown under a long-day photoperiod (light:dark of 16:8) at 22 ± 1°C in a temperature-controlled cabinet. Ten-day-old plants were treated with 100 mM NaCl for salt-stress experiments. A randomised complete block design (RCBD) with two biological sets of replications were performed. The treatment and control groups, treated for 1, 3 and 5 days in rice, and 3 and 7 days in Arabidopsis, were sampled and immediately frozen in liquid nitrogen and stored at -80 °C.

RNA extraction of the aerial part of rice, and whole seedlings of Arabidopsis were carried out using TRIzol reagent (Invitrogen, CA, USA), using the protocol described in (Azizi et al., 2017) for rice, and following manufacturer’s instruction for Arabidopsis. Genomic DNA removal and cDNA synthesis were performed using ReverTra Ace^®^ qPCR RT Master Mix with gDNA remover (TOYOBO CO. LTD, Japan). Transcription level was determined by quantitative PCR using the THUNDERBIRD SYBR qPCR mix (TOYOBO CO. LTD, Japan) and the QuantStudio 12K Flex real-time PCR system (Life Technologies, CA, USA) for rice, and the CFX96 real-time PCR system (Bio-Rad, CA, USA) for Arabidopsis. The cycling conditions were performed as follows: 5 min at 95°C followed by 40 rounds of 15 s at 95°C, 30 s at 50-55°C, 30 s at 72°C, and 1 round of 30 s at 55°C. The oligonucleotide primers used in this study are listed in **Supplementary Table S7**. At least four (for rice) and six (for Arabidopsis) biological replicates with three technical replicates were used for RT-qPCR analyses. *OsEF1A* (*ELONGATION FACTOR 1A*, Os03g0177750) and *AtPEX4* (*PEROXIN 4*, AT5G25760) were used as reference genes for rice and Arabidopsis, respectively. Relative transcription levels were calculated using the 2^-ΔΔCt^ method (Livak and Schmittgen, 2001) and statistically evaluated using ANalysis Of VAriance (ANOVA) followed by Tukey *posthoc* comparison. These tests were done using the ‘aov’ and ‘TukeyHSD’ functions in the 'stats' R package and the plots were done using the 'ggpubr' package (Kassambara, 2023).

### Experimental functional analysis in Arabidopsis orthologues

Orthologous rice-to-arabidopsis gene list was obtained using BioMart (https://plants.ensembl.org/biomart/) available at EnsemblPlants (Kinsella et al., 2011). Gene IDs of *Oryza sativa* Japonica Group genes (IRGSP-1.0) were used as references to fetch a list of orthologous Arabidopsis genes from the *Arabidopsis thaliana* (TAIR10) dataset. Only orthologous genes with 1-to-1 relationships were used in this study.

Functional validations in Arabidopsis mutants were conducted at the Faculty of Science, Mahidol University, Bangkok, Thailand, using the same set-up as described above. Arabidopsis mutant lines were obtained from the Arabidopsis Biological Resource Center (ABRC) (see **Supplementary Table S13**). Seven-day-old (7) wild-type (Col-0) and mutants were then treated with 250 mM NaCl for salt-stress experiments, as in Chutimanukul et al. (2018b) and Khrueasan et al. (2019). The treatment and control groups (5 seedlings, at least 4 biological replications), treated for 12 days, were photographed with a 12-megapixel camera of an iPhone 12 Pro (Apple Inc, USA). The leaf area and rosette diameter were visualised from the top-view and measured using ImageJ 1.53 (Schneider et al., 2012). The seedlings were then harvested, washed, and measured fresh and dry weights using a weighing machine (Sartorius BSA224S, Max 220 g, ± 0.001mg sensitivity, Germany). P-values of the differences between the plants grown under the control and salt-stress conditions were computed using ANOVA, followed by the Tukey *posthoc* comparison, when applicable.

## Results

### Overview of integrative and comparative QTL analyses

**Figure 1:**
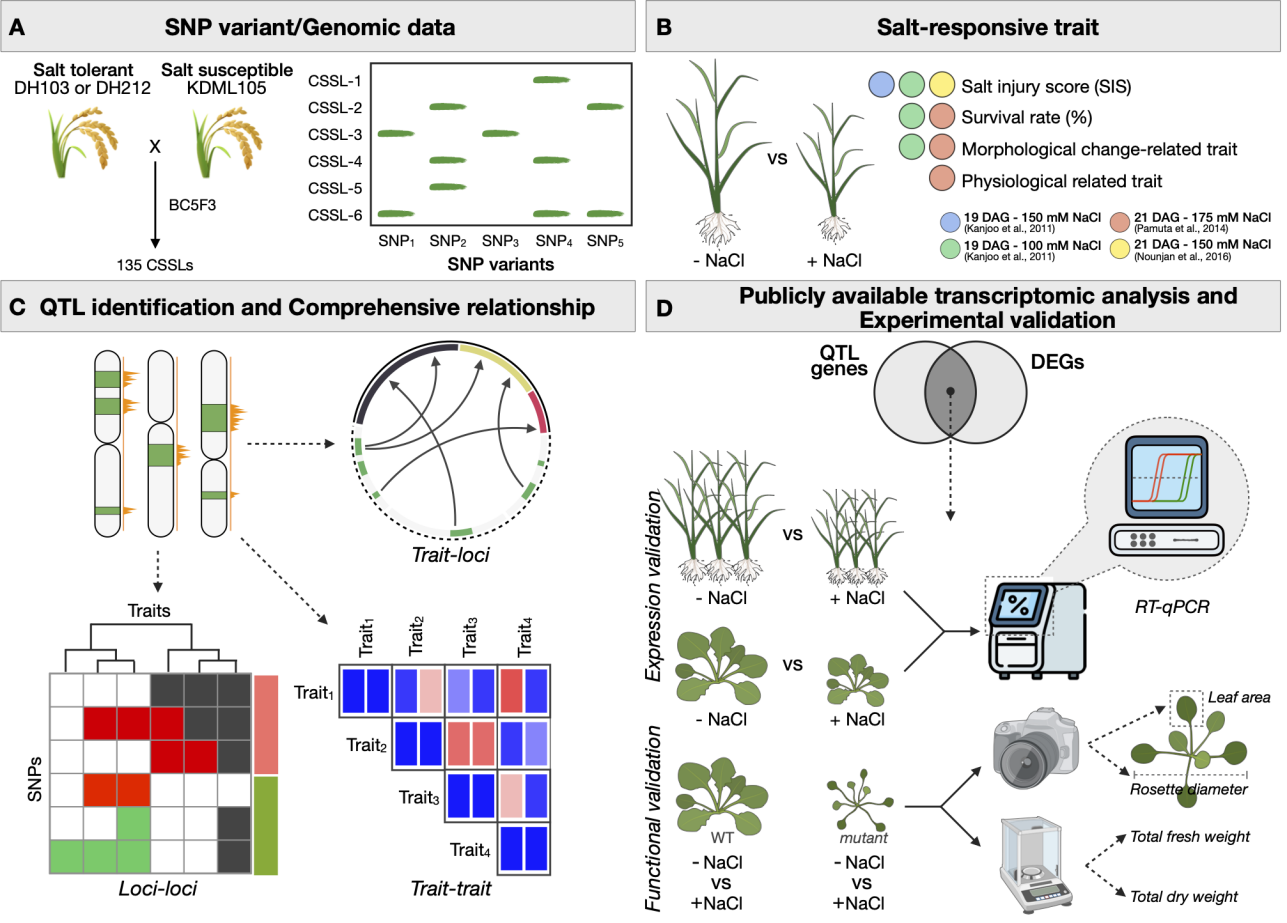
Overview of our comparative quantitative trait loci (QTL) analysis framework for investigating the relationships between multiple salt-responsive phenotypes. **(A)** A summary of crossing strategy and genomic information of the CSSL populations used as a model in this study. The phenotype and trait information were obtained from Kanjoo et al. (2011), Pamuta et al. (2014) and Nounjan et al. (2016), and the genomic variations were from Sherman et al. (2022). **(B)** A summary of salinity-related traits obtained from three studies and four experimental settings described above. **(C)** A summary of QTL identification steps and comprehensive investigations of trait-loci and trait-trait, and loci-loci involved in salt responsiveness. **(D)** A summary of expression level and functional validations in rice and Arabidopsis.

**Figure 1** summarises the overview of integrative and comparative analyses of QTL and transcriptomic data in this study. Using one consistent pipeline, we first obtained and re-analysed the data of salinity-related phenotypic and physiological traits from 46 independent experiments and conditions performed on the same set of CSSL rice populations, as previously described (Kanjoo et al., 2011; Pamuta et al., 2014; Nounjan et al., 2016) (see also Methods). In brief, two salt-tolerant cultivars, DH103 and DH212, were separately crossed into the salt-susceptible cultivar KDML105. The distributions of 7,714 and 3,171 informative SNPs between KDML105 and the two salt-tolerant parents, as identified by Shearman et al. (2022), are shown in **Supplementary Figures S8** and **S9**. We observed haplotypic regions with 100 informative SNPs or higher within 1 Mb genomic regions in Chromosomes 1, 2, 3, 4, 8 and 9 of the DH212-derived CSSLs; and in Chromosomes 1, 2, 3, 4, 7 and 8 of the DH103-derived CSSLs. These were considered the introgressed chromosomal segments of the salt-tolerant cultivars in the genome of KDML105. **Supplementary Figures S10** and **S11** show the distributions of informative SNPs in each of 104 DH212-derived and 31 DH103-derived CSSLs, spreading across different chromosomes in the two populations. For the salinity-related traits investigated here, we saw variations of salt-responsive characteristics (e.g., SIS, dry weight, total chlorophyll content, electrolyte leakage) among the CSSLs, while the susceptible KDML105 parent showed apparent susceptible characteristics (e.g., higher SIS, lower total chlorophyll content, higher electrolyte leakage), as compared to those of the two tolerant parents (**Supplementary Figure S12**). These together showcase the suitability of the genomic and phenotypic data to be used in further comparative QTL analyses.

### A framework for integration of SNPs from multiple salinity-related traits

To unbiasedly investigate the genetic marker-trait association and identify the salinity-related QTLs of each of the 46 traits obtained in different studies, we applied three different QTL identification methods to investigate the associations between SNPs and the phenotypic data described above, namely interval mapping, Wilcoxon signed-rank test, and logistic regression (see Methods). As the confidence scores from different QTL identification systems are on different scales and ranges (**Supplementary Figure S6**), we transformed these scores into percentile rank (PR) values, so that they can be compared in terms of ranking rather than parametric scales. In other words, these “PR-normalised confidence scores” serve as a method to prioritise SNPs according to their likelihood to be associated with the salinity-related traits of interest. This approach did not compromise or distort the variation patterns observed between salinity-related and non-salinity-related SNPs (**Supplementary Figure S13**).

We next established a framework to compare the salinity-related SNPs identified by different methods. Firstly, we assessed the consistency between the PR-normalised confidence scores from different methods, and the ability to recover known salinity-related QTLs and genes from earlier studies on these CSSL populations (**Supplementary Table S2**, see Methods). Looking into the numbers of CSSLs with the SNP alleles from the tolerant cultivars (out of 104 DH212-derived lines and 31 DH103-derived lines in total) (**Supplementary Figure S4**), we observed that the correlations between methods improved as we excluded the SNPs that were present in fewer than five CSSLs (**Supplementary Figure S4**). We also assessed the ability of the framework to distinguish the known salinity-related SNPs (as described above, see **Table S2**), from the rest of the SNPs. We observed the intercept between the selectivity and sensitivity of recovering these known salinity-related SNPs when the SNPs are present in at least five CSSLs (**Supplementary Figure S5**). After this filtering process, there were 745 and 2,431 SNPs passing the cut-offs from the DH212- and DH103-derived populations, respectively.

Next, we investigated the minimum PR-normalised confidence scores that gave the best power to recover the known salinity-related SNPs based on the previous studies, by plotting the changes in sensitivity and selectivity (**Supplementary Figure S14**), as well as the percentages of salinity-related genes and all genes being retained using different PR confidence score cut-offs (**Supplementary Figure S15**). Both plots suggested that the most optimal cut-off that distinguished known salinity-related SNPs and genes from the rest is percentile 80. After this PR score filtering step, there are on average of 266 SNPs ( ± 33, SD) from the DH212-derived population, and 974 SNPs ( ± 212, SD) from the DH103-derived population, left for further investigation (**Supplementary Table S8**). Note that the numbers of SNPs passing the cut-offs in at least one QTL identification method varied between different traits.

### Comprehensive trait-loci relationships detected by different QTL methods

The results of the salt-responsive SNPs detected by three QTL identification methods described earlier were summarised in **Supplementary Table S9**. As expected, the two methods showing more overlapping results are logistic regression and Wilcoxon test, as they are both single marker analyses (SMAs) approaches. In contrast, the interval mapping method is a linkage analysis (Haley and Knott, 1992), which takes into account the confidence scores of adjacent SNPs (**Supplementary Figure S16** and **Supplementary Table S9**). **Supplementary Figure S17** demonstrates the locations of these SNPs from the QTL analysis and their flanking genomic regions (see Methods for more details) For the following in-depth comparative analyses, we narrowed down the genomic regions of interested by including only the SNPs that passed the cut-offs in at least two QTL identification methods, which means the regions of interest were down to approximately one-fifth of the initial genomic positions covered by the informative SNPs (**Supplementary Figures S3**, **S8**, **S9**, **S17**).

**Figure 2:**
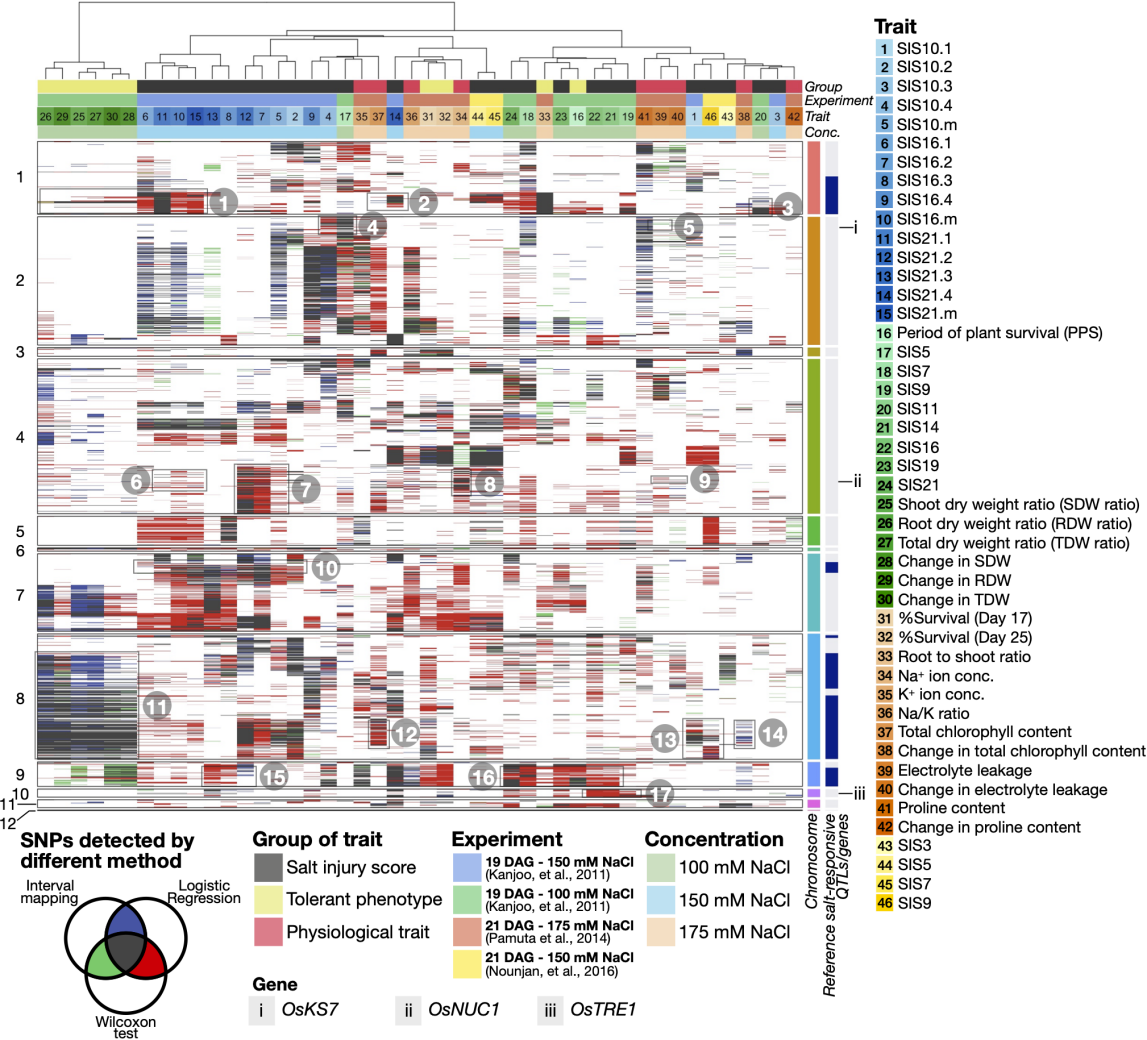
Heatmap summarising high-confidence SNPs and salinity-related gene candidates identified in 46 salt-responsive traits. High-confidence SNPs were characterised if they are present in at least five CSSLs and passed the 80th percentile confidence scores in two or three QTL identification methods, as indicated in the heatmap in ‘blue-green-red’ or ‘black’, respectively (see Methods for more details). Columns and rows represent salinity-related traits and SNPs across the chromosome numbers indicated on the left of the heatmap. Colour codes of the traits, experimental settings salt concentrations, and examples of salinity-related QTL/genes are also described (see also high-resolution version in **Supplementary Materials**).

Among the salinity-related SNPs and genes identified by this approach, we observed several high-confidence loci and SNPs found by all the three QTL identification methods (black boxes in **Figure 2**), as seen in earlier studies (as summarised in **Supplementary Table S2**). For instance, we observed the SNPs linked to SIS on Chromosome 1 (see Boxes 1, 2 and 3 indicating the positions of SNPs associated with the trait numbers 6, 11, 15, 14, and 20 in **Figure 2**, for instance), as previously characterised by Khrueasan et al. (2013), Chutimanukul et al. (2018b) and Khrueasan et al. (2020). High-resolution **Figure 2** and additional candidate genes can be found in Extended Results, available as **Supplementary Materials**. For the SNPs that were identified by at least two QTL identification methods (red, green, and blue boxes in **Figure 2**), we found additional SNPs linked to SIS, such as on Chromosome 7 (Box 10 - trait no. 6-8 and 10) and on Chromosome 9 (Boxes 15 and 16 - trait no. 8 and 22), as characterised by Kanjoo et al. (2011), as well as SNPs on Chromosome 8 that are related to dry weights (Box 11 - trait no. 25, 27 and 29), SIS (Box 13 and trait no. 1 and 46) and photosynthesis adaptation (Boxes 12 and 14 - trait no. 37 and 38), as characterised by Nounjan et al. (2016). These exemplify the ability of our integrative framework in terms of re-discovering characterised salinity-related QTLs and genes from earlier studies.

We also observed multiple SNPs detected by at least two QTL mapping methods that have been demonstrated to be linked to salinity in other studies conducted in different plants to our CSSL populations. For instance, we found SNPs associated with SIS (Boxes 6 and 7 - trait no. 5, 7 and 10) and ion balance adaptation (Boxes 8 and 9 - trait no. 34, 39 and 40) on Chromosome 4 (see **Figure 2**), being linked to a salt-responsive gene *OsNUC1* (*NUCLEOLIN 1*, Os04g0620700) (see Gene ii), as previously identified by Sripinyowanich et al. (2013). Similarly, more SNPs associated with SIS (Box 17 - trait no. 19, 21 and 22) can be linked to the cellular homeostasis and salt-responsive gene *OsTRE1* (*TREHALASE 1*, Os10g0521000) (see Gene iii), as described by Islam et al. (2019).

Importantly, we have found high-confidence QTL regions with novel candidate salinity-related genes, of which to the best of our knowledge, their salt-responsive functions have not been characterised before. These include the gene phytoalexin biosynthesis-related *OsKS7* (*ENT-KAURENE SYNTHASE 7*, Os02g0570400) (see Gene i on Chromosome 2), which is associated with SIS10 (Box 4 - trait no. 4) and electrolyte leakage (Box 5 - trait no. 39). These together showcase that our comparative QTL approach can re-discover salinity-related SNPs and genes previously characterised in the same and independent populations, as well as suggest additional candidates that will be further explored in the next sections.

### Investigating trait-specific and multi-trait loci associated with salt-stress phenotypes

**Figure 3:**
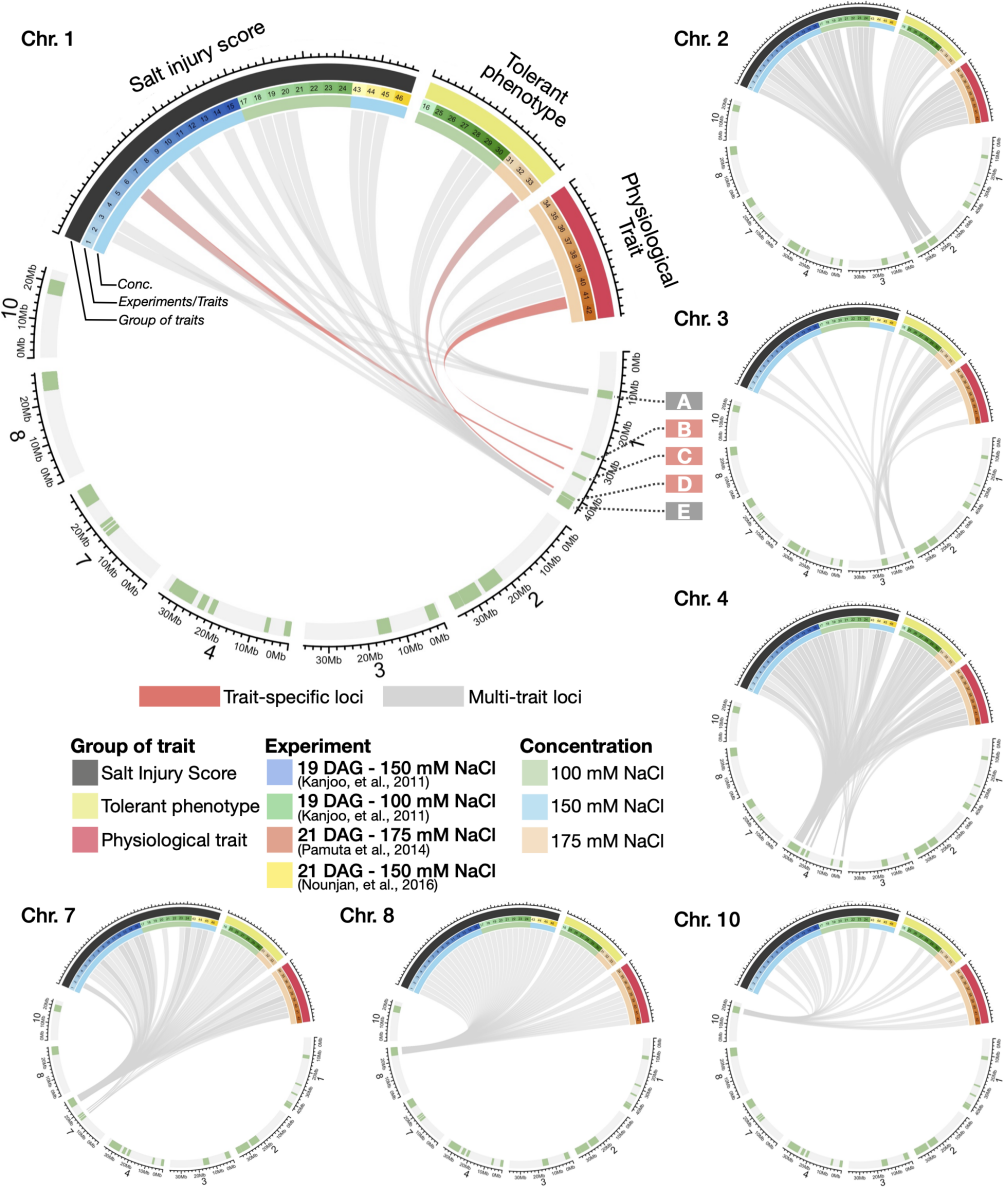
Circos plots summarising the relationships between salt-responsive traits and high-confidence QTLs. High-confidence QTLs were identified on Chromosomes 1, 2, 3, 4, 7, 8 and 10, using the same criteria described in **Figure 2**. The colours of the line linking traits to genomic locations represent the trait-specific (pink) and multi-trait (grey) loci on the QTL regions.

Having identified salinity-associated QTLs for each of the 46 salinity-related traits, we next asked if and to what extent multiple salinity-related traits were mapped to overlapping loci (which shall be referred to as “multi-trait loci” herein), and in contrast, those loci that are uniquely associated with specific traits (“trait-specific loci” herein). We classified salinity-related traits into three major groups: (i) salt injury scores (SISs) of plants after different salt-stress treatment periods (indicated by black bars in Circos plots, **Figure 3**); (ii) tolerant phenotypes (e.g., shoot/root/total dry weight, percentage of plant survival, as indicated by yellow bars); (iii) physiological traits (e.g., Na^+^ and K^+^ concentrations, total chlorophyll content, electrolyte leakage, and proline content, indicated by red bars) (see also Methods and **Supplementary Table S10**).

Looking through different chromosomes, most of the high-confidence QTLs in this study were linked to more than one groups of salinity-related traits (indicated by grey lines in Circos plots), (see also a complete list of QTL-trait relationships in **Supplementary Table S10**). For instance, Chromosomes 1 Region A was associated with several types of traits including SIS (trait no. 2, 9 and 17), K^+^ concentration (trait no. 35), electrolyte leakage (trait no. 39 and 40) and proline content (trait no. 41). Within this region, we found examples of known salt-responsive genes such as *OsIF* (*INTERMEDIATE FILAMENT*, Os01g0292700), which is related to seedling growth, electrolyte leakage and proline content adaptation under salinity as shown in a previous study (Soda et al., 2016). We also observed another multi-trait locus on Chromosome 1 Region E (**Figure 3**), being linked to SIS of different plant stages (trait no. 3, 6, 9, 11, 14, 18-20 and 43-45), dry weights (trait no. 28-30), root to shoot ratio (trait no. 33), and change in total chlorophyll (trait no. 38). Other multi-trait loci and genes of interest were also observed in Chromosomes 2 (e.g., *OsKS7*, Os02g0706900), 3 (*OsEXPA21*, Os03g0377100), 4 (*OsFRO1*, Os04g0578600 and *OsNUC1*, Os04g0620700) and 8 (*OsSRWD1*, Os08g0497600) (see **Supplementary Table S10**), for instance. Additional salinity-related candidate genes are described in Extended Results and **Supplementary Tables S11** and **S13**.

In contrast to the multi-trait loci, our comparative analysis also revealed several genomic regions and candidate genes specifically associated with certain stress phenotypes in this study, or “trait-specific gene”. To illustrate this point, Chromosome 1 Region B was associated with proline content (trait no. 41). As expected, this genomic region contains proline production-related genes under salt stress in plants such as *OsGLT1* (*NADH-DEPENDENT GLUTAMATE SYNTHASE 1*, Os01g0681900), and glutathione transferase genes *OsGSTU39* (*TAU GLUTATHIONE S-TRANSFERASE 39*, Os01g0692100) and *OsGSTU40* (*TAU GLUTATHIONE S-TRANSFERASE 40*, Os01g0692000). In Region C of Chromosome 1, which was specifically linked to SIS16 (trait no. 6), we found *OsSUVH7 (SUVH HISTONE METHYLTRANSFERASE 7*, Os01g0811300), which has been shown to be associated with seedling growth adaptation under salinity (Wang et al., 2020). On Chromosome 1 Region D, which was linked to trait no. 33 - root to shoot ratio, we found the oxidative stress related gene *OsSPL2 (RICE SQUAMOSA PROMOTER-BINDING-LIKE 2*, Os01g0922600), and cytokinin receptor *OsHK3* (*HISTIDINE KINASE 3*, Os01g0923700), which have been shown to be associated with the development of the root and aerial tissues in earlier studies (Ito and Kurata, 2006; Yue et al., 2017). The full list of multi-trait and trait-specific loci can be found in **Supplementary Table S10** and the candidate genes within the loci are listed in **Supplementary Table S11**.

### Relationships between different salinity-related traits and phenotypes

Salt-responsive mechanisms in plants are complex and involve multiple biological processes and pathways. Having investigated the links between the QTL regions and multiple salinity-related traits in this comparative study, here we looked more closely into the relationships between these different traits, morphological and physiological phenotypes, by investigating their correlations across the CSSLs, using the tolerant and phenotype scores (as obtained directly from the three studies: Kanjoo et al. (2011), Nounjan et al. (2016) and Pamuta et al. (2014), see **Supplementary Figure S18**). Alternatively, here we also analysed the correlations between traits using their PR-normalised confidence scores from our QTL comparative analysis pipeline, which reflect the likelihoods of two or more traits being linked to similar overlapping genomic regions (see **Supplementary Figure S19** and Methods for details).

**Figure 4:**
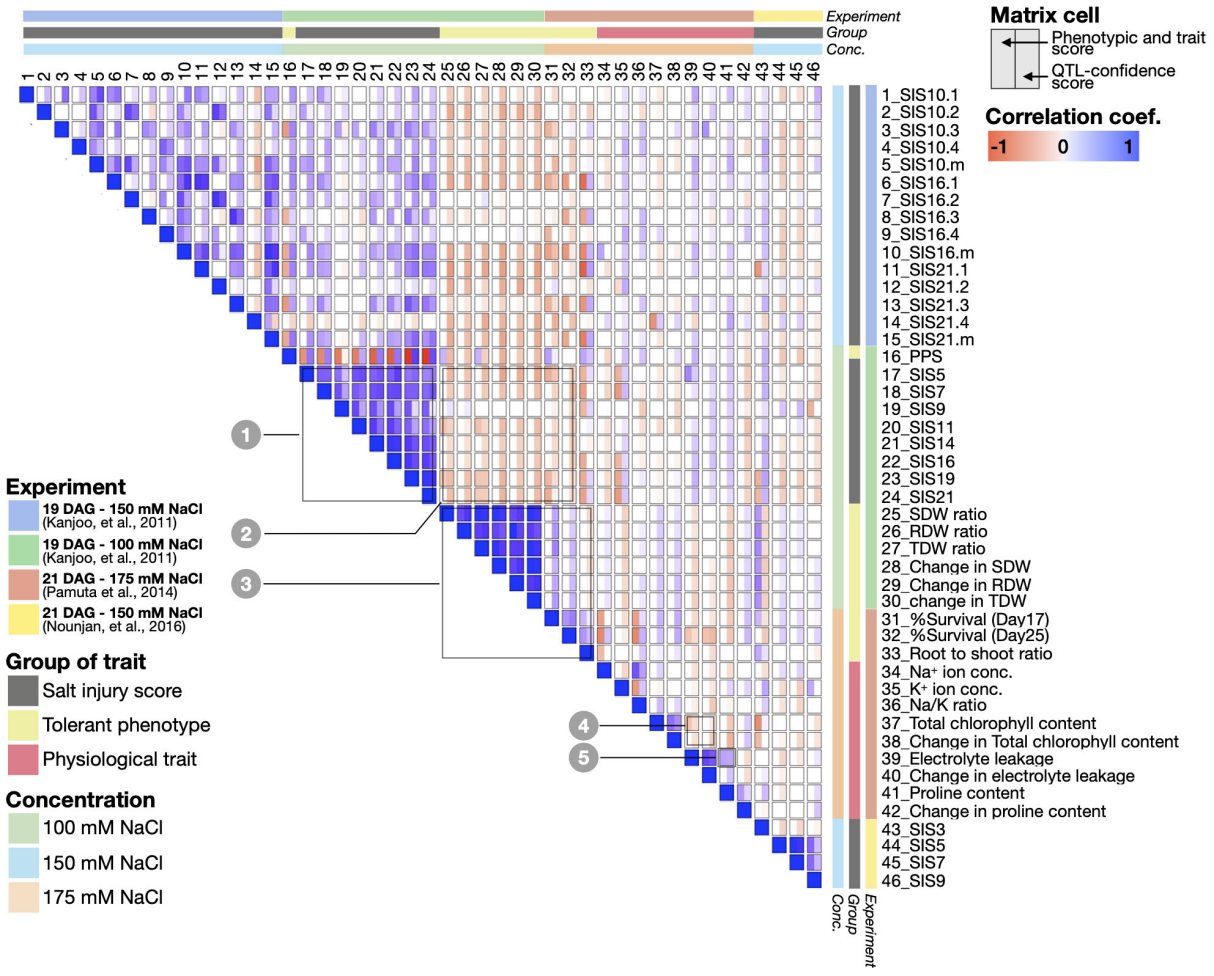
Spearman correlation matrix representing the relationships between 46 salt-responsive traits combined and analysed in this study. The correlations were computed using two different sets of numbers: the phenotype and trait scores directly taken from Kanjoo et al. (2011), Pamuta et al. (2014) and Nounjan et al. (2016), (shown on the left half of each cell), and the and QTL-confidence scores from this study (shown on the right half of each cell). The blue and red represent the correlation coefficient values (p<0.05).

As expected, **Figure 4** and **Supplementary Figure S19** demonstrate clear positive correlations between closely related traits, such as the SISs of plants obtained at 5-21 days after the salt treatment (see Box 1 - trait no. 17 - 24); or among similar tolerant phenotypes (e.g., shoot/root/total dry weight ratio, percentage of survival) (see Box 3 - trait no. 25 - 33). See **Supplementary Table S12** for a complete list of trait-trait correlations. Apart from the trait scores themselves, the relationships between traits can also be assessed through the correlations of confidence scores from the QTL analyses, where positive correlations between two traits means they tend to have trait-associated SNPs and genes at the same or overlapping genomic locations. **Figure 4**; **Supplementary Table S12** summarise the trait correlations based on the phenotype scores (**Supplementary Figure S18**) as compared to those from the QTL confidence scores (**Supplementary Figure S19**).

Indeed, we observed overall agreements between the two methods. For instance, the traits that exhibit salt sensitivity such as SIS and electrolyte leakage show similar trends of phenotype score across the CSSLs, and these traits were also mapped to highly overlapping genomic locations (**Figure 4**, positive correlations based on the phenotype and QTL confidence scores, see Boxes 1 and 5). On the contrary, we also observed the traits with opposite trends of the phenotype scores and also mapped to different genomic regions, including SIS vs’ dry weight; SIS vs’ survival rate, and chlorophyll content vs’ electrolyte leakage, for instance (**Figure 4**, negative correlations based on the phenotype and QTL confidence scores, see Boxes 2 and 4, **Figure 4**).

Interestingly, there are some traits that do not show agreement between the phenotype and QTL scores. For instance, the phenotype scores of SISs tend to be negatively correlated with the root to shoot ratio (trait no. 33) and PPS (trait no. 16), but these traits were mapped to the same genomic locations, and hence have positive correlations based on the QTL scores. Conversely, PPS and dry weights (trait no. 25 and 27) was positively correlated based on phenotype scores, as we saw high PPS and dry weight in salt-tolerant CSSLs. However, the two traits were mapped to different genomic locations and suggest the traits are controlled by a different set of genes (**Figure 4**). With our comprehensive documentation (**Figure 4**, **Supplementary Table S12**), we have established detailed connections between salt-responsive phenotypes, and the genomic regions and genes linked to these phenotypes of interest. This, in turn, provides testable hypotheses to explore novel salt-responsive mechanisms and their interplay in greater details.

### Refining salinity-related QTL candidates using salt-stress transcriptomes

We have so far demonstrated the applications of our integrative framework for analyses of QTL using different traits from multiple studies, which could narrow down salt-responsive genomic locations. However, we noted that one of the main limitations of this particular dataset is the resolution of SNP markers of the CSSL populations, which were statistically adequate, but still returned large QTL genomic regions containing several candidate genes. Here, we asked whether publicly available large-scale omic data, such those from transcriptomic studies performed under salt-stress conditions (e.g., from expression microarray and RNA-seq studies, as summarised in **Supplementary Tables S5** and **S6**, respectively), could be used to further pinpoint the salt-responsive genes.

Based on a simplified assumption that the genes whose functions are related to stress-related mechanisms should be more differentially expressed between the stress and normal conditions, as compared to housekeeping genes, indeed we found that the known housekeeping genes (based on earlier studies by Narsai et al. (2010) and Molla et al. (2015) were expressed with notably lower variations under salt-stress as compared to non-salt-stress conditions than other genes in the genome (**Supplementary Figure S7**). If we arbitrarily chose the transcriptional variations of the housekeeping genes as the cut-offs for salt-responsive genes (see Methods), we could further narrow down the salinity-related candidate genes from the integrative QTL framework of 5,077 to 1,093 genes (**Supplementary Figure S20**). We noted; however, that even though this approach is useful for extracting high-confidence salinity-related candidates, it entails a simplified assumption that did not take into account the genomic variations within the protein-coding genes that might directly affect the protein functions, rather than the expression levels of the genes.

### Experimental validations of salinity-related candidates in rice and *Arabidopsis thaliana*

In addition to the framework for integrating multiple QTL datasets and traits, and comprehensively investigation of overall relationships between trait-loci, trait-trait, loci-loci, and potential links to transcriptomic changes, this work provides a comprehensive list of candidates and testable hypotheses of salt-responsive mechanisms from different biological pathways, including ion transport and homeostasis, membrane integrity, photosynthesis, signal transduction, Ca^2+^ sensing, redox controls, chromatin modification and several transcription factors. To this end, we performed experimental validations on a number of chosen known and novel salinity-related genes using gene expression analysis of the candidates in *Oryza sativa*, (DH103, DH212 and KDML105 cultivars) and in *Arabidopsis thaliana* (Col-0), together with functional analyses using lack-of-function mutants in *Arabidopsis thaliana*. See **Supplementary Table S11** for a complete list of salinity-related candidates, a list of candidate genes being validated in **Supplementary Table S13**, and Extended Results for additional descriptions and validations of the candidate genes.

**Figure 5:**
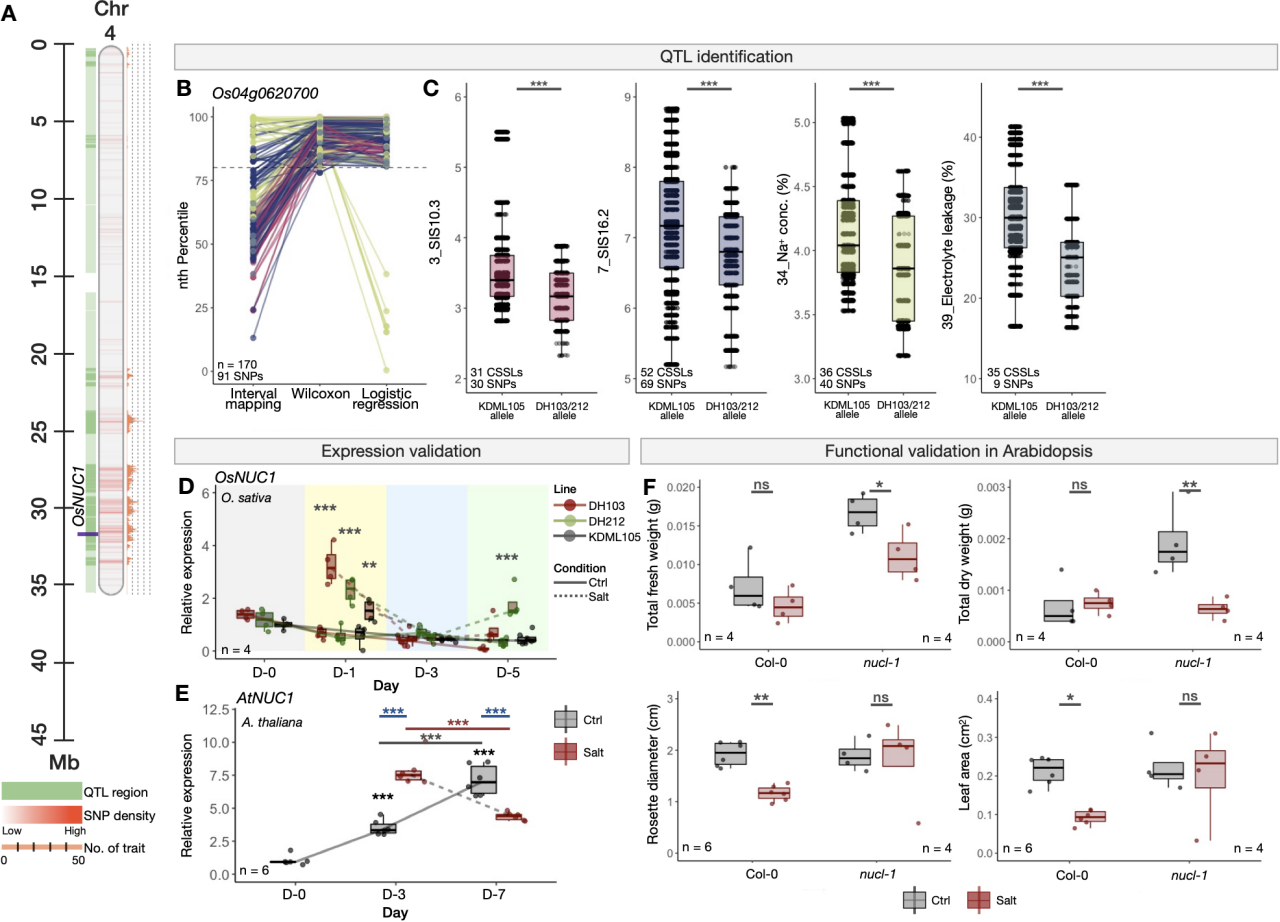
Experimental validations of *OsNUC1* in rice (*O. sativa*) and its orthologue in *A*. *thaliana*. **(A)** Genomic location of *OsNUC1* in the QTL region on Chromosome 4 of *O. sativa* (Nipponbare cv.). **(B)** PR-normalised confidence scores of each SNP position within the *OsNUC1* gene obtained from the three QTL identification methods. Colours representing the four traits are as described in **(C)**. **(C)** Phenotype scores of CSSLs with the alleles from salt-sensitive KDML105, or salt-tolerant DH103/DH212 cultivars in four salinity-related traits (trait no. 3, 7, 34 and 39). Phenotype scores were obtained from Kanjoo et al. (2011) and Pamuta et al. (2014). **(D)** Expression analysis of *OsNUC1* in KDML105, DH103 and DH212 rice cultivars under the control and salt stress (100 mM NaCl) condition. The salt treatment was conducted using 16-day-old rice seedlings. **(E)** Expression analysis of *OsNUC1* orthologous gene in *A*. *thaliana*, *AtNUC1*, under the control and salt stress (100 mM NaCl) condition. The salt treatment was conducted using 10-day-old Col-0 *A*. *thaliana* seedlings. **(F)** Morphological responses, namely total fresh weight, total dry weight, rosette diameter and leaf area of WT (Col-0) and loss-of-function mutant line (*nucl-1*). The experiment was conducted using 7-day-old seedlings and the measurement was done 12 days in the control or salt stress (250 mM NaCl) conditions. Error bars represent standard deviations from four biological replicates. Asterisks (*) represent the significant p-value [one-way ANOVA in **(D)** and **(E)** and t-test in **(F)**] between the control and salt stress conditions. Significance levels: * p < 0.05; ** p < 0.01; *** p < 0.001; ns denotes not significant.

As an example of well-characterised salt-responsive genes, we investigated the expression and functions of *OsNUC1* (**Figure 5**), whose salt-tolerant enhancing function has previously been shown in rice overexpressing *OsNUC1* (Sripinyowanich et al., 2013). Nucleolin is found in the nucleolus and plays an important role in assembly of ribosomal proteins and RNA (Maris et al., 2005; Lunde et al., 2007). In our study, *OsNUC1* was identified as a high-confidence salt-tolerant candidate in a multi-trait QTL region on Chromosome 4 (**Figure 5A**) based on 91 high-confidence SNPs within the region in at least two QTL identification methods (**Figure 5B**). In the four traits that we detected significant SNPs in this region, namely SIS (traits no. 3 and 7), Na^+^ concentration (trait no. 34) and electrolyte leakage (trait no. 39), the CSSLs with the SNP alleles from the salt-tolerant parents (i.e., DH103 and/or DH212) showed higher tolerance levels than those with the alleles from the salt-susceptible parent (i.e., KDML105), that is, lower SIS, cellular Na^+^ concentrations, and electrolyte leakage under salt stress (**Figure 5C**).

We then performed additional expression analysis and showed that *OsNUC1* was up-regulated at an early stage (Day 1) after being treated with a moderate salt stress (100 mM NaCl) in the DH103, DH212 and KDML105 rice cultivars, and particularly higher in the two salt-tolerant lines (**Figure 5D**). Similar trend was seen in the Arabidopsis orthologue *AtNUCL-1 (NUCLEOLIN LIKE 1, AT1G48920)*, whose transcription level was higher in a salt-stress condition as compared to the normal control condition at three days (Day 3) after the treatment, and then decreased to below the control at Day 7 (**Figure 5E**). Functional validation in the lack-of-function Arabidopsis mutant *nucl-1* showed significant reduction of fresh and dry weights in the mutant under the salt stress, whereas such an influence was not seen in wild-type Col-0 plants (**Figures 5F**; **Supplementary Figure S21**). On the contrary, the reductions in rosette leaf diameters and areas observed in Col-0 under salt stress disappeared in *nucl-1*. These together recapitulate the functions of the *NUC1* gene in salinity-related and enhancing tolerance, as seen in Sripinyowanich et al. (2013). Similarly to *OsNUC1*, we also performed expression and functional validations in three other known salt-responsive genes: pyrimidine biosynthesis *OsDHODH1* (*DIHYDROOROTATE DEHYDROGENASE 1*, Os02g0736400, **Supplementary Figure S22**) (Liu et al., 2009); chromatin modification *OsSRWD1* (*SALT RESPONSIVE WD40 PROTEIN 1*, Os08g0497600, **Supplementary Figure S23**) (Huang et al., 2008); ion homeostasis regulation *OsFRO1* (*FERRIC REDUCTASE 1*, Os04g0578600, **Supplementary Figure S24**) (Wang et al., 2013; Muhammad et al., 2018).

**Figure 6:**
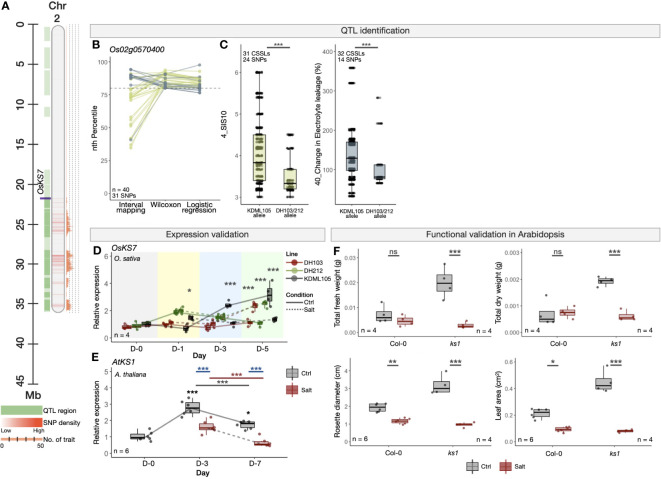
Experimental validations of *OsKS7* in rice (*O. sativa*) and its orthologue in *A*. *thaliana*. **(A)** Genomic location of *OsKS7* in the QTL region on Chromosome 2 of *O. sativa* (Nipponbare cv.). **(B)** PR-normalised confidence scores of each SNP position within the *OsKS7* gene obtained from the three QTL identification methods. Colours representing the four traits are as described in **(C)**. **(C)** Phenotype scores of CSSLs with the alleles from salt-sensitive KDML105, or salt-tolerant DH103/DH212 cultivars in two salinity-related traits (trait no. 4 and 40). Phenotype scores were obtained from Kanjoo et al. (2011) and Pamuta et al. (2014). **(D)** Expression analysis of *OsKS7* in KDML105, DH103 and DH212 rice cultivars under the control and salt stress (100 mM NaCl) condition. The salt treatment was conducted using 16-day-old rice seedlings. **(E)** Expression analysis of *OsKS7* orthologous gene in *A*. *thaliana*, *AtKS1*, under the control and salt stress (100 mM NaCl) condition. The salt treatment was conducted using 10-day-old Col-0 *A*. *thaliana* seedlings. **(F)** Morphological responses, namely total fresh weight, total dry weight, rosette diameter and leaf area of WT (Col-0) and loss-of-function mutant line (*ks1*). The experiment was conducted using 7-day-old seedlings and the measurement was done 12 days in the control or salt stress (250 mM NaCl) conditions. Error bars represent standard deviations from four biological replicates. Asterisks (*) represent the significant p-value [one-way ANOVA in **(D)** and **(E)** and t-test in **(F)**] between the control and salt stress conditions. Significance levels: * p < 0.05; ** p < 0.01; *** p < 0.001; ns denotes not significant.

We also looked into several potential novel salinity-related candidate genes, including *OsKS7* (*ENT-KAURENE SYNTHASE 7*, Os02g0570400) (**Figure 6**), which is known for its role in diterpene phytoalexin biosynthesis, a low molecular weight antimicrobial compound taking part in defence mechanisms to control invading microorganisms such as fungi, bacteria and viruses (Kanno et al., 2006). Up-regulation of *OsKS7* was shown in rice seedlings under UV treatment (Cho et al., 2004). To the best of our knowledge; however, its role on sensing or responding to salt stress has not been yet characterised. The *OsKS7* is located within another multi-trait QTL region on Chromosome 2 (**Figure 6A**), which contains 31 high-confidence SNPs detected by at least two QTL identification methods, linking to two salinity-related traits, namely SIS10 (trait no. 4) and change in electrolyte leakage (trait no. 40) (**Figure 6B**). Similar to the *OsNUC1*-containing genomic region described above, the CSSLs that contain the SNP alleles from DH103 and/or DH212 within the *OsKS7* gene showed superior tolerant characteristics as compared to KDML105, as indicated by lower SIS and change in electrolyte leakage (**Figure 6C**). *OsKS7* has been shown to be transcriptionally up-regulated in rice under salt stress based on the combined publicly available transcriptomes we described earlier (**Supplementary Table S11**).

According to our additional expression analyses (**Figure 6D**), *OsKS7* was down-regulated under salt stress as compared to control at Day 3 and Day 5 in the salt-susceptible KDML105, but the gene was up-regulated in the salt-tolerant DH103 and DH212 at Day 5, suggesting *OsKS7* might enhance salinity tolerance in rice. In Arabidopsis, the expression of *AtKS1 (ENT-KAURENE SYNTHASE 1*, AT1G79460*)*, an orthologue of rice *OsKS7*, was down-regulated in wild-type Col-0 under salt stress (**Figure 6E**), corresponding to the reduction of rosette leaf diameters and areas in Col-0 (**Figures 6F** and **Supplementary Figure S12**). This, together with further loss of fresh and dry weights and even greater reductions of leaf diameters and areas in the *ks1* mutant reinforce the role of *OsKS7/AtKS1* in promoting salt tolerance in rice and Arabidopsis. Other new candidate genes also being tested for their functional roles in salinity responses are a hydrolase gene with unknown function (Os02g0706900, **Supplementary Figure S25**); a growth and development regulator *OsEXPA21 (ALPHA-EXPANSIN 21*, Os02g0377100, **Supplementary Figure S26**, see also Extended Results).

## Discussion

Elevated salt concentration in soil has detrimental effects on multiple aspects of plant growth and development, presenting a significant risk to agricultural production. Therefore, enhancing the ability of agriculturally important crops to withstand high salt levels holds immense importance. Similar to other abiotic stresses, the mechanisms that plants employ to sense and respond to salinity are complex and known to involve multiple genes and biological pathways (Flowers, 2004; Pires et al., 2015; Qin et al., 2020). Hence, investigating one or a few salt-tolerant traits alone is unlikely to offer a comprehensive understanding of the complex and interwinding mechanisms involved in salt-stress responses.

Thanks to advances in genetic marker identification and next-generation sequencing, recent association mapping studies have been able to examine the relationships between several stress-related traits and genomic loci, e.g., Julkowska et al. (2016), Tsai et al. (2019) and Zhao et al. (2022). Nevertheless, earlier QTL and GWAS studies typically focus on a specific set of related traits of interest. For instance, in recent QTL studies of salt-responsive traits, earlier studies focused on photosynthetic efficiency under salinity stresses e.g., Liu et al. (2017), Sun et al. (2019) and Tsai et al. (2019). Homeostasis of intracellular Na^+^ and K^+^ levels is another important aspect of plant responses to salt stress, and this was the main focus of several earlier QTL studies, e.g., Wang et al. (2012), Krishnamurthy et al. (2020) and Nakhla et al. (2021). Each of these traits reflect different aspects of abiotic stresses, which might be linked to the same or different loci and genes (Liu et al., 2009; Genc et al., 2010). Thus, it would be of important interest to standardise, integrate and unbiasedly compare multiple traits from multiple studies, into a single comprehensive loci-trait and trait-trait investigation.

In this study, we have developed a heuristic framework for systematic integration and comprehensive evaluation of multiple stress-related traits, using physiology and phenotype information obtained in three different QTL studies performed in rice (Kanjoo et al., 2011; Pamuta et al., 2014; Nounjan et al., 2016), as a model to illustrate our approach (**Figure 1**). Under salt stress, rice seedlings showed clear morphological and physiological differences between the tolerant and susceptible cultivars (see **Supplementary Figure S12**). These include higher SISs, higher root to shoot ratio, lower plant survival and dry weights, lower total chlorophyll content, lower proline content, higher Na^+^ to K^+^ ratio, and higher electrolyte leakage that are all observed in the susceptible cultivar KDML105.

To minimise potential technical biases resulting from different QTL identification methods, we combined results from three QTL identification methods, namely interval mapping, Wilcoxon signed-rank test, logistic regression, and assigned the confidence levels of the SNPs and loci associated with the traits of interest based on the agreement between the methods (**Figure 2**, **Supplementary Figure S3**). Combining and comparing confidence scores from different QTL identification methods is by no means straightforward, as each of them exhibit distinct ranges of values (**Supplementary Figures S6**, **S12**, **S13**). By transforming the QTL confidence scores of respective methods into the PR-normalised scores, we were able to prioritise, investigate, and compare the SNPs and loci that were identified by different QTL methods (**Figure 2**; **Supplementary Figure S13**). Such implementation of the PR normalisation to the QTL confidence scores had not been done before, as far as we know, but a similar approach has successfully been used based on the phenotype and trait scores in earlier studies (Zou et al., 2003; Goh and Yap, 2009).

Our comparative analysis of multi-traits and loci also allows us to obtain a bird’s eye view of the relationships between several phenotypic and physiological properties, as well as to characterise trait-specific and multi-trait loci (**Figures 2**, **3**). Indeed, analyses of the links between multiple traits have been performed in earlier studies, using the correlations between the phenotype and trait properties e.g., Ninsuwan et al. (2013), Wang et al. (2013) and Habila et al. (2022). However, for the first time in this work, we also examined the correlations between the traits in terms of the similarities of QTL confidence scores assigned to all the SNPs across the genome (**Figure 4**). This allowed us to investigate two or more traits that may exhibit high correlations in terms of phenotype scores among the CSSLs, but the traits might be mapped to different genomic loci, and hence show low or negative correlations of QTL confidence scores (for instance, between period of plant survival scores or PPS (trait no. 16) vs’ dry weights (trait no. 25 and 27), as seen in **Figure 4**). On the other hand, we also observed high correlations of QTL confidence scores between traits, despite their phenotype scores not exhibiting high correlations among the CSSLs, suggesting these traits are linked genetically or regulated by the same or overlapping genes (for instance, between chlorophyll content (trait no. 37-38) vs’ dry weights (trait no. 25-30); and between SIS (trait no. 1-15 and 17-24) vs’ proline content (trait no. 41), see also **Figure 4**).

In addition to establishing the analytic framework for combining and analysing multiple phenotypic and physiological traits, we have showcased its practical applications in identifying previously known salinity-related SNPs and genes, as well as discovering new candidates that can further be tested and evaluated (see complete lists of candidates in **Supplementary Table S11** and validated genes in **Supplementary Table S13**). We have carefully investigated and validated several candidate genes from various biological pathways, including the genes whose functions are directly involved in growth and development. In the presence of salt stress, cell division and expansion are hindered because the priority switches to coping with osmotic pressure and water loss (Munns and Tester, 2008; Roy et al., 2014). Here, we have recapitulated the role of *OsNUC1* in promoting salt tolerance as observed by Sripinyowanich et al. (2013). Another potential candidate known for its involvement in growth and development processes is *OsEXPA21*, a gene known for its role in loosening plant cell walls and cell enlargement (Cosgrove, 2000). The gene is located in the QTL regions identified in the traits directly linked to growth and development such as lower SIS (trait no. 1 and 46), and higher survival rates (trait no. 32 observed in CSSLs with DH103/212 alleles) (see also **Supplementary Figure S26** and Extended Results).

Ion transport and homeostasis regulation is another important aspect that determines salt tolerance in plants, as it mediates translocation of various ions including Na^+^, through the xylem, vacuolar sequestration, as well as ion channels to name a few (Wu, 2018). Using our framework, the gene *OsFRO1*, which is known for its role in Fe homeostasis between cytoplasm and vacuole (Moore et al., 2014; Li et al., 2019), was identified as a high-confidence salinity-related candidate in five traits including Na^+^ concentrations (trait no. 34) (**Supplementary Figure S24**). Other genes identified as salinity-related genes in this study that are involved in this pathway include *OsNHX1* (*Na^+^/H^+^ ANTIPORTER*, Os07g0666900), which is localized in the tonoplast membrane and functions in compartmentation of Na^+^ from cytosol into the vacuoles in exchange for H^+^ (Fukuda et al., 2004), and *OsACA11* (*CA^2+^-ATPASE 11*, Os04g0605500) (Huda et al., 2013).

The salinity tolerance of rice can be influenced by various biochemical compounds, including osmoprotectants, signalling molecules, and polyamines (Ganie et al., 2019). These biochemical protectants can mitigate the effect of salinity stress by regulating osmotic balance, and hence improving photosynthesis, seed germination and antioxidation (Singh et al., 2022). Examples of salinity-related genes involved in osmoprotectant regulation characterised using our framework include *OsKS7*, which generates a polyamine compound taking part in defence mechanisms (Kanno et al., 2006) (**Figure 6**). Another notable candidate is *OsDHODH1*, whose role in promoting salt tolerance was described by Liu and coworkers (Liu et al., 2009) (see **Supplementary Figure S22** and Extended Results).

We have also identified multiple signal transduction genes as salinity-related candidates. For instance, we observed *OsSRWD1*, being characterised as a candidate in eight traits relating to salt-tolerant phenotypes, and it was up-regulated as early as one and three days after the salt stress treatments in rice and in Arabidopsis, respectively (**Supplementary Figures S23B–E**). This falls in line with an earlier work by Huang et al. (2008), which demonstrated that the WD40 protein subfamily promotes salt tolerance in the seedlings of IR64 rice under salt stress. Other candidates salinity-related genes with signal transduction functions that were identified in this study include *OsMSR2* (*MULTI-STRESS-RESPONSIVE GENE 2*, Os01g0955100), as previously characterised by Khrueasan et al. (2019) and Xu et al. (2013), protein kinases *OsCDPK7* (*CALCIUM-DEPENDENT PROTEIN KINASE 7*, Os04g0584750) (Saijo et al., 2000), *OsCPK12* (*CALCIUM-DEPENDENT PROTEIN KINASE* 12, Os04g0560600) (Asano et al., 2012) and *OsMKK4* (*MAP KINASE KINASE 4*, Os02g0787300) (Kumar et al., 2008). Note that a complete list of candidates with the functions relating to other biological processes such as reactive oxygen species (ROS) regulation, photosynthesis, and electron transportation, can be found in **Supplementary Table S11**.

Our framework offers a robust approach for systematic integration and comprehensive characterisation of multiple stress-related traits, using a combined dataset of 46 salinity-related traits from three independent studies that shared the same CSSL populations as a model. The total number of CSSLs analysed in this study is 135, which is a suitable size for bi-parental QTL mapping studies (Collard et al., 2005), and within the same range as in other recent QTL studies e.g., Tiwari et al. (2016) and Kulkarni et al. (2020). In terms of number of polymorphic markers, there are 3,171 informative SNPs out of 18,181 total SNPs between DH212 and KDML105; and 7,714 informative out of 15,562 total SNPS between DH103 and KDML105, in the populations used as a model in this study. While it is known that a greater number of informative SNPs will provide a higher mapping resolution, the SNP numbers used in our study are comparable to what were used in recent QTL studies in rice, e.g., Tiwari et al. (2016), Kulkarni et al. (2020) and Zhao et al. (2020). These together suggest that the representative dataset used to develop the framework is suitable and provides sufficient statistical power and resolution. With larger numbers of individuals and SNPs, we expect the framework to provide better accuracy and coverage in identifying known and novel abiotic-related genes. Another potential limitation of this work is the QTL genomic markers used as the benchmarks for optimising the framework, which is currently based on earlier studies on the same CSSL populations. Expanding the list of benchmarking salinity-related genomic regions and genes could potentially improve the coverages of new candidate searches.

Taken together, our work not only introduces a novel approach for conducting comparative analyses of multiple QTLs, but also provides a list of candidate genes and readily testable hypotheses for salinity-related mechanisms across various biological pathways. This, in turn, will contribute to expedite the enhancement of crop resilience to salinity and other abiotic stresses associated with global climate changes.

## Data availability statement

The original contributions presented in the study are included in the article/**Supplementary Files**, further inquiries can be directed to the corresponding author/s.

## Author contributions

SP: Conceptualization, Writing – original draft, Writing – review & editing, Data curation, Formal analysis, Methodology, Validation, Visualization. NN: Writing – review & editing, Resources. PT: Conceptualization, Resources, Writing – review & editing. MS: Conceptualization, Funding acquisition, Resources, Writing – original draft, Writing – review & editing. VC: Conceptualization, Funding acquisition, Investigation, Project administration, Supervision, Writing – original draft, Writing – review & editing.
