# Supplementary material for: Comparative quantitative trait loci analysis framework reveals relationships between salt stress responsive phenotypes and pathways
Source: Front Plant Sci. 2024 Feb 23;15:1264909. doi: 10.3389/fpls.2024.1264909 (PMC10920293; doi:10.3389/fpls.2024.1264909)
Supplement: Supplementary file 1 [file DataSheet_1.zip › Extended Results.pdf]

frontiers

*Extended Results*

# Comparative Quantitative Trait Loci Analysis Framework Reveals Relationships between Salt-Stress Responsive Phenotypes and Pathways

**Sunadda Phosuwan[1,2], Noppawan Nounjan[3], Piyada Theerakulpisut[4], Meechai Siangliw[5,*] and Varodom Charoensawan[2,6,7,8,9,10*]**

[1]Doctor of Philosophy Program in Biochemistry (International Program), Faculty of Science, Mahidol University, Bangkok, Thailand

[2]Department of Biochemistry, Faculty of Science, Mahidol University, Bangkok, Thailand

[3]Biodiversity and Environmental Management Division, International College, Khon Kaen University, Khon Kaen, Thailand

[4]Salt-tolerant Rice Research Group, Department of Biology, Faculty of Science, Khon Kaen University, Khon Kaen, Thailand

[5]National Center for Genetic Engineering and Biotechnology (BIOTEC), Pathum Thani, Thailand.

[6]Integrative Computational BioScience (ICBS) center, Mahidol University, Nakhon Pathom, Thailand

[7]Division of Medical Bioinformatics, Research Department, Faculty of Medicine Siriraj Hospital, Mahidol University, Bangkok, Thailand

[8]Department of Biochemistry, Faculty of Medicine Siriraj Hospital, Mahidol University, Bangkok, Thailand

[9]Siriraj Genomics, Faculty of Medicine Siriraj Hospital, Mahidol University, Bangkok, Thailand

[10]School of Chemistry, Institute of Science, Suranaree University of Technology, Nakhon Ratchasima, Thailand

*** Correspondence:**
Varodom Charoensawan and Meechai Siangliw
varodom.cha@mahidol.ac.th, meechai@biotec.or.th

***Note for Extended Results***

This Supplementary Materials provides additional descriptions of candidate genes that were characterised by the analytic pipeline presented in the study by Phosuwan et al. (2024), together with their biological functions if available.

***Comprehensive trait-loci relationships detected by different QTL methods***

Among the salinity-related SNPs and genes identified by this approach, we observed several high-confidence loci and SNPs found by all the three QTL methods black boxes in Figure 2), as seen in earlier studies (as summarised in Table S2). For instance, we observed the SNPs linked to SIS on Chromosome 1 (see Boxes 1, 2 and 4 indicating the positions of SNPs associated with the trait numbers 6, 11, 15, 14, and 20 in Figure 2), as previously characterised by Khrueasan et al. (2013), Chutimanukul et al. (2018b) and Khrueasan et al. (2020). Examples of these salinity-related candidate genes within these regions are ribosomal proteins *OsRPL27* (*50S RIBOSOMAL PROTEIN L27,* Os01g0924000) and *OsRPL34* (*50S RIBOSOMAL PROTEIN L34*, Os01g0805000), *DUF3007* (*PROTEIN OF UNKNOWN FUNCTION DUF3007*, Os01g0805200) and *OsNDHO* (*NAD(P)H:PLASTOQUINONE DEHYDROGENASE COMPLEX SUBUNIT O*, Os01g0959900), as indicated by the genomic locations i, iii, ii and vi in Figure 2, respectively. Also on Chromosome 1, our framework was able to identify the known photosynthesis-related salinity-related gene *OsMSR2* (*MULTI-STRESS-RESPONSIVE GENE 2*, Os01g0955100) (Gene v and Box 2 - trait no. 37 in this study) (Khrueasan et al., 2019), and the gene relating to seedling growth *OsIRO2* (*IRON-RELATED TRANSCRIPTION FACTOR 2*, Os01g0952800) (Gene iv and Boxes 1 and 3 - trait no. 25-30 and 33) (Chutimanukul et al., 2018b; Khrueasan et al., 2019). On Chromosome 8, we detected SNPs linked to SIS (Boxes 15 and 18 - trait no. 8 and 22), as previously identified by Kanjoo et al. (2011), SNPs linked to dry weights (Box 14 - trait no. 25-30), and the total chlorophyll content-related gene *Os08g0526300* (Gene x and Box 16 - trait no. 37), as previously identified by Nounjan et al. (2016).

When we expanded our search criteria to cover SNPs identified by at least two QTL identification methods (red, green, and blue boxes in Figure 2), we recovered more SNPs linked to SIS, such as on Chromosome 7 (Box 13 - trait no. 6-8 and 10) and on Chromosome 9 (Boxes 21 and 22 - trait no. 8 and 22), as characterised by Kanjoo et al. (2011), as well as SNPs on Chromosome 8 which are related to dry weights (Box 14 - trait no. 25, 27 and 29), SIS (Box 19 and trait no. 1 and 46) and photosynthesis adaptation (Boxes 17 and 20 - trait no. 37 and 38), as proposed by Nounjan et al. (2016). These exemplify the ability of our integrative framework in terms of re-discovering characterised salinity-related QTLs and genes from earlier studies.

We also observed several high-confidence SNPs detected by at least two QTL mapping methods that did not correspond to a known salt-stress loci characterised from these particular CSSL populations, but have been demonstrated to be linked to salinity in other studies conducted

in independent populations and studies. For instance, we found SNPs associated with SIS (Boxes 7 and 8 - trait no. 5, 7 and 10) and ion balance adaptation (Boxes 10 and 11 - trait no. 34, 39 and 40) on Chromosome 4 (see Figure 2), being linked to a salt-responsive gene *OsNUC1* (*NUCLEOLIN 1*, Os04g0620700) (see Gene ix), as previously identified by Sripinyowanich et al. (2013). Similarly, more SNPs associated with SIS (Box 23 - trait no. 19, 21 and 22) can be linked to the cellular homeostasis and salt-responsive gene *OsTRE1* (*TREHALASE 1*, Os10g0521000) (see Gene xi), as described by Islam et al. (2019). We also observed the intracellular iron homeostasis-related *OsFRO1* (*FERRIC REDUCTASE 1*, Os04g0578600) (Gene viii on Chromosome 4), which was previously characterised as salinity-related genes (Wang et al., 2013; Muhammad et al., 2018), being identified by our framework to be associated with SIS (Boxes 9 and 12 - trait no. 45 and 46), survival rates (Box 9 - trait no. 31 and 32) and $Na^+$ concentration (Box 9 - trait no. 34).

In addition to this, we have found high-confidence QTL regions with novel candidate salinity-related genes, of which to the best of our knowledge, their salt-responsive functions have not been characterised before. These include the gene phytoalexin biosynthesis-related *OsKS7* (*ENT-KAURENE SYNTHASE 7*, Os02g0570400) (see Gene vii on Chromosome 2), which is associated with SIS10 (Box 5 - trait no. 4) and electrolyte leakage (Box 6 - trait no. 39).

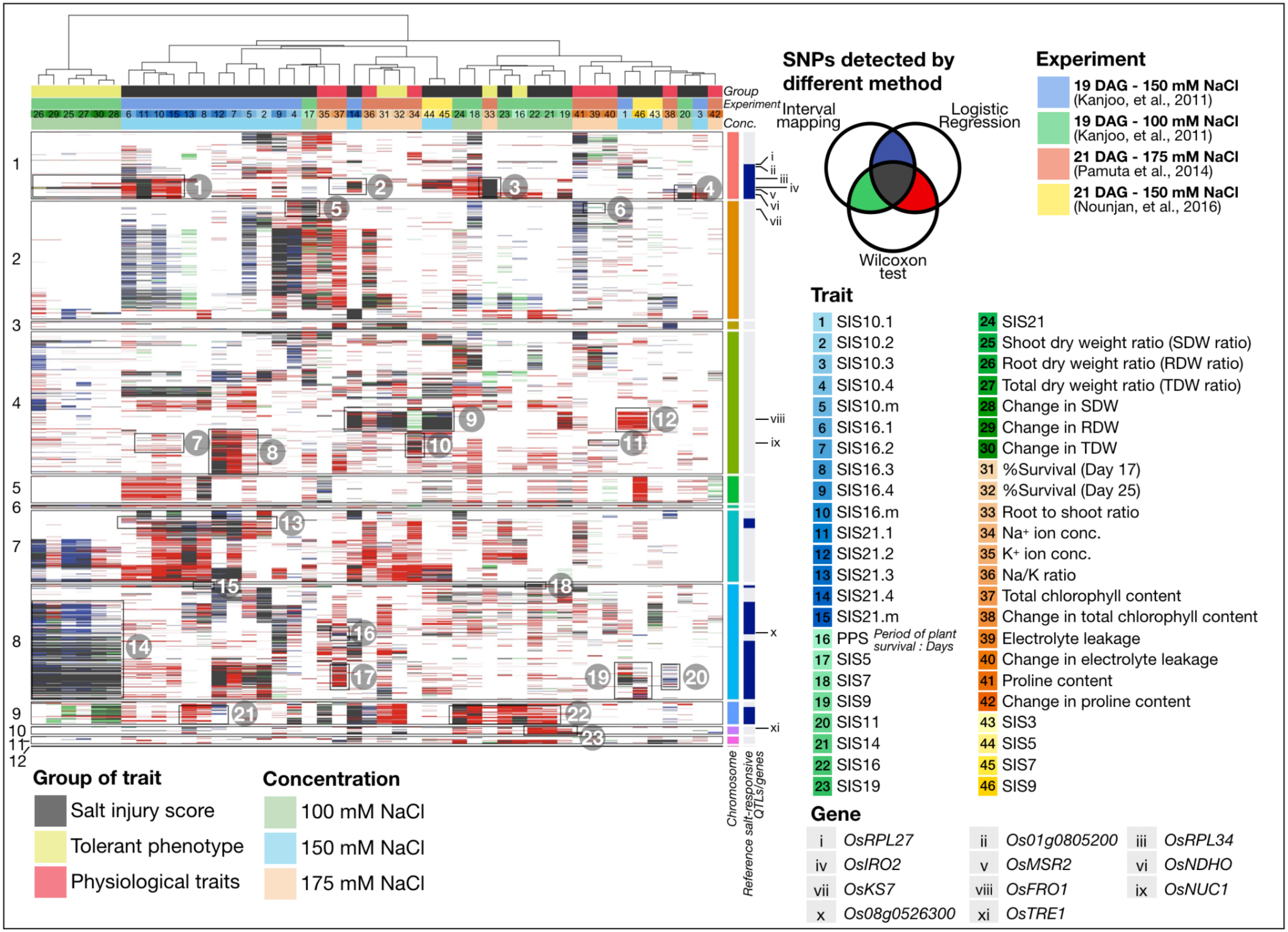
Group
Experiment
Conc.
SNPs detected by different method
Interval mapping
Logistic Regression
Wilcoxon test
Experiment
19 DAG - 150 mM NaCl (Kanjoo, et al., 2011)
19 DAG - 100 mM NaCl (Kanjoo, et al., 2011)
21 DAG - 175 mM NaCl (Pamuta et al., 2014)
21 DAG - 150 mM NaCl (Nounjan, et al., 2016)
Trait
1 SIS10.1
2 SIS10.2
3 SIS10.3
4 SIS10.4
5 SIS10.m
6 SIS16.1
7 SIS16.2
8 SIS16.3
9 SIS16.4
10 SIS16.m
11 SIS21.1
12 SIS21.2
13 SIS21.3
14 SIS21.4
15 SIS21.m
16 PPS Period of plant survival : Days
17 SIS5
18 SIS7
19 SIS9
20 SIS11
21 SIS14
22 SIS16
23 SIS19
24 SIS21
25 Shoot dry weight ratio (SDW ratio)
26 Root dry weight ratio (RDW ratio)
27 Total dry weight ratio (TDW ratio)
28 Change in SDW
29 Change in RDW
30 Change in TDW
31 %Survival (Day 17)
32 %Survival (Day 25)
33 Root to shoot ratio
34 Na+ ion conc.
35 K+ ion conc.
36 Na/K ratio
37 Total chlorophyll content
38 Change in total chlorophyll content
39 Electrolyte leakage
40 Change in electrolyte leakage
41 Proline content
42 Change in proline content
43 SIS3
44 SIS5
45 SIS7
46 SIS9
Gene
i OsRPL27
ii Os01g0805200
iii OsRPL34
iv OsIRO2
v OsMSR2
vi OsNDHO
vii OsKS7
viii OsFRO1
ix OsNUC1
x Os08g0526300
xi OsTRE1
Group of trait
Salt injury score
Tolerant phenotype
Physiological traits
Concentration
100 mM NaCl
150 mM NaCl
175 mM NaCl
Chromosome
Reference salt-responsive QTLs/genes

**Figure 2 Heatmap summarising high-confidence SNPs and salinity-related gene candidates identified in 46 salt-responsive traits**. High-confidence SNPs were characterised if they are present in at least 5 CSSLs and passed the 80th percentile confidence scores in two or three QTL identification methods, as indicated in the heatmap in 'blue-green-red' or 'black', respectively (see Methods for more details). Columns and rows represent salinity-related traits and SNPs across the chromosome numbers indicated on the left of the heatmap. Colour codes of the traits, experimental settings and salt concentrations, examples of salinity-related QTL/genes and genes described in the texts are all indicated on the right of the heatmap.

***Investigating trait-specific and multi-trait loci associated with salt-stress phenotypes***

Most of high-confidence QTLs in this study were linked to more than one groups of salinity-related traits (indicated by grey lines in Circos plots, Figure 3, see high-resolution version in Supplementary Materials) (see also a complete list of QTL-trait relationships in Table S10). For instance, Chromosomes 1 Region A was associated with several types of traits including SIS (trait no. 2, 9 and 17), $K^+$ concentration (trait no. 35), electrolyte leakage (trait no. 39 and 40) and proline content (trait no. 41). Within this region, we found examples of known salt-responsive genes such as *OsIF* (*INTERMEDIATE FILAMENT*, Os01g0292700),which is related to seedling growth, electrolyte leakage and proline content adaptation under salinity as shown in a previous study (Soda et al., 2016). We also observed another multi-trait locus on Chromosome 1 Region E (Figure 3), being linked to SIS of different plant stages (trait no. 3, 6, 9, 11, 14, 18-20 and 43-45), dry weights (trait no. 28-30), root to shoot ratio (trait no. 33), and change in total chlorophyll (trait no. 38). As an example, within this region we found previously characterised salinity-related genes such as *OsNPC2* (*NON-SPECIFIC PHOSPHOLIPASE C2*, Os01g0955000) (Ngo et al., 2019; Campo and San Segundo, 2020), *OsMSR2* (*MULTI-STRESS-RESPONSIVE GENE 2*, Os01g0955100 (Khrueasan et al., 2019), and *OsASR3* (*ABSCISIC ACID-STRESS-RIPENING-INDUCIBLE 3*, Os01g0959100) (Joo et al., 2013). Other multi-trait loci and genes of interest were also observed in Chromosomes 2 *OsKS7,* Os02g0706900), 3 (*OsEXPA21*, Os03g0377100), 4 (*OsFRO1*, Os04g0578600 and *OsNUC1*, Os04g0620700) and 8 (*OsSRWD1*, Os08g0497600) (see Table S10), for instance. Additional salinity-related candidate genes are described in Tables S11 and S13.

Our comparative analysis also revealed several genomic regions and candidate genes specifically associated with certain stress phenotypes in this study, or "trait-specific gene". To illustrate this point, we discovered specific regions linked to particular traits, including in Chromosome 1 Region B linking to proline content (trait no. 41). This genomic region contains proline production-related genes under salt stress in plants such as *OsGLT1* (*NADH-DEPENDENT GLUTAMATE SYNTHASE 1*, Os01g0681900) and glutathione transferase genes *OsGSTU39* (*TAU GLUTATHIONE S-TRANSFERASE 39*, Os01g0692100) and *OsGSTU40* (*TAU GLUTATHIONE S-TRANSFERASE 40*, Os01g0692000) (Silveira et al., 2003; Kumar and Trivedi, 2018). In Region C of Chromosome 1, which was specifically linked to SIS16 (trait no. 6), we found *OsSUVH7* (*SUVH HISTONE METHYLTRANSFERASE 7*, Os01g0811300), which has been shown to be associated with seedling growth adaptation under salinity (Wang et al., 2020). On Chromosome 1 Region D, which was linked to "root to shoot ratio" (trait no. 33), we found the oxidative stress related gene *OsSPL2* (*SQUAMOSA PROMOTER-BINDING-LIKE 2*, Os01g0922600), and cytokinin receptor *OsHK3* (*HISTIDINE KINASE 3*, Os01g0923700), which have been shown to be associated with the development of the root and aerial tissues in earlier studies (Ito and Kurata, 2006; Yue et al., 2017). The full list of multi-trait and trait-specific loci can be found in Table S10 and the candidate genes within the loci are listed in Table S11.

***Novel salinity-related genes from various biological processes and functions***

In addition to establishing the analytic framework for combining and analysing multiple phenotypic and physiological traits, we have showcased its practical applications in identifying previously known salinity-related SNPs and genes, as well as discovering new candidates that can further be tested and evaluated (see a complete list in Table S11). Here, we described salt-responsive characteristics of selected candidate genes based on their biological functions, and experimental validations of salinity-related candidates in rice and *Arabidopsis thaliana.*

*Growth and development*

In the presence of salt stress, cell division and expansion are hindered because the priority switches to coping with osmotic pressure and water loss (Munns and Tester, 2008; Roy et al., 2014). Here, we have recapitulated the role of *OsNUC1* (*NUCLEOLIN 1*, Os04g0620700) in promoting salt tolerance as observed by Sripinyowanich and colleagues (Sripinyowanich et al., 2013). In our study, *OsNUC1* was identified as a high-confidence salinity-related candidate in four different traits, and its expression was up-regulated under salinity stress in young rice and Arabidopsis seedlings, and the loss-of-function of Arabidopsis orthologue mutant led to restricted growth in terms of biomass and leaf areas (Figure 5). Another potential candidate known for its involvement in growth and development processes is *OsEXPA21* (*ALPHA-EXPANSIN 21*, Os03g0377100), a gene known for its role in loosening plant cell walls and cell enlargement (Cosgrove, 2000). The gene is located in the QTL regions identified in the traits directly linked to growth and development such as lower SIS (trait no. 1 and 46), and higher survival rates (trait no. 32 observed in CSSLs with DH103/212 alleles) (Figure S26). The gene has also been validated for its differential expression in salinity vs' control conditions, and for the impact on plant growth in a lack-of-function mutant vs' WT control (Table S13 and Figure S26).

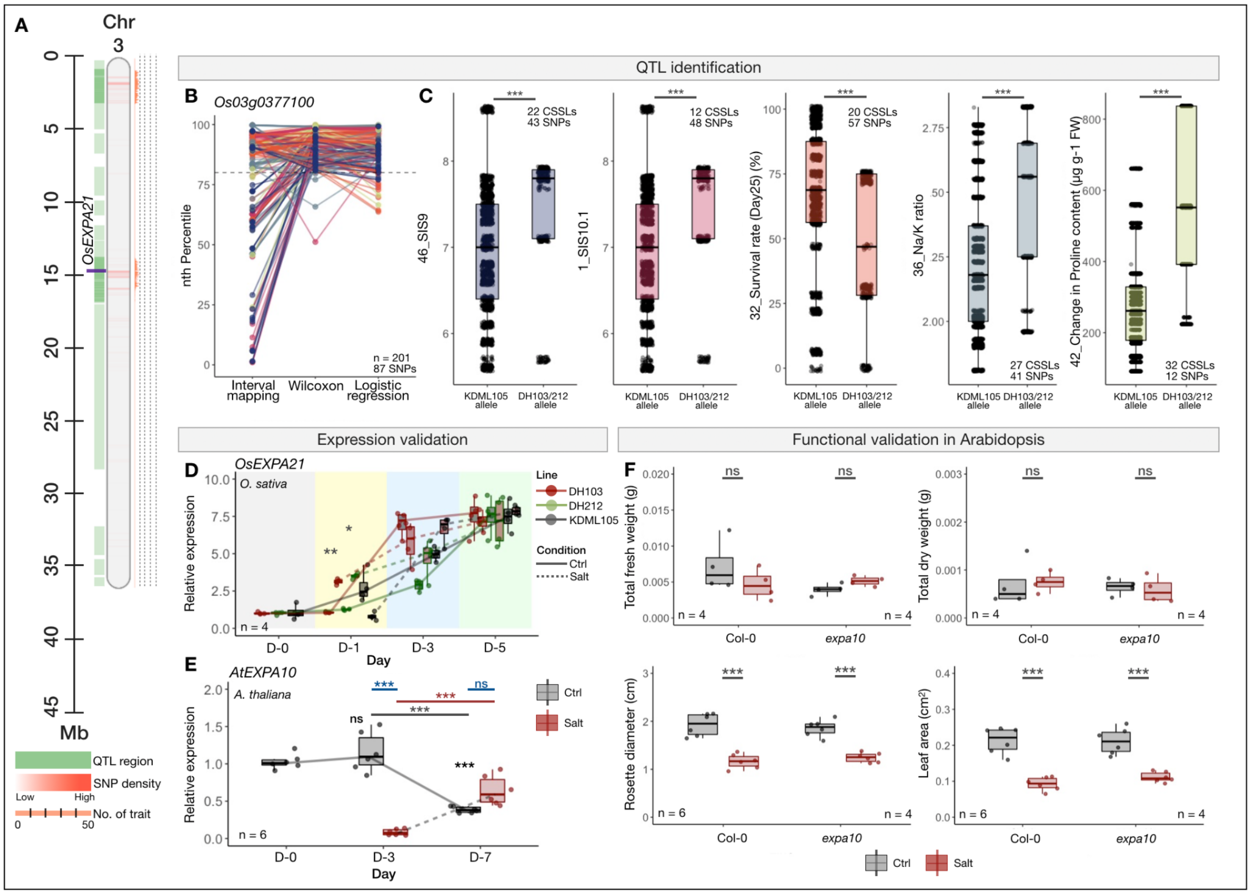


**Figure S26.** Experimental validations of *OsEXPA21* in rice (*O. sativa*) and its orthologue in Arabidopsis (*A. thaliana*). (A) Genomic location of *OsEXPA21* in the QTL region on Chromosome 2 of *O. sativa* (Nipponbare cv.). (B) PR-normalised confidence scores of each SNP position within the *OsEXPA21* gene obtained from the three QTL identification methods. Colours representing the four traits are as described in C. (C) Phenotype scores of CSSLs with the alleles from salt-susceptible KDML105, or salt-tolerant DH103/DH212 cultivars in five salt-responsive traits (trait no. 1, 32, 36, 42 and 46). Phenotype scores were obtained from Kanjoo et al. (2011), Pamuta et al. (2014) and Nuanjan et al. (2016). (D) Expression analysis of *OsEXPA21* in KDML105, DH103 and DH212 rice cultivars under the control and salt stress (100 mM NaCl) condition. The salt treatment was conducted using 16-day-old rice seedlings. (E) Expression analysis of *OsEXPA21* orthologous gene in Arabidopsis, *AtEXPA10*, under the control and salt stress (100 mM NaCl) condition. The salt treatment was conducted using 10-day-old Col-0 Arabidopsis seedlings. (F) Morphological responses, namely total fresh weight, total dry weight, rosette diameter and leaf area of WT (Col-0) and loss-of-function mutant line (*expa10*). The experiment was conducted using 7-day-old seedlings and the measurement was done 12 days in the control or salt stress (250 mM NaCl) conditions. Error bars represent standard deviations from four biological replicates. Asterisks (*) represent the significant p-value (one-way ANOVA in D and E and t-test in F) between the control and salt stress conditions.

*Ion transport and homeostasis*

Ion transport and homeostasis regulation is another important aspect that determines salt tolerance in plants, as it mediates translocation of various ions including $Na^+$, through the xylem, vacuolar sequestration, as well as ion channels to name a few (Wu, 2018). Using our framework, the gene *OsFRO1* (*FERRIC REDUCTASE 1*, Os04g0578600), which is known for its role in Fe homeostasis between cytoplasm and vacuole (Moore et al., 2014; Li et al., 2019), was identified as a high-confidence salinity-related candidate in five traits including $Na^+$ concentrations (trait no. 34) (Figures S2). Similar to what was observed by Muhammad and coworkers (Muhammad et al., 2018), we saw that the expression of *OsFRO1* was up-regulated in the susceptible rice cultivar KDML105 at one day after salinity treatment, as compared to in the control, but our extended time points at three and five days post treatment showed the opposite. Interestingly, at these later stages, *OsFRO1* was transcribed under salinity stress lower than in the control in KDML105, as well as the two salt-tolerant cultivars, DH102 and DH103 (Figure S24D), and the same was seen in Arabidopsis (Figure S24E). This suggests that low expression of the ferric reductase oxidase gene might be linked to salt tolerance due to the crosstalks between salinity and heavy metal responsive pathways, which might activate different signalling cascades and converge to give rise overlapping outcomes (Knight and Knight, 2001; Hamed et al., 2013). Other genes identified as salinity-related genes in this study that are involved in this pathway include *OsNHX1* (*$Na^+$/$H^+$ ANTIPORTER*, Os07g0666900), which plays a role in exporting $Na^+$ from the cell in exchange for $H^+$ (Fukuda et al., 2004), and *OsACA11* (*$CA^{2+}$-ATPASE 11*, Os04g0605500) (Huda et al., 2013).

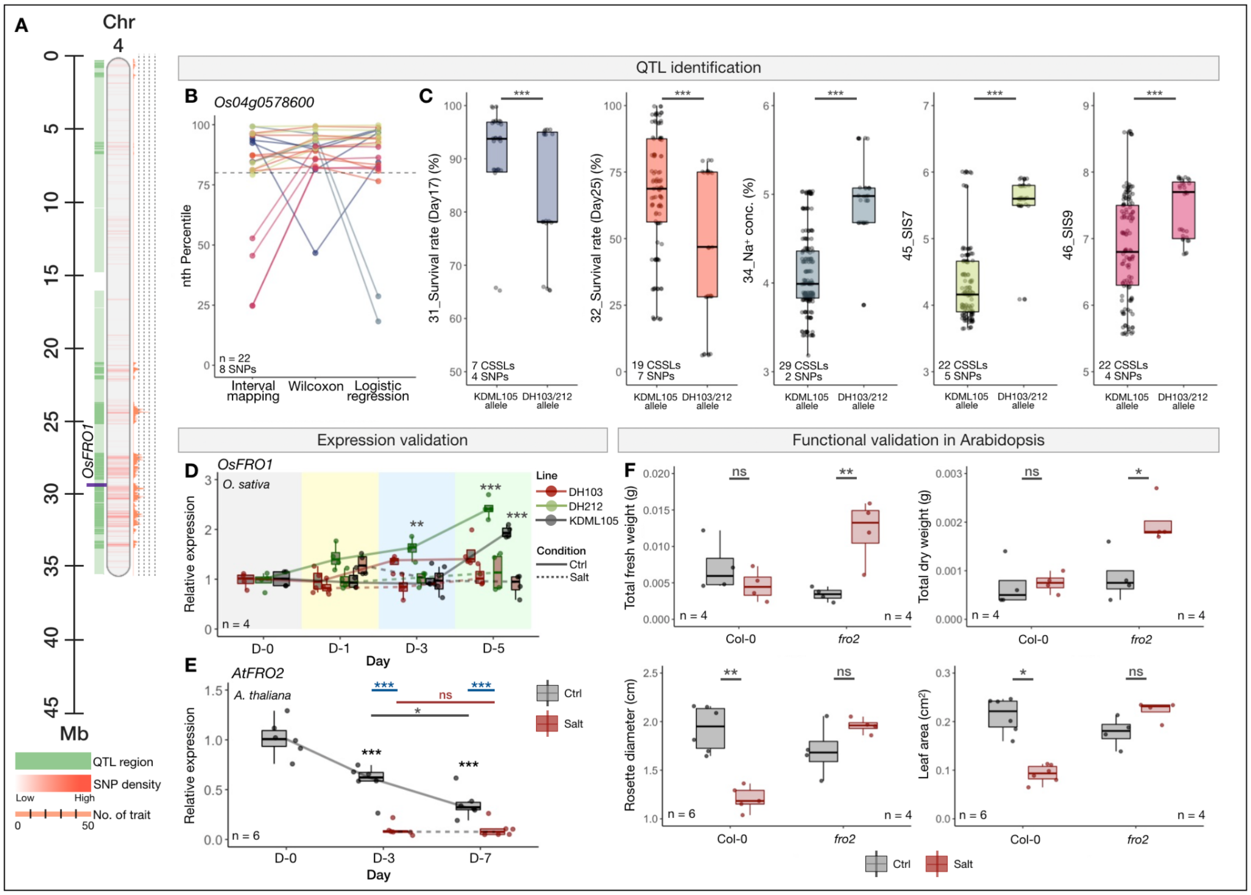


**Figure S24.** Experimental validations of *OsFRO1* in rice (*O. sativa*) and its orthologue in Arabidopsis (*A. thaliana*). (A) Genomic location of *OsFRO1* in the QTL region on Chromosome 4 of *O. sativa* (Nipponbare cv.). (B) PR-normalised confidence scores of each SNP position within the *OsFRO1* gene obtained from the three QTL identification methods. Colours representing the four traits are as described in C. (C) Phenotype scores of CSSLs with the alleles from salt-susceptible KDML105, or salt-tolerant DH103/DH212 cultivars in five salt-responsive traits (trait no. 31, 32, 34, 45 and 46). Phenotype scores were obtained from Pamuta et al. (2014) and Nuanjan et al. (2016). (D) Expression analysis of *OsFRO1* in KDML105, DH103 and DH212 rice cultivars under the control and salt stress (100 mM NaCl) condition. The salt treatment was conducted using 16-day-old rice seedlings. (E) Expression analysis of *OsFRO1* orthologous gene in Arabidopsis, *AtFRO2*, under the control and salt stress (100 mM NaCl) condition. The salt treatment was conducted using 10-day-old Col-0 Arabidopsis seedlings. (F) Morphological responses, namely total fresh weight, total dry weight, rosette diameter and leaf area of WT (Col-0) and loss-of-function mutant line (*fro2*). The experiment was conducted using 7-day-old seedlings and the measurement was done 12 days in the control or salt stress (250 mM NaCl) conditions. Error bars represent standard deviations from four biological replicates. Asterisks (*) represent the significant p-value (one-way ANOVA in D and E and t-test in F) between the control and salt stress conditions.

## *Regulation of biochemical compounds*

The salinity tolerance of rice can be influenced by various biochemical compounds, including osmoprotectants, signalling molecules, and polyamines (Ganie et al., 2019). These biochemical protectants can mitigate the effect of salinity stress by regulating osmotic balance, and hence improving photosynthesis, seed germination and antioxidation (Singh et al., 2022). Examples of salinity-related genes involved in osmoprotectant regulation characterised using our framework include *OsKS7* (*ENT-KAURENE SYNTHASE 7*, Os02g0570400), that generating a polyamine compound taking part in defence mechanisms (Kanno et al., 2006), which was described in detail earlier (Figure 6). Another notable candidate is *OsDHODH1* (*DIHYDROOROTATE DEHYDROGENASE 1*, Os02g0736400), whose role in promoting salt tolerance was described by Liu and coworkers (Liu et al., 2009) (see Figure S22 and Extended Results). In our study, *OsDHODH1* was identified as a high-confidence salinity-related gene in four traits, including high proline content (trait no. 41), which is related to osmoprotectants as it involves pyrimidine biosynthesis that generating proline precursors (Wang et al., 2003), and the gene was up-regulated in five days and three days after the treatments in rice and in Arabidopsis, respectively (Figures S22B-22E). In addition, the Arabidopsis loss-of-function mutant of the orthologous gene, *pvd1*, also exhibited restricted growth in terms of biomass and leaf areas under salt stress (Figure S22F).

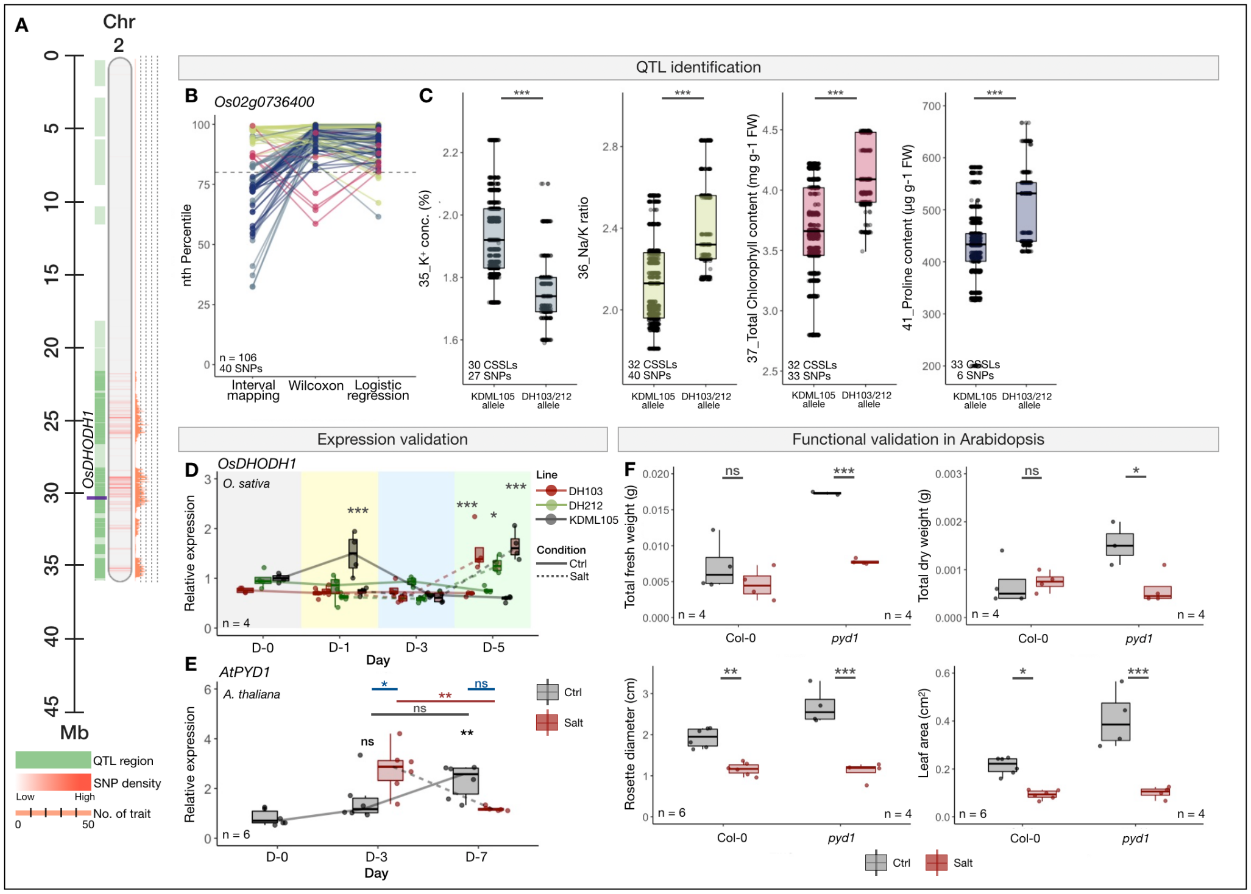

**Figure S22.** Experimental validations of *OsDHODH1* in rice (*O. sativa*) and its orthologue in Arabidopsis (*A. thaliana*). (A) Genomic location of *OsDHODH1* in the QTL region on Chromosome 2 of *O. sativa* (Nipponbare cv.). (B) PR-normalised confidence scores of each SNP position within the *OsDHODH1* gene obtained from the three QTL identification methods. Colours representing the four traits are as described in C. (C) Phenotype scores of CSSLs with the alleles from salt-susceptible KDML105, or salt-tolerant DH103/DH212 cultivars in four salt-responsive traits (trait no. 35, 36, 37 and 41). Phenotype scores were obtained from Pamuta et al. (2014). (D) Expression analysis of *OsDHODH1* in KDML105, DH103 and DH212 rice cultivars under the control and salt stress (100 mM NaCl) condition. The salt treatment was conducted using 16-day-old rice seedlings. (E) Expression analysis of *OsDHODH1* orthologous gene in Arabidopsis, *AtPYD1*, under the control and salt stress (100 mM NaCl) condition. The salt treatment was conducted using 10-day-old Col-0 Arabidopsis seedlings. (F) Morphological responses, namely total fresh weight, total dry weight, rosette diameter and leaf area of WT (Col-0) and loss-of-function mutant line (*pyd1*). The experiment was conducted using 7-day-old seedlings and the measurement was done 12 days in the control or salt stress (250 mM NaCl) conditions. Error bars represent standard deviations from four biological replicates. Asterisks (*) represent the significant p-value (one-way ANOVA in D and E and t-test in F) between the control and salt stress conditions.

*Signal transduction*

We have also identified multiple signal transduction genes as salinity-related candidates. For instance, we observed *OsSRWD1* (*SALT RESPONSIVE WD40 PROTEIN 1*, Os08g0497600) being characterised as a candidate in eight traits relating to salt-tolerant phenotypes, and it was up-regulated as early as one and three days after the salt stress treatments in rice and in Arabidopsis, respectively (Figures S23B-E). This falls in line with an earlier work by Huang and colleagues, which demonstrated that the WD40 protein subfamily promotes salt tolerance in the seedlings of IR64 rice under salt stress (Huang et al., 2008). Other candidates salinity-related genes with signal transduction functions that were identified in this study include *OsMSR2* (*MULTI-STRESS-RESPONSIVE GENE 2*, Os01g0955100), as previously characterised by Khrueasan et al. (2019) and Xu et al. (2013), protein kinases *OsCDPK7* (*CALCIUM-DEPENDENT PROTEIN KINASE 7*, Os04g0584750) (Saijo et al., 2000), *OsCPK12* (*CALCIUM-DEPENDENT PROTEIN KINASE* 12, Os04g0560600) (Asano et al., 2012) and *OsMKK4* (*MAP KINASE KINASE 4*, Os02g0787300) (Kumar et al., 2008). Note that a complete list of candidates with the functions relating to other biological processes such as reactive oxygen species (ROS) regulation, photosynthesis, and electron transportation, can be found in Table S12.

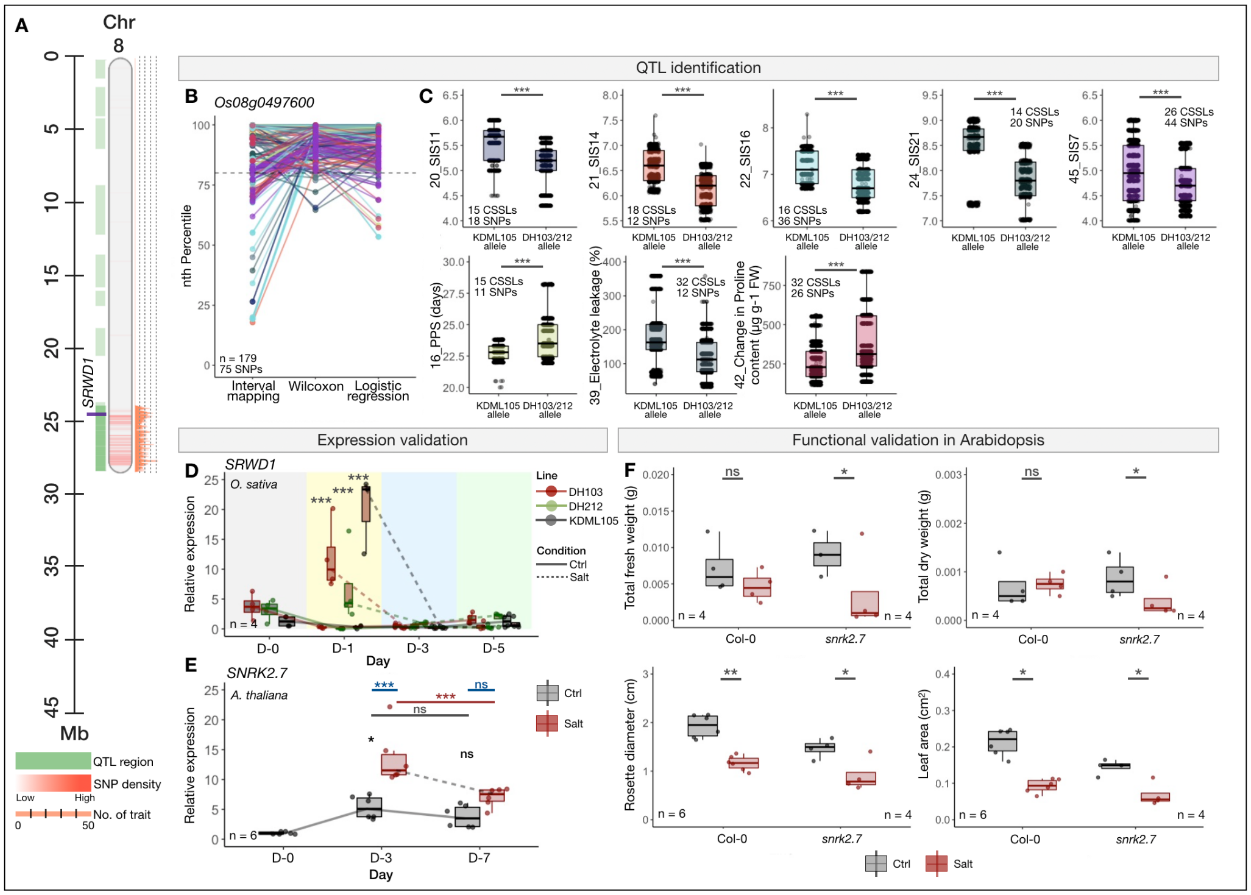


**Figure S23.** Experimental validations of *OsSRWD1* in rice (*O. sativa*) and its orthologue in Arabidopsis (*A. thaliana*). (A) Genomic location of *OsSRWD1* in the QTL region on Chromosome 8 of *O. sativa* (Nipponbare cv.). (B) PR-normalised confidence scores of each SNP position within the *OsSRWD1* gene obtained from the three QTL identification methods. Colours representing the four traits are as described in C. (C) Phenotype scores of CSSLs with the alleles from salt-susceptible KDML105, or salt-tolerant DH103/DH212 cultivars in 8 salt-responsive traits (trait no. 6, 20, 21, 22, 24, 39, 42 and 45). Phenotype scores were obtained from Kanjoo et al. (2011), Pamuta et al. (2014) and Nuanjan et al. (2016). (D) Expression analysis of *OsSRWD1* in KDML105, DH103 and DH212 rice cultivars under the control and salt stress (100 mM NaCl) condition. The salt treatment was conducted using 16-day-old rice seedlings. (E) Expression analysis of *OsSRWD1* orthologous gene in Arabidopsis, *AtSNRK2.7*, under the control and salt stress (100 mM NaCl) condition. The salt treatment was conducted using 10-day-old Col-0 Arabidopsis seedlings. (F) Morphological responses, namely total fresh weight, total dry weight, rosette diameter and leaf area of WT (Col-0) and loss-of-function mutant line (*snrk2.7*). The experiment was conducted using 7-day-old seedlings and the measurement was done 12 days in the control or salt stress (250 mM NaCl) conditions. Error bars represent standard deviations from four biological replicates. Asterisks (*) represent the significant p-value (one-way ANOVA in D and E and t-test in F) between the control and salt stress conditions.

*Uncharacterised function*

Another candidate gene was also being tested for its functional roles in salinity responses in this study is the hydrolase gene with the unknown function *Os02g0706900* (see Figure S25). In our investigation, *Os02g0706900* has been identified as a high-confidence salinity-related gene exhibiting significant associations with four traits, namely SIS (trait no. 5), K+ concentration (trait no. 35), Na/K ratio (trait no. 36), and Proline content (trait no. 41) (Figure S25C). Expression analysis revealed an up-regulation at Day 1 followed by down-regulation in rice (Figure S25D), a pattern was different from its Arabidopsis orthologous gene, *AT4G36610*, which demonstrated up-regulation after exposure to salt stress for 7 days (Figure S25E). Moreover, the phenotypic characteristics of the Arabidopsis mutant of the orthologous gene exhibited minimal changes, with only a reduction in leaf area observed under salt stress conditions (Figure S25F).

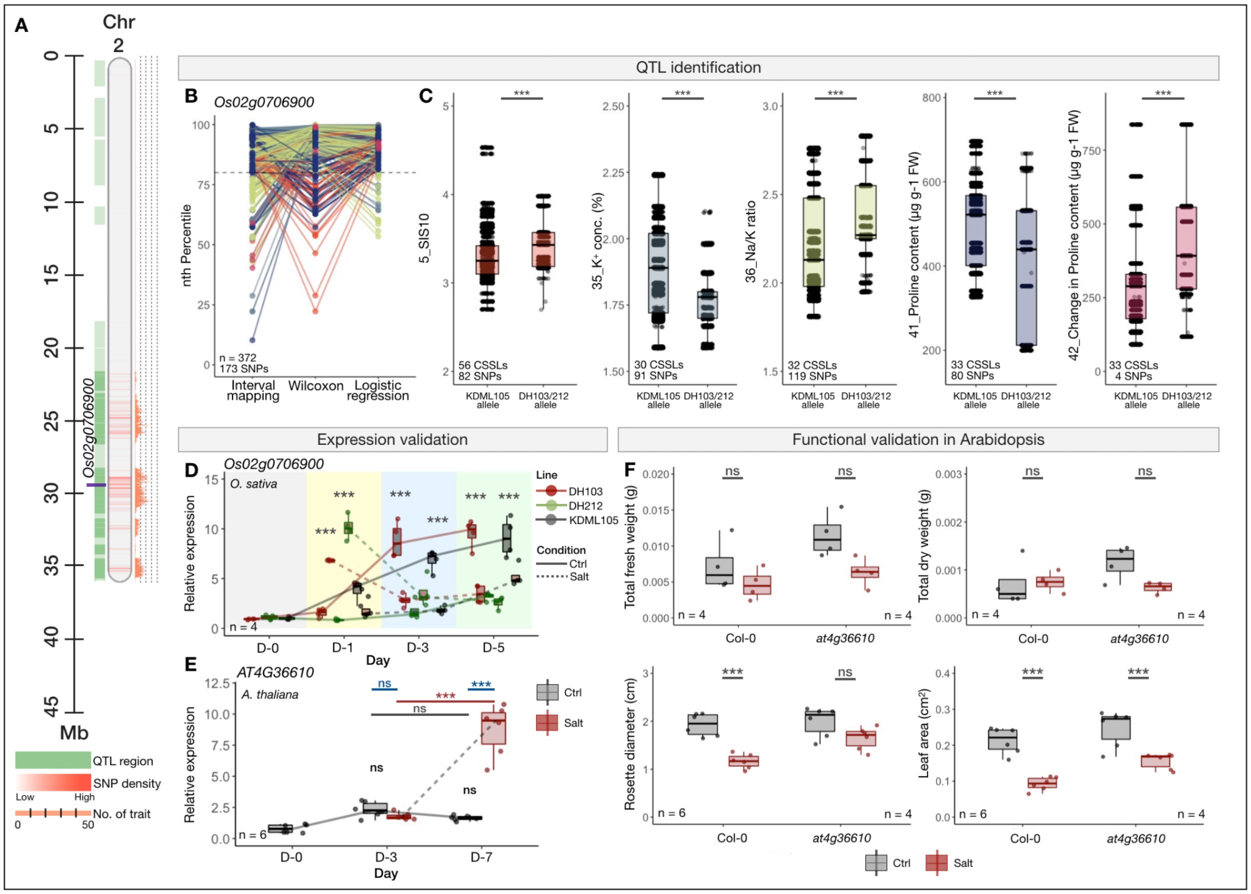


**Figure S25.** Experimental validations of *Os02g0706900* in rice (*O. sativa*) and its orthologue in Arabidopsis (*A. thaliana*). (A) Genomic location of *Os02g0706900* in the QTL region on Chromosome 2 of *O. sativa* (Nipponbare cv.). (B) PR-normalised confidence scores of each SNP position within the *Os02g0706900* gene obtained from the three QTL identification methods. Colours representing the five traits are as described in C. (C) Phenotype scores of CSSLs with the

alleles from salt-susceptible KDML105, or salt-tolerant DH103/DH212 cultivars in four salt-responsive traits (trait no. 5, 35, 36, 41 and 42). Phenotype scores were obtained from Kanjoo et al. (2011) and Pamuta et al. (2014). (D) Expression analysis of *Os02g0706900* in KDML105, DH103 and DH212 rice cultivars under the control and salt stress (100 mM NaCl) condition. The salt treatment was conducted using 16-day-old rice seedlings. (E) Expression analysis of *Os02g0706900* orthologous gene in Arabidopsis, *AT4G36610*, under the control and salt stress (100 mM NaCl) condition. The salt treatment was conducted using 10-day-old Col-0 Arabidopsis seedlings. (F) Morphological responses, namely total fresh weight, total dry weight, rosette diameter and leaf area of WT (Col-0) and loss-of-function mutant line (*At4g36610*). The experiment was conducted using 7-day-old seedlings and the measurement was done 12 days in the control or salt stress (250 mM NaCl) conditions. Error bars represent standard deviations from four biological replicates. Asterisks (*) represent the significant p-value (one-way ANOVA in D and E and t-test in F) between the control and salt stress conditions.

**References**


Campo, S. and San Segundo, B. (2020). Systemic induction of phosphatidylinositol-based signaling in leaves of arbuscular mycorrhizal rice plants. *Sci. Rep.* 10: 15896. doi: 10.1038/s41598-020-72985-6

Chutimanukul, P., Kositsup, B., Plaimas, K., Buaboocha, T., Siangliw, M., Toojinda, T., et al. (2018b). Photosynthetic responses and identification of salt tolerance genes in a Chromosome Segment Substitution Line of 'Khao Dawk Mali 105' Rice. *Environ. Exp. Bot.* 155, 497-508. doi: 10.1016/j.envexpbot.2018.07.019

Cosgrove, D. J. (2000). Loosening of plant cell walls by expansins. *Nature* 407**,** 321-326. doi: 10.1038/35030000

Fukuda, A., Nakamura, A., Tagiri, A., Tanaka, H., Miyao, A., Hirochika, H., et al. (2004). Function, intracellular localization and the importance in salt tolerance of a vacuolar Na(+)/H(+) antiporter from rice. *Plant Cell Physiol.* 45**,** 146-159. doi: 10.1093/pcp/pch014

Ganie, S. A., Molla, K. A., Henry, R. J., Bhat, K. V. and Mondal, T. K. (2019). Advances in understanding salt tolerance in rice. *Theor. Appl. Genet.* 132**,** 851-870. doi: 10.1007/s00122-019-03301-8

Hamed, K. B., Ellouzi, H., Talbi, O. Z., Hessini, K., Slama, I., Ghnaya, T., Bosch, S. M., Savour, A. and Abdelly, C. (2013). Physiological response of halophytes to multiple stresses. *Funct. Plant Biol.* 40: 883-896. doi: 10.1071/FP13074

Huang, J., Wang, M. M., Bao, Y. M., Sun, S. J., Pan, L. J. and Zhang, H. S. (2008). SRWD: a novel WD40 protein subfamily regulated by salt stress in rice (*Oryza sativa* L.). *Gene* 424**,** 71-79. doi: 10.1016/j.gene.2008.07.027

Huda, K. M., Banu, M. S., Garg, B., Tula, S., Tuteja, R. and Tuteja, N. (2013). OsACA6, a P-type IIB $Ca^{2+}$ ATPase promotes salinity and drought stress tolerance in tobacco by ROS scavenging and enhancing the expression of stress-responsive genes. *Plant J.* 76**,** 997-1015. doi: 0.1111/tpj.12352

Islam, M. O., Kato, H., Shima, S., Tezuka, D., Matsui, H. and Imai, R. (2019). Functional identification of a rice trehalase gene involved in salt stress tolerance. *Gene* 685**,** 42-49. doi: 10.1016/j.gene.2018.10.071

Ito, Y. and Kurata, N. (2006). Identification and characterization of cytokinin-signalling gene families in rice. *Gene* 382**,** 57-65. doi: 10.1016/j.gene.2006.06.020

Joo, J., Lee, Y. H., Kim, Y. K., Nahm, B. H. and Song, S. I. (2013). Abiotic stress responsive rice *ASR1* and *ASR3* exhibit different tissue-dependent sugar and hormone-sensitivities. *Mol. Cells* 35: 421-435. doi: 10.1007/s10059-013-0036-7

Kanjoo, V., Jearakongman, S., Punyawaew, K., Siangliw, J., Siangliw, M., Vanavichit, A., et al. (2011). Co-location of quantitative trait loci for drought and salinity tolerance in rice. *Thai J. Genet.* 4, 126-138. doi: 10.14456/tjg.2011.3

Kanno, Y., Otomo, K., Kenmoku, H., Mitsuhashi, W., Yamane, H., Oikawa, H., et al. (2006). Characterization of a rice gene family encoding type-A diterpene cyclases. *Biosci. Biotechnol. Biochem.* 70**,** 1702-1710. doi: 10.1271/bbb.60044

Khrueasan, N., Chutimanukul, P., Plaimas, K., Buaboocha, T., Siangliw, M., Toojinda, T., et al. (2019). Comparison between the transcriptomes of 'KDML105' rice and a salt-tolerant chromosome segment substitution line. *Genes* 10, 742. doi: 10.3390/genes10100742

Khrueasan, N., Plaimas, K., Kositsup, B., Chaidee, A., Buaboocha, T., Siangliw, M., et al. (2013). Gene co-expression network of predicted salt tolerance region on chromosome 1 in rice (*Oryza sativa* L.). *Thai J. Genet.* 6**,** 30-35.

Khrueasan, N., Siangliw, M., Toojinda, T., Imyim, A., Buaboocha, T. and Chadchawan, S. (2020). Physiological mechanisms of the seedling stage salt tolerance of near isogenic rice lines with the 'KDML105' genetic background. *Int. J. Agric. Biol.* 23**,** 927-934. doi: 10.17957/IJAB/15.1371

Knight, H., and Knight, M. R. (2001). Abiotic stress signalling pathways: specificity and cross-talk. *Trends Plant Sci.* 6: 262-267. doi: 10.1016/s1360-1385(01)01946-x

Kumar, S. and Trivedi, P. K. (2018). A vacuolar membrane ferric-chelate reductase, OsFRO1, alleviates Fe toxicity in rice (*Oryza sativa* L.). *Front. Plant Sci.* 10, 700. doi: 10.3389/fpls.2019.00700

Liu, W. Y., Wang, M. M., Huang, J., Tang, H. J., Lan, H. X. and Zhang, H. S. (2009). The *OsDHODH1* gene is involved in salt and drought tolerance in rice. *J. Integr. Plant Biol.* 51**,** 825-33. doi: 10.1111/j.1744-7909.2009.00853.x

Moore, K. L., Chen, Y., van de Meene, A. M. L., Hughes, L., Liu, W., Geraki, T., et al. (2014). Combined NanoSIMS and synchrotron X-ray fluorescence reveal distinct cellular and subcellular distribution patterns of trace elements in rice tissues. *New Phytol.* 201**,** 104-115. doi: 10.1111/nph.12497

Muhammad, I., Jing, X.-Q., Shalmani, A., Ali, M., Yi, S., Gan, P.-F., et al. (2018). Comparative in silico analysis of ferric reduction oxidase (FRO) genes expression patterns in response to abiotic stresses, metal and hormone applications. *Molecules* 23, 1163. doi: 10.3390/molecules23051163

Munns, R. and Tester, M. (2008). Mechanisms of salinity tolerance. *Annu. Rev. Plant Biol.* 59**,** 651-681. doi: 10.1146/annurev.arplant.59.032607.092911

Ngo, A. H., Kanehara, K. and Nakamura, Y. (2019). Non-specific phospholipases C, NPC2 and NPC6, are required for root growth in Arabidopsis. *The Plant J.* 100, 825-835. doi: 10.1111/tpj.14494

Nounjan, N., Siangliw, J. L., Toojinda, T., Chadchawan, S. and Theerakulpisut, P. (2016). Salt-responsive mechanisms in chromosome segment substitution lines of rice (*Oryza sativa* L. cv. KDML105). *Plant Physiol. Biochem.* 103**,** 96-105. doi: 10.1016/j.plaphy.2016.02.038

Silveira, J. A., Viégas, R.deA., da Rocha, I. M., Moreira, A. C., Moreira, R.deA. and Oliveira, J. T. (2003). Proline accumulation and glutamine synthetase activity are increased by salt-induced proteolysis in cashew leaves. *J. Plant Physiol.* 160, 115-123. doi: 10.1078/0176-1617-00890

Singh, P., Choudhary, K. K., Chaudhary, N., Gupta, S., Sahu, M., Tejaswini, B., et al. (2022). Salt stress resilience in plants mediated through osmolyte accumulation and its crosstalk mechanism with phytohormones. *Front. Plant Sci.* 13, 1006617. doi: 10.3389/fpls.2022.1006617

Soda, N., Sharan, A., Gupta, B. K., Singla-Pareek, S. L. and Pareek, A. (2016). Evidence for nuclear interaction of a cytoskeleton protein (OsIFL) with metallothionein and its role in salinity stress tolerance. *Sci. Rep.* 6**,** 34762. doi: 10.1038/srep34762

Sripinyowanich, S., Chamnanmanoontham, N., Udomchalothorn, T., Maneeprasopsuk, S., Santawee, P., Buaboocha, T., et al. (2013). Overexpression of a partial fragment of the salt-responsive gene OsNUC1 enhances salt adaptation in transgenic *Arabidopsis thaliana* and rice (*Oryza sativa* L.) during salt stress. *Plant Sci.* 213**,** 67-78. doi: 10.1016/j.plantsci.2013.08.013

Wang, G.-F., Li, W.-Q., Li, W.-Y., Wu, G.-L., Zhou, C.-Y. and Chen, K.-M. (2013). Characterization of rice NADPH oxidase genes and their expression under various environmental conditions. *Int. J. Mol. Sci.* 14, 9440–9458. doi: 10.3390/ijms14059440

Wang, J., Nan, N., Li, N., Liu, Y., Wang, T.-J., Hwang, I., et al. (2020). A DNA methylation reader–chaperone regulator–transcription factor complex activates *OsHKT1;5* expression during salinity stress. *The Plant Cell* 32**,** 3535-3558. doi: 10.1105/tpc.20.00301

Wang, W., Vinocur, B. & Altman, A. (2003). Plant responses to drought, salinity and extreme temperatures: towards genetic engineering for stress tolerance. *Planta* 218, 1–14. doi: 10.1007/s00425-003-1105-5

Wu, H. (2018). Plant salt tolerance and $Na^+$ sensing and transport. *Crop J.* 6**,** 215-225. doi: 10.1016/j.cj.2018.01.003
