# Supplementary material for: Comparative quantitative trait loci analysis framework reveals relationships between salt stress responsive phenotypes and pathways
Source: Front Plant Sci. 2024 Feb 23;15:1264909. doi: 10.3389/fpls.2024.1264909 (PMC10920293; doi:10.3389/fpls.2024.1264909)
Supplement: Supplementary file 1 [file DataSheet_1.zip › Supplementary Figures.pdf]

Supplementary Material Figures

Comparative Quantitative Trait Loci Analysis Framework Reveals Relationships between Salt Stress Responsive Phenotypes and Pathways

Sunadda Phosuwan[1,2], Noppawan Nounjan[3], Piyada Theerakulpisut[4], Meechai Siangliw[5,*] and Varodom Charoensawan[2,6,7,8,9,10*]

[1]Doctor of Philosophy Program in Biochemistry (International Program), Faculty of Science, Mahidol University, Bangkok, Thailand

[2]Department of Biochemistry, Faculty of Science, Mahidol University, Bangkok, Thailand

[3]Biodiversity and Environmental Management Division, International College, Khon Kaen University, Khon Kaen, Thailand

[4]Salt-tolerant Rice Research Group, Department of Biology, Faculty of Science, Khon Kaen University, Khon Kaen, Thailand

[5]National Center for Genetic Engineering and Biotechnology (BIOTEC), Pathum Thani, Thailand.

[6]Integrative Computational BioScience (ICBS) center, Mahidol University, Nakhon Pathom, Thailand

[7]Division of Medical Bioinformatics, Research Department, Faculty of Medicine Siriraj Hospital, Mahidol University, Bangkok, Thailand

[8]Department of Biochemistry, Faculty of Medicine Siriraj Hospital, Mahidol University, Bangkok, Thailand

[9]Siriraj Genomics, Faculty of Medicine Siriraj Hospital, Mahidol University, Bangkok, Thailand

[10]School of Chemistry, Institute of Science, Suranaree University of Technology, Nakhon Ratchasima, Thailand

*** Correspondence:**
Varodom Charoensawan and Meechai Siangliw
varodom.cha@mahidol.ac.th, meechai@biotec.or.th

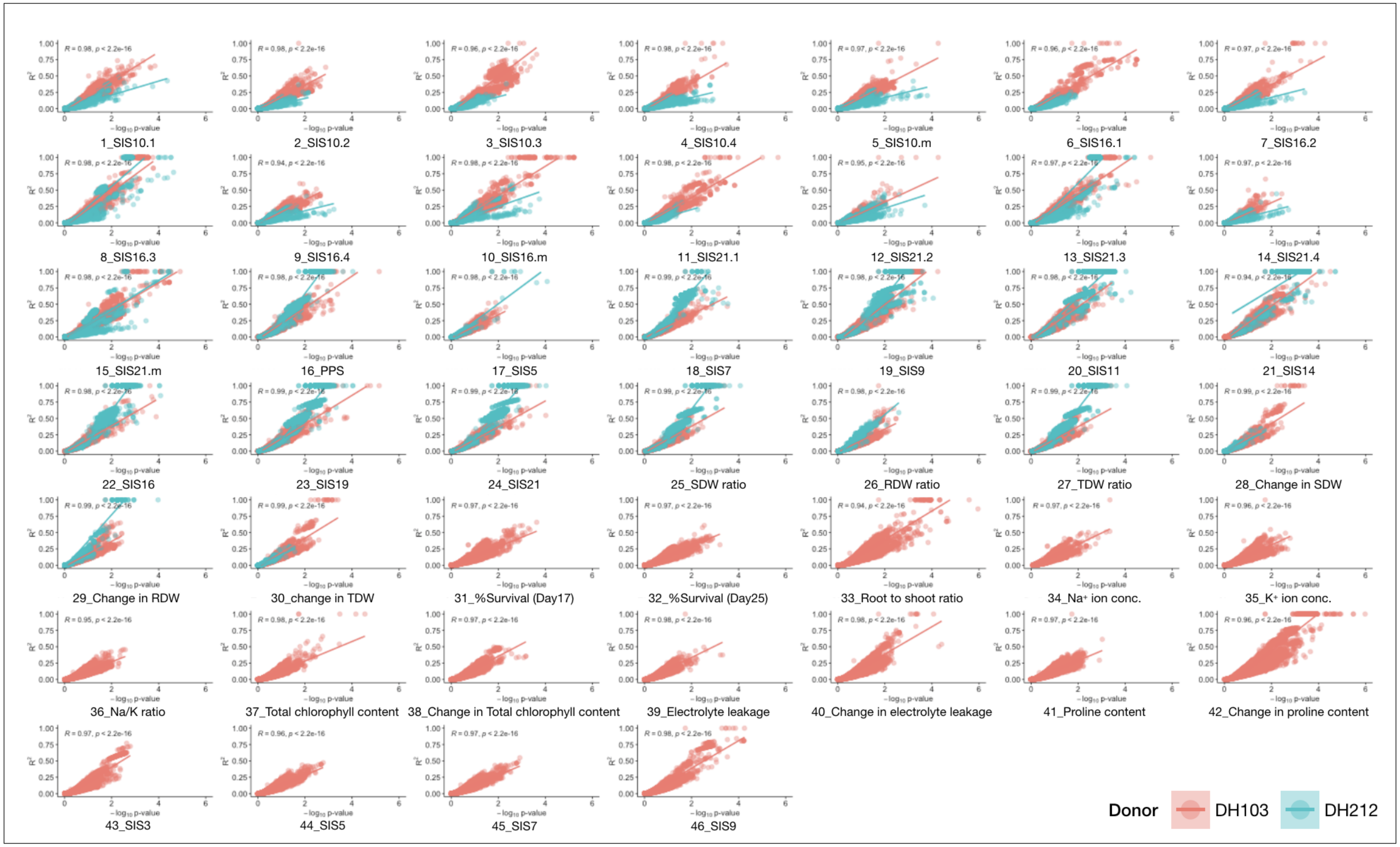

Figure S1. Scatter plots between -log$_{10}$(p-value) and coefficient of determination (R^2) of 46 salt-responsive traits. Spearman correlation coefficients were shown on the top-left panel of each plot. The pink and turquoise lines represent the linear regression, based on the DH103 and DH212-derived CSSL populations, respectively.

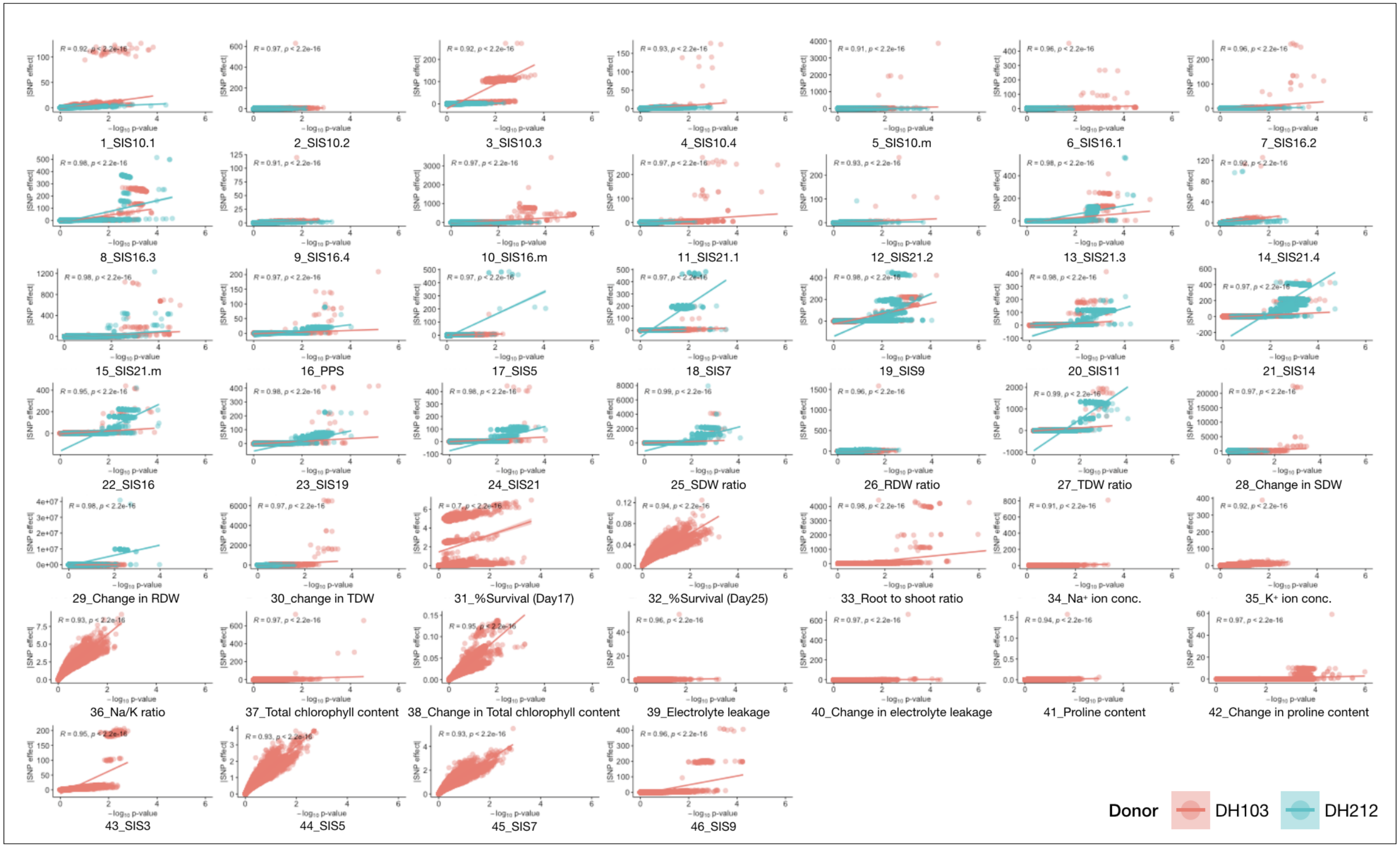

Figure S2. Scatter plots between -$\log_{10}$(p-value) and absolute values of SNP effect (coefficient: |SNP effect|) of 46 salt-responsive traits. Spearman correlation coefficients were shown on the top-left panel of each plot. The pink and turquoise lines represent the linear regression, based on the DH103- and DH212-derived CSSL populations, respectively.

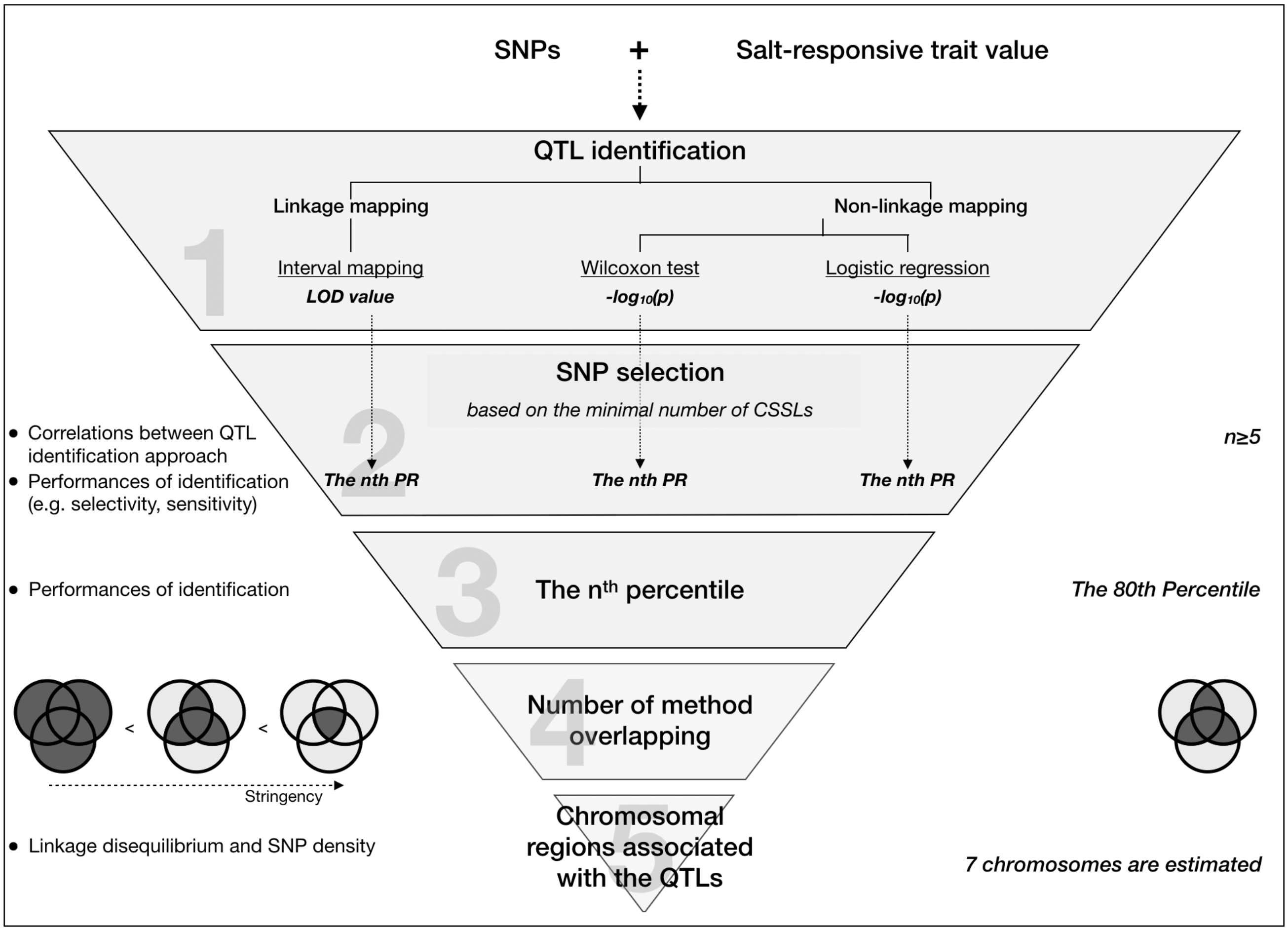

Figure S3. Salinity-related gene identification framework applied to the 46 salt-responsive traits of interest from three different QTL studies.

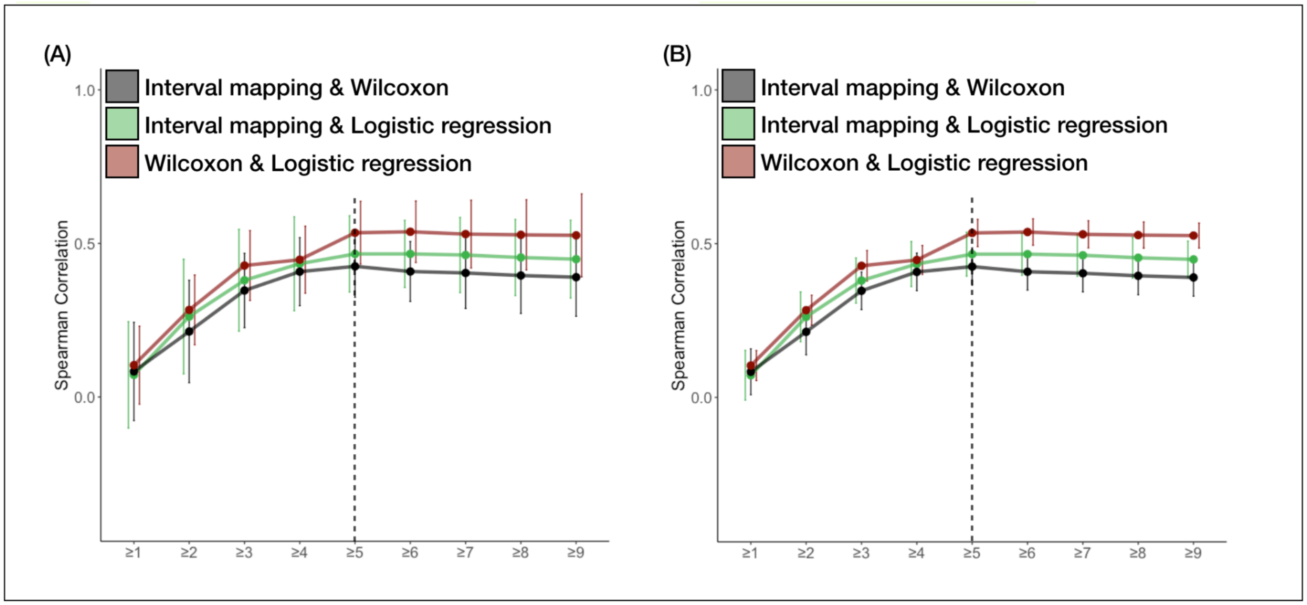

Figure S4. Spearman correlation coefficients among 46 salt-responsive traits between QTL confident scores of two QTL identification methods when the SNPs that were presented in more than ‘n’ CSSLs. The error bars represent the (A) standard deviations (SDs), and (B) confidence intervals (CIs) among different salt-responsive traits. The dashed line represents the appropriate minimal number of CSSLs.

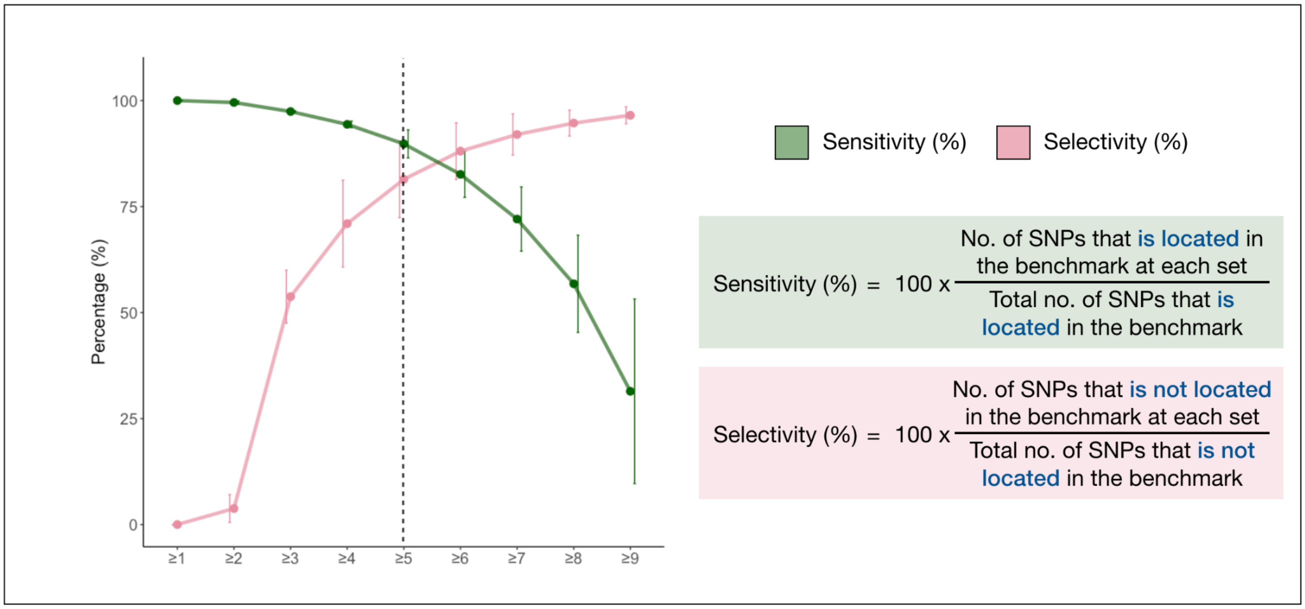

Figure S5. The percentage of selectivity and sensitivity of SNPs that were presented in minimum ‘n’ CSSLs. The error bars represent standard deviations (SDs) among the salt-responsive traits. The dashed line represents the appropriate minimal number of CSSLs.

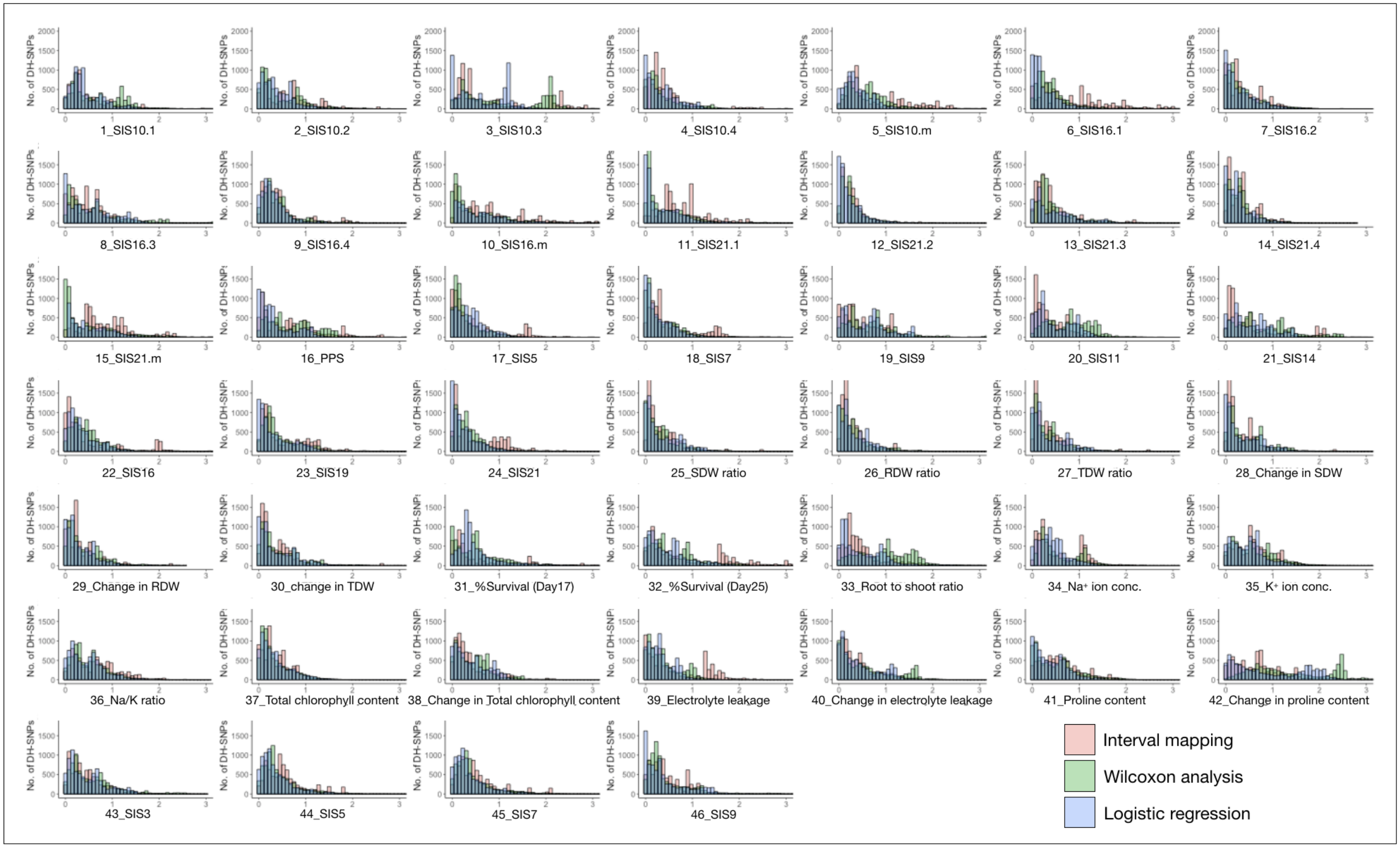

Figure S6. Distribution of LOD, -log$_{10}$(p) and -log$_{10}$(p) values, obtained from Interval mapping (pink), Wilcoxon signed-rank test (green) and Logistic regression analysis (blue), respectively, of the 46 salt-responsive traits in this study.

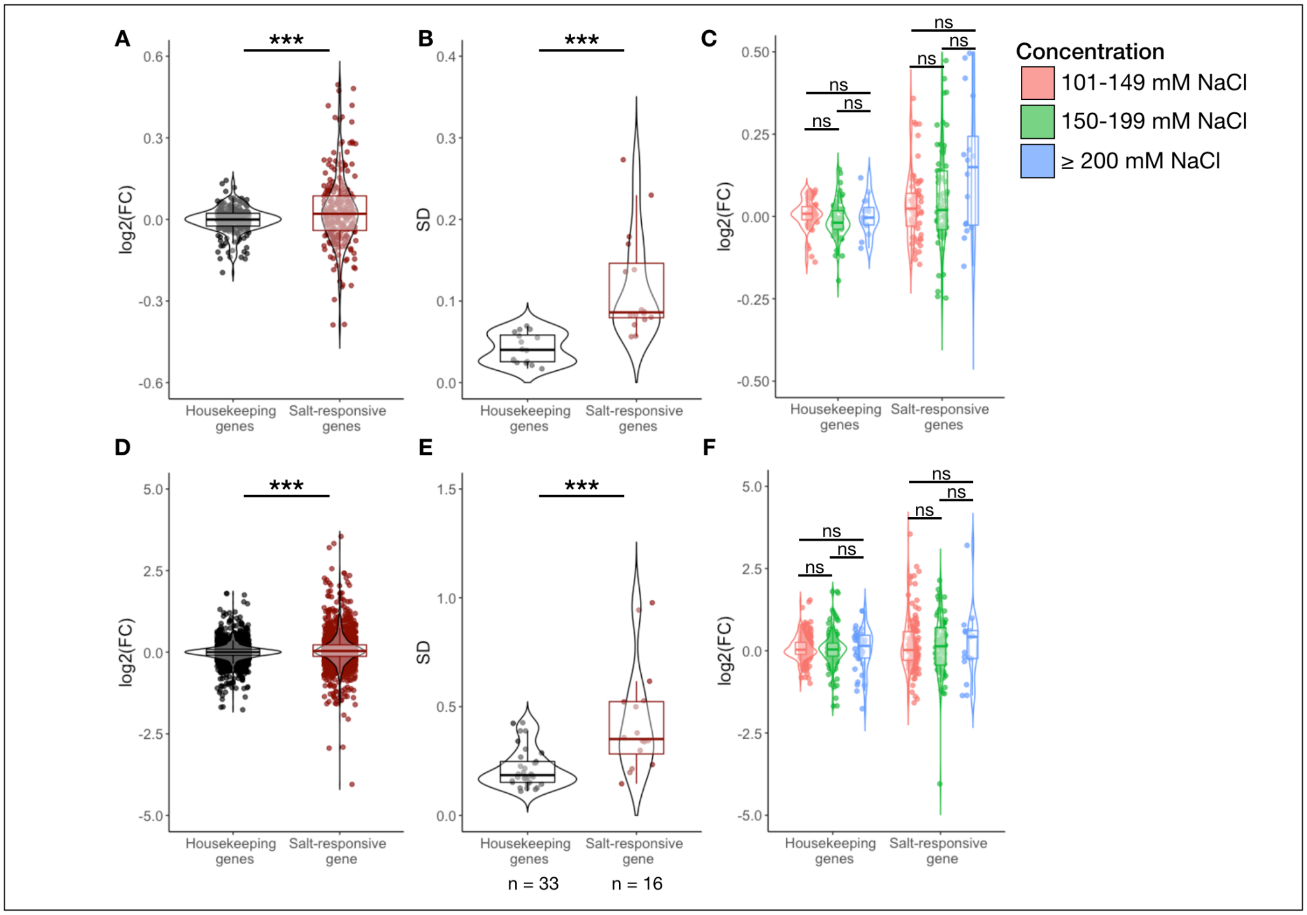

Figure S7. Transcriptomes obtained from publicly available (Top) microarray and (Bottom) RNA-seq datasets. (A and C) Distribution of expression values (Log_2FC : Log_2(Salt/Ctrl)) and (B and D) standard deviations (SDs) of the housekeeping and salt-responsive genes. (D and F) Distribution of expression values at different salt concentrations. Lists of the housekeeping and salt-responsive genes were obtained from Narsai et al. (2010) and Mollar et al. (2015), respectively. (***, p<0.001, unpaired *t*-test)

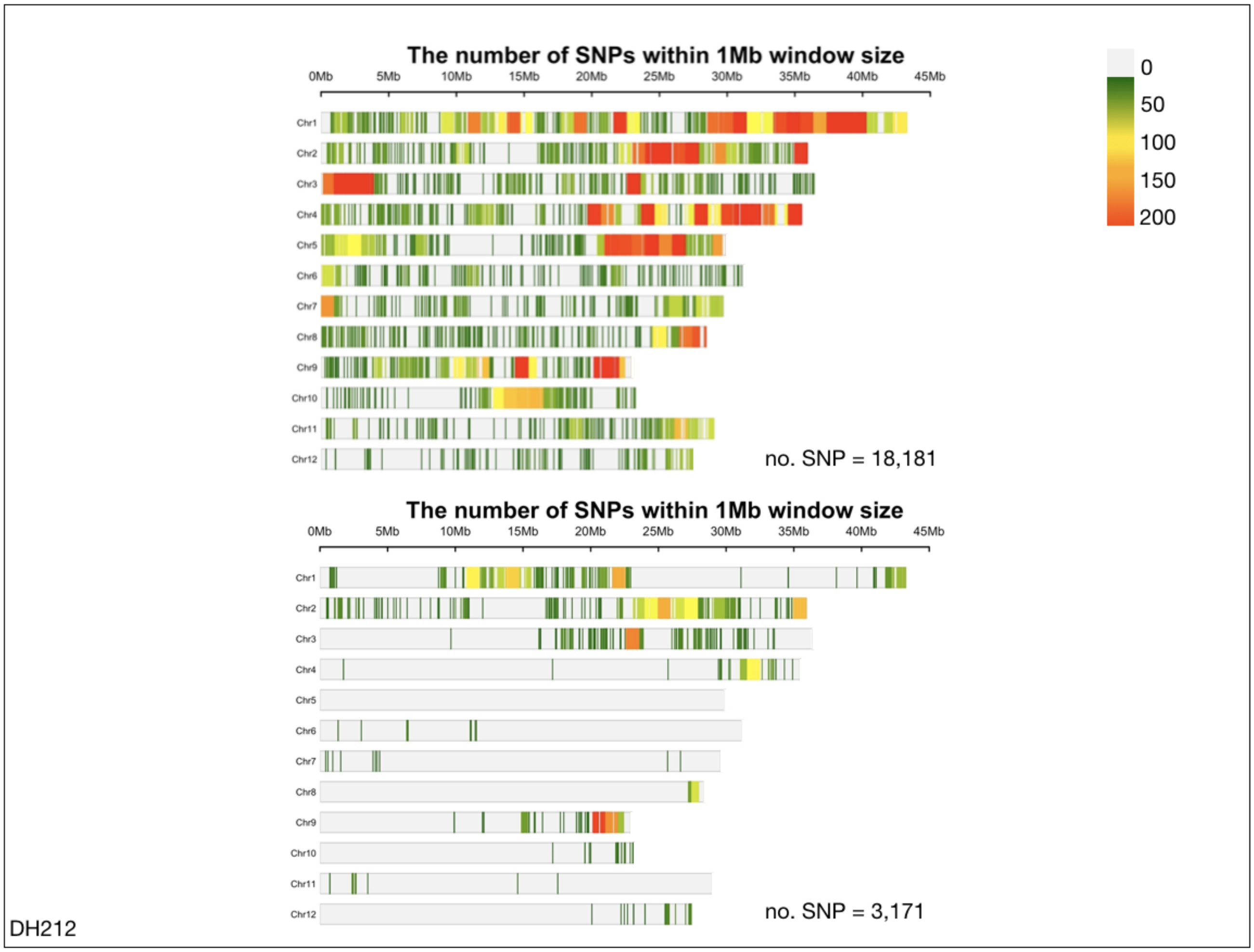

Figure S8. Total SNPs identified from DH212 (Top). Informative SNPs between the chromosome of the 'DH212'-derived rice CSSLs and the recurrent parent 'KDML105' (Bottom). Colour lines represent the numbers of SNPs within 1 Mb window size.

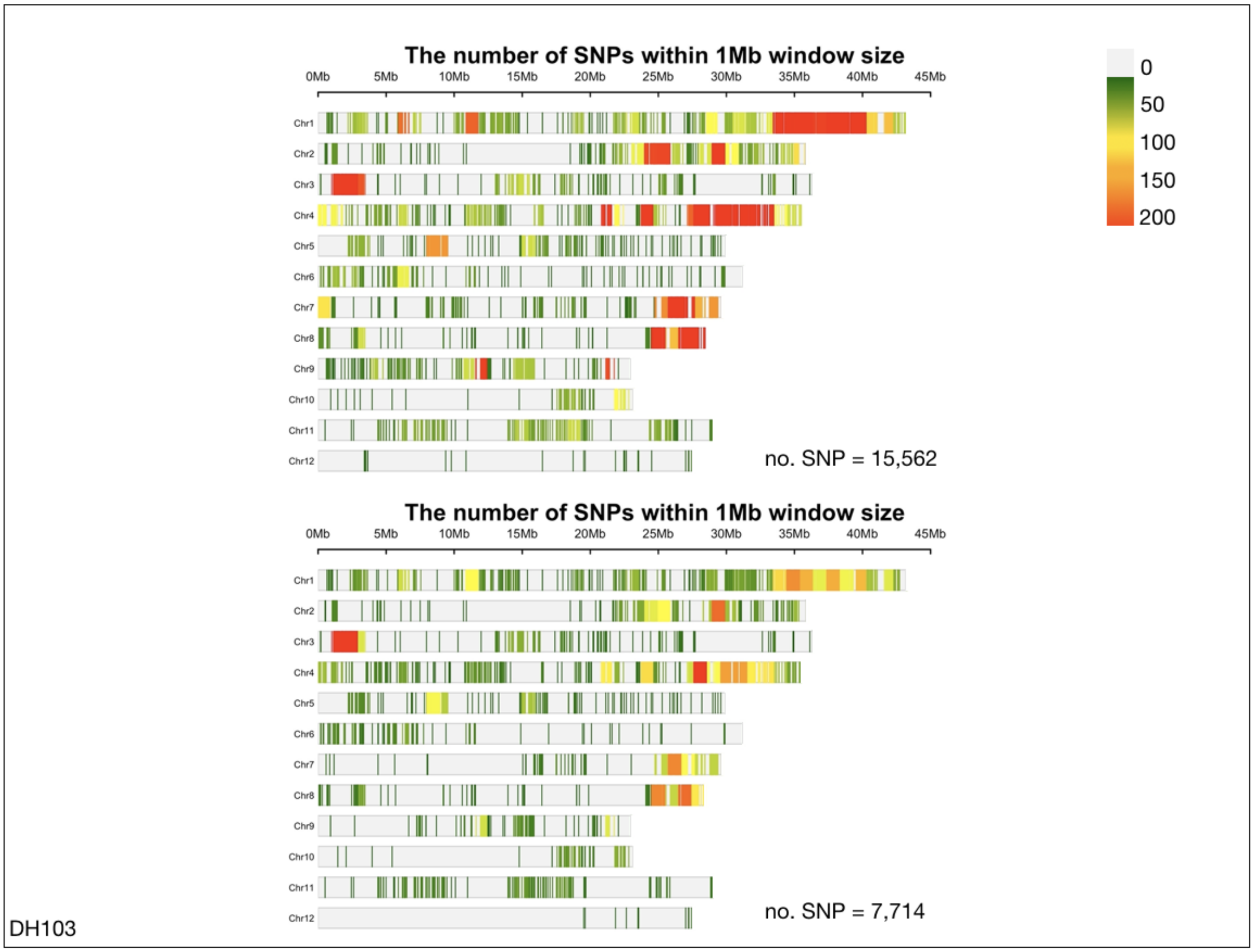

Figure S9. Total SNPs identified from DH103 (Top). Informative SNPs between the chromosome of the 'DH212'-derived rice CSSLs and the recurrent parent 'KDML105' (Bottom). Colour lines represent the numbers of SNPs within 1 Mb window size.

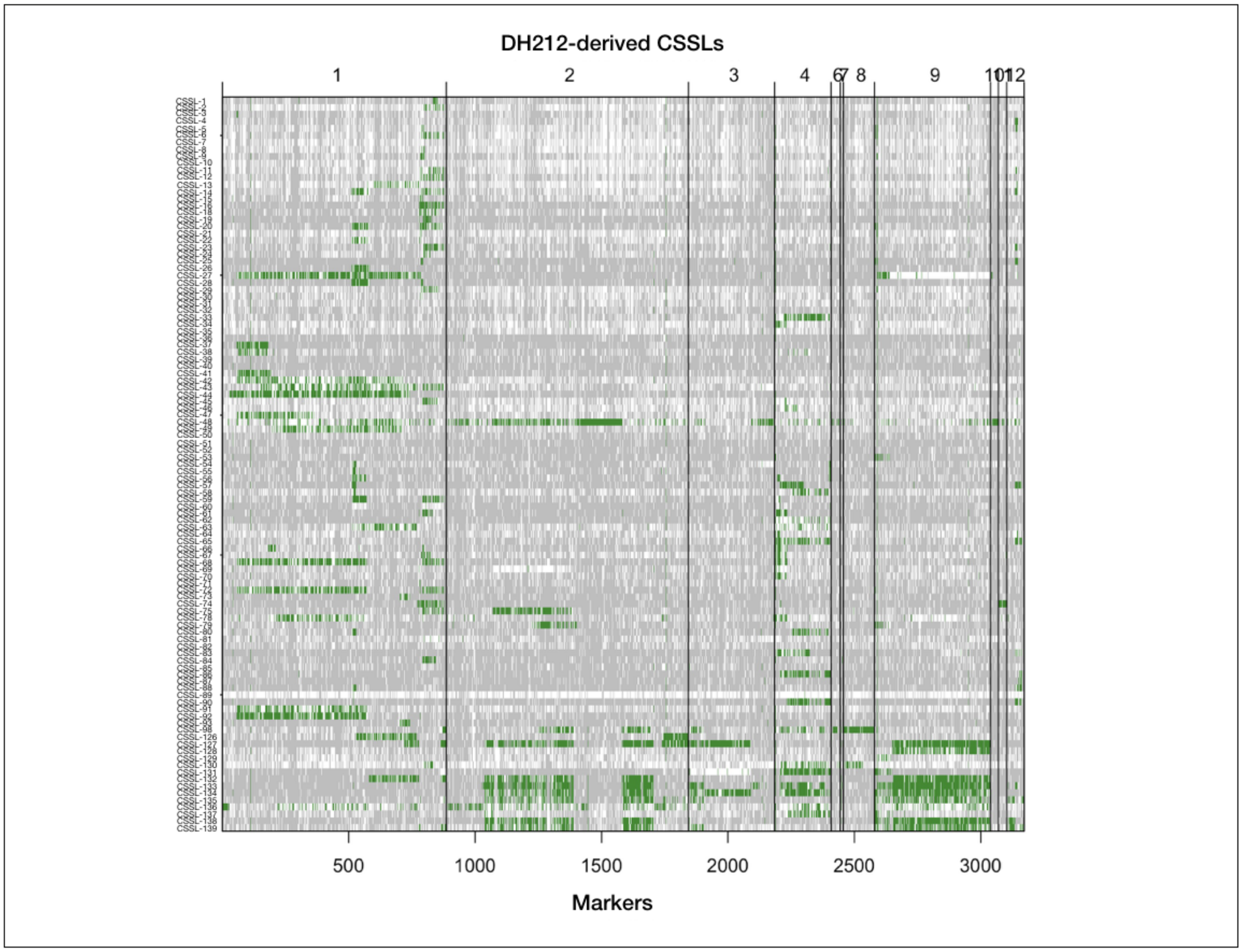

Figure S10. Genomic locations of the SNPs detected in the 104 DH212-derived CSSLs.

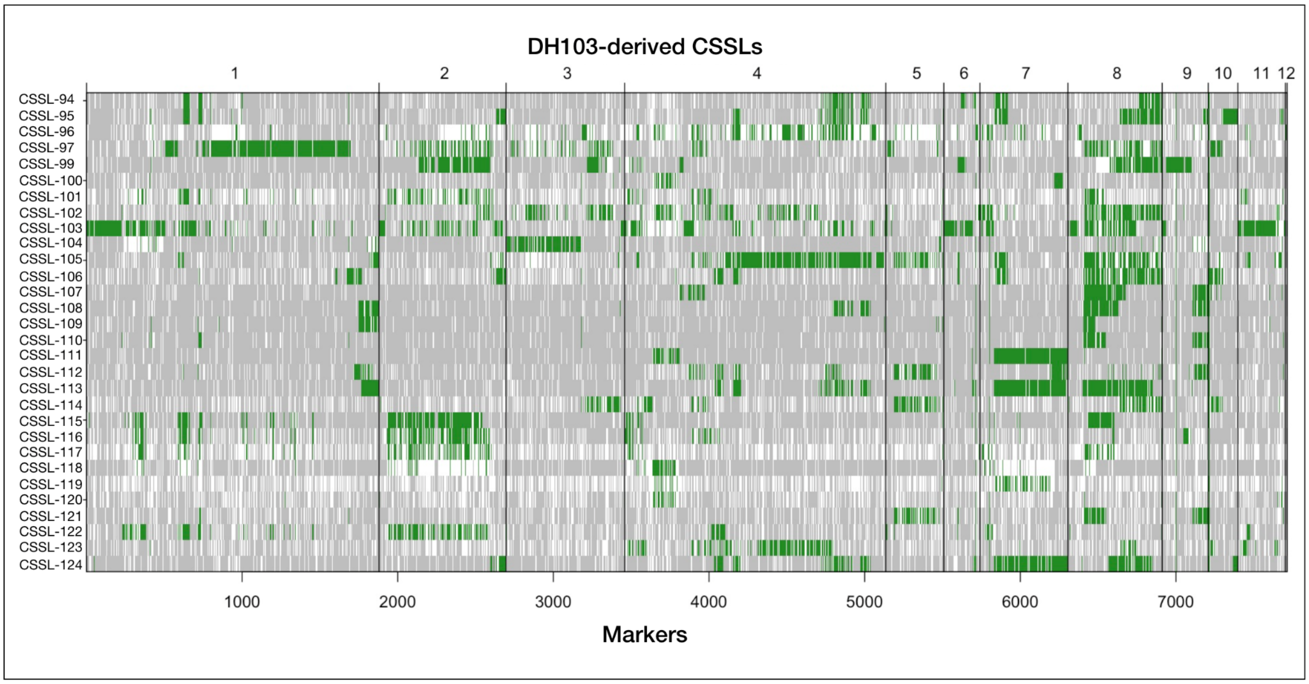

Figure S11. Genomic locations of the SNPs detected in the 31 DH103-derived CSSLs.

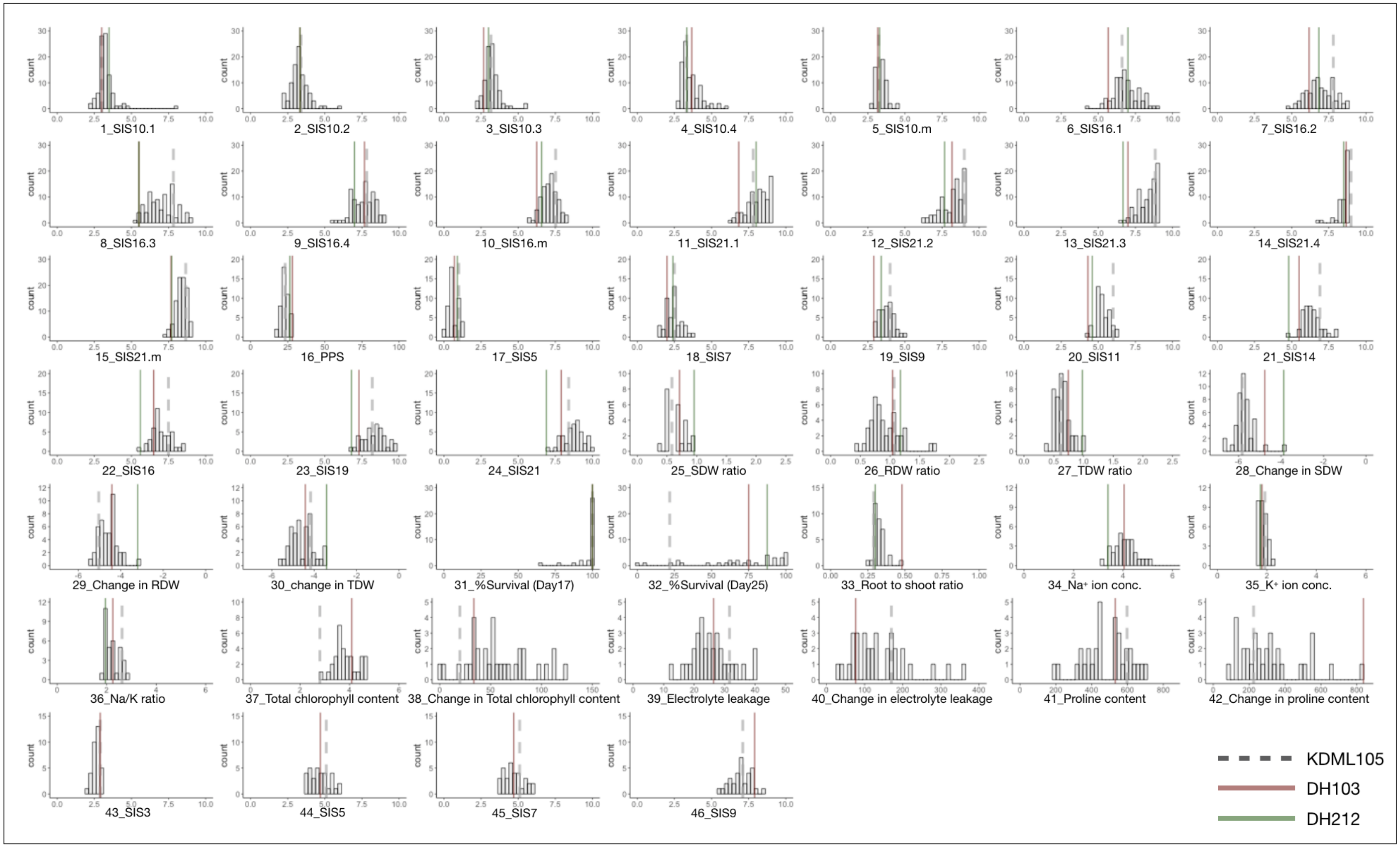

Figure S12. Forty-six (46) salt-responsive traits of CSSLs and their parents (DH103, DH212 and KDML105) (see Tables 1 and S1). Red, green and dashed-grey lines represent phenotype and trait properties of DH103, DH212 and KDML105, respectively.

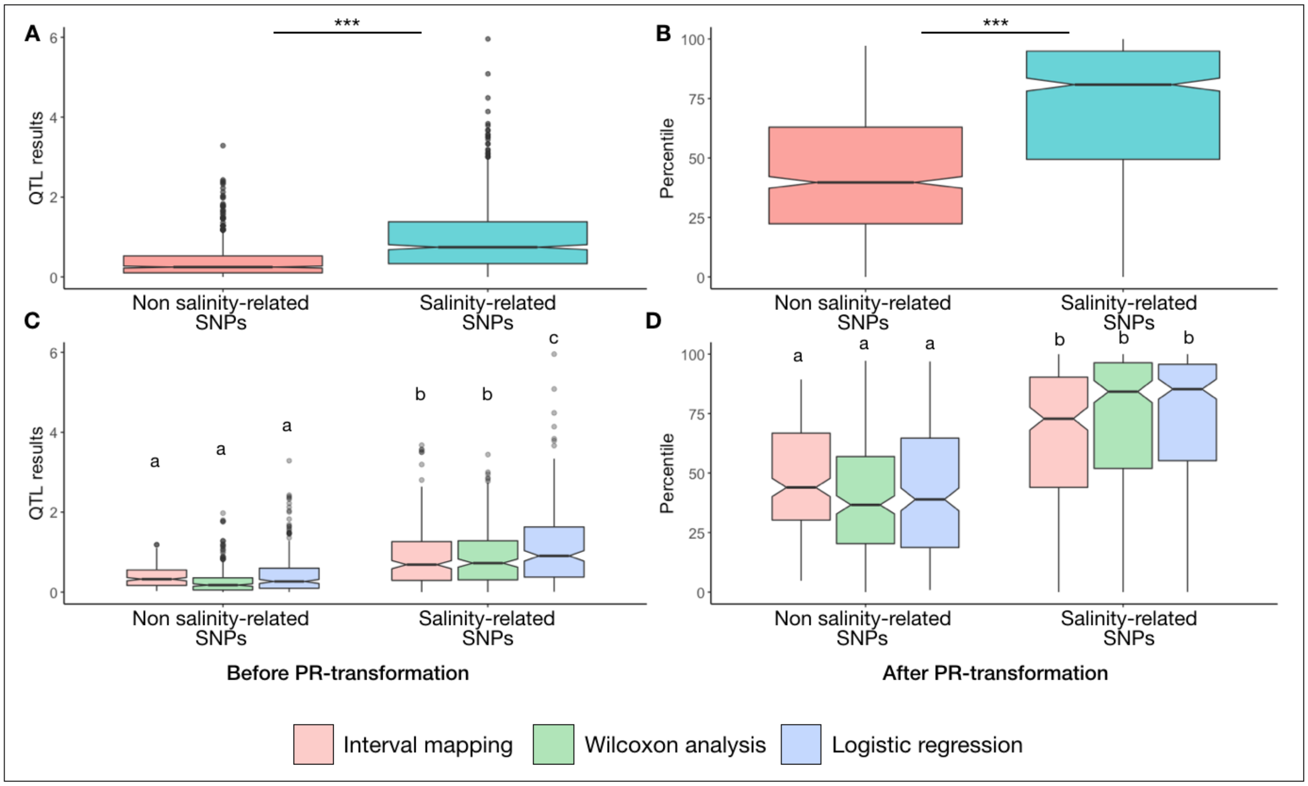

Figure S13. The QTL confident values of 46 salt-responsive traits, obtained from the SNPs locating (salinity-related SNPs) and not locating (non-salinity-related SNPs) in the salinity-responsive QTLs according to the benchmark (Table S2) before (A and C) and after transformed (B and D) to the percentile rank values. Kruskal-Wallis and Tukey's test ($p<0.05$) were performed between QTL confidence values locating and non-locating in the salt-responsive QTLs (A and B) and among QTL confidence values obtained from different methods (C and D), respectively.

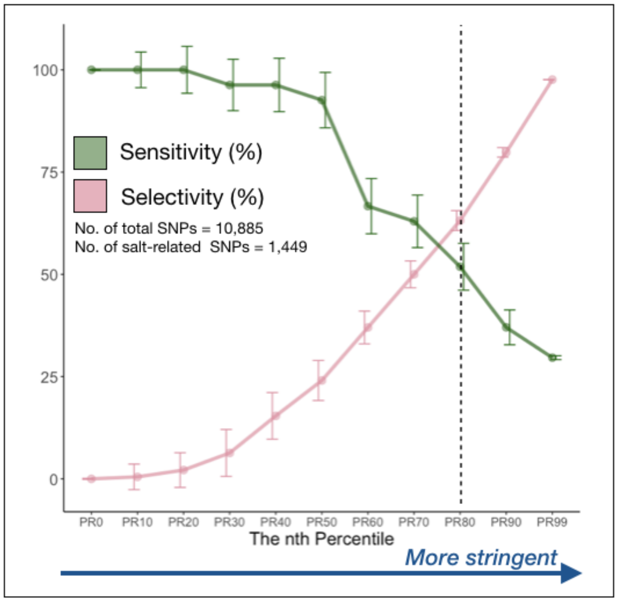

Figure S14. Selectivity and sensitivity (%) of SNPs when the QTL confident values of SNP_i were higher than the 'n'-th percentile (zero-th (0) to one-hundred-th (100)) in at least one QTL identification method. The error bars represent standard deviations (SDs). values among the salt-responsive traits. The dashed line represents the appropriate minimal percentile rank for the cut-off.

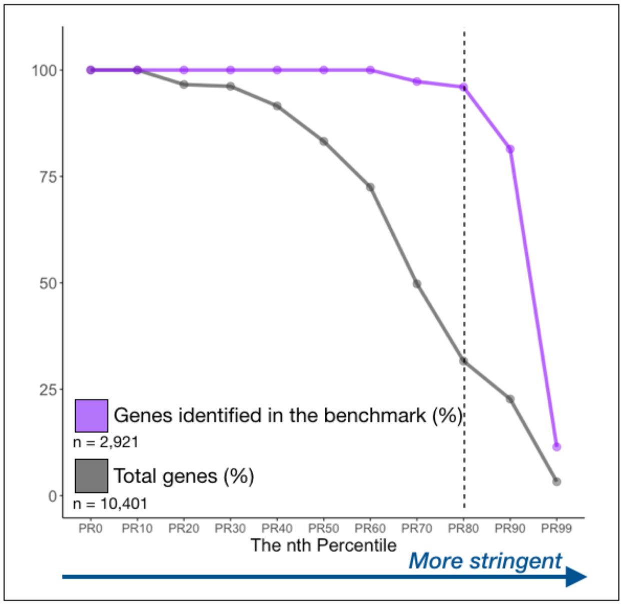

Figure S15. Number of total genes and genes located in salinity-related QTL regions (%) based on the benchmark (Table S2) when the QTL confident values of SNP_i located in the certain genes were higher than the 'n'-th percentile (zero-th (0) to one-hundred-th (100)) in at least one QTL identification method. The error bars represent standard deviations (SDs). values among the salt-responsive traits. The dashed line represents the appropriate minimal percentile rank for the cut-off.

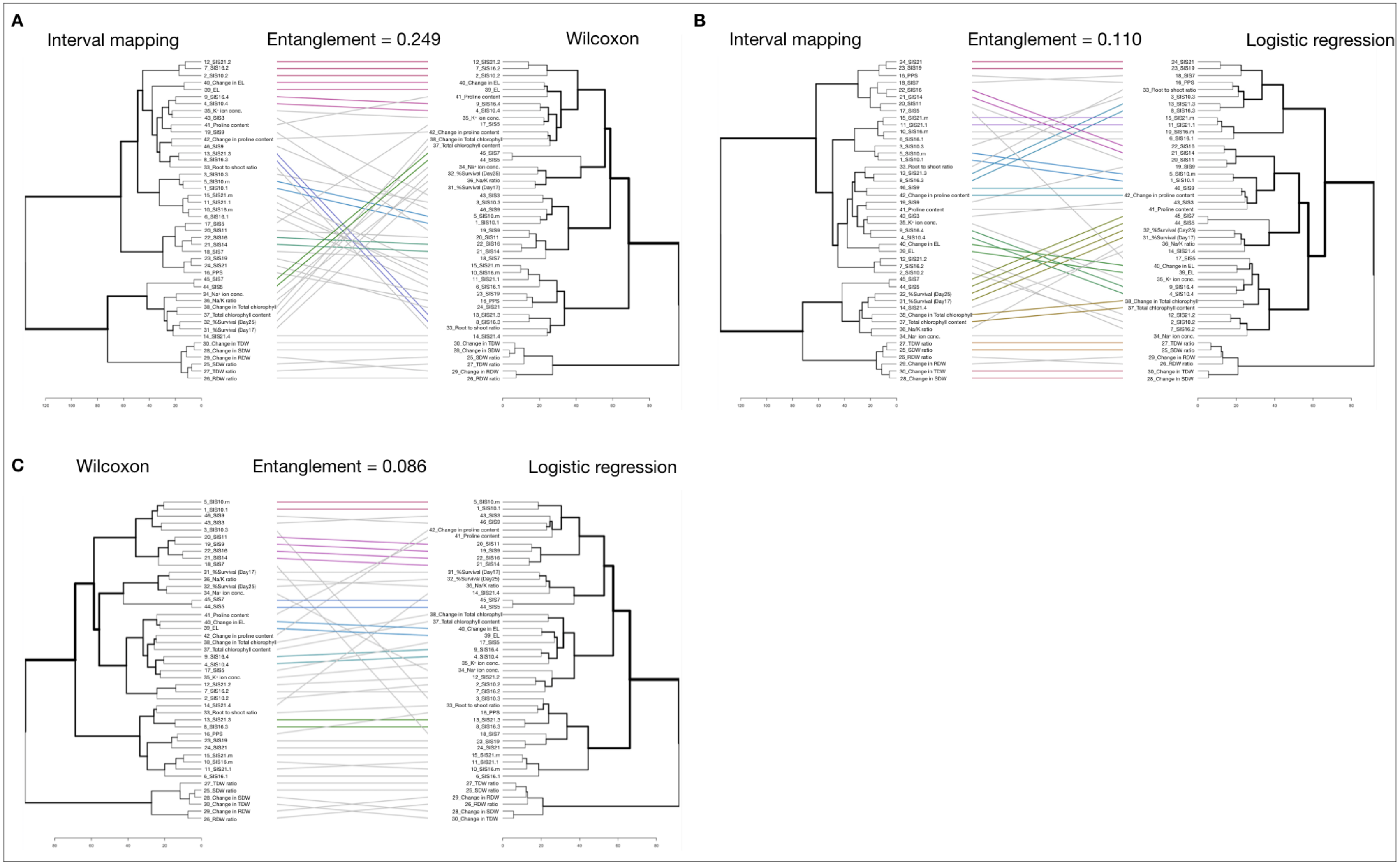

Figure S16. “Tanglegrams” between two dendrograms for visualising two hierarchical clusterings of PR-normalised confident values obtained from: (A) Interval mapping and Wilcoxon signed-rank test, (B) Interval mapping and Logistic regression analysis and (C) Wilcoxon signed-rank test and Logistic regression analysis.

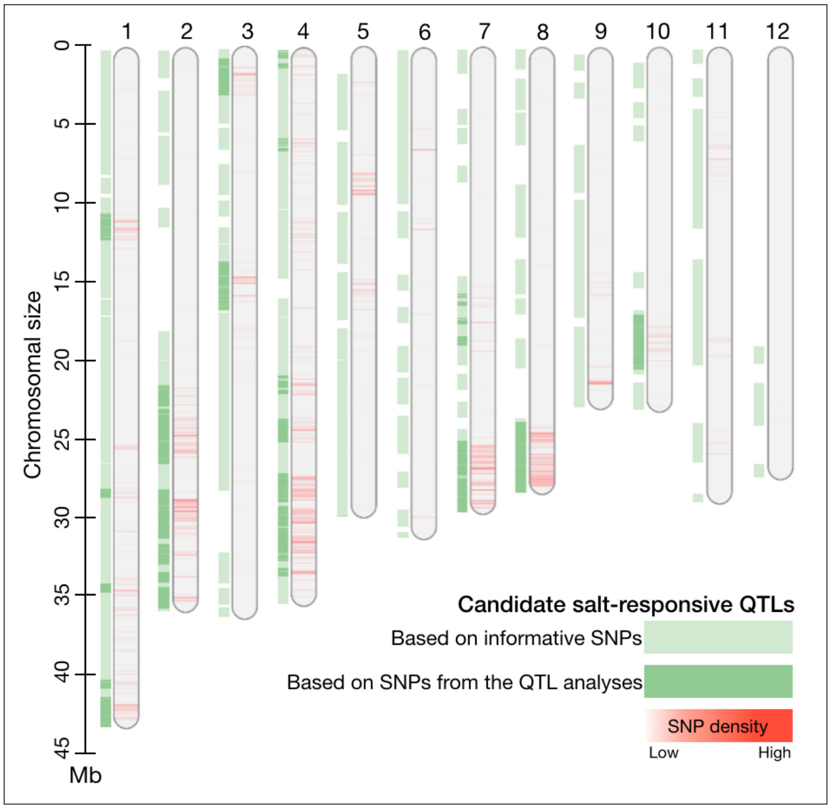

Figure S17. SNPs identified by the QTL analyses as the following criteria: n ≥ 5 CSSLs selection and the 80-th Percentile rank results in at least two methods. The QTL regions (dark green, 70.4 Mbp) based on the pipeline, covered 21.78% of candidate regions identified based on informative SNPs (light green, 323.2 Mbp) derived from the CSSL populations.

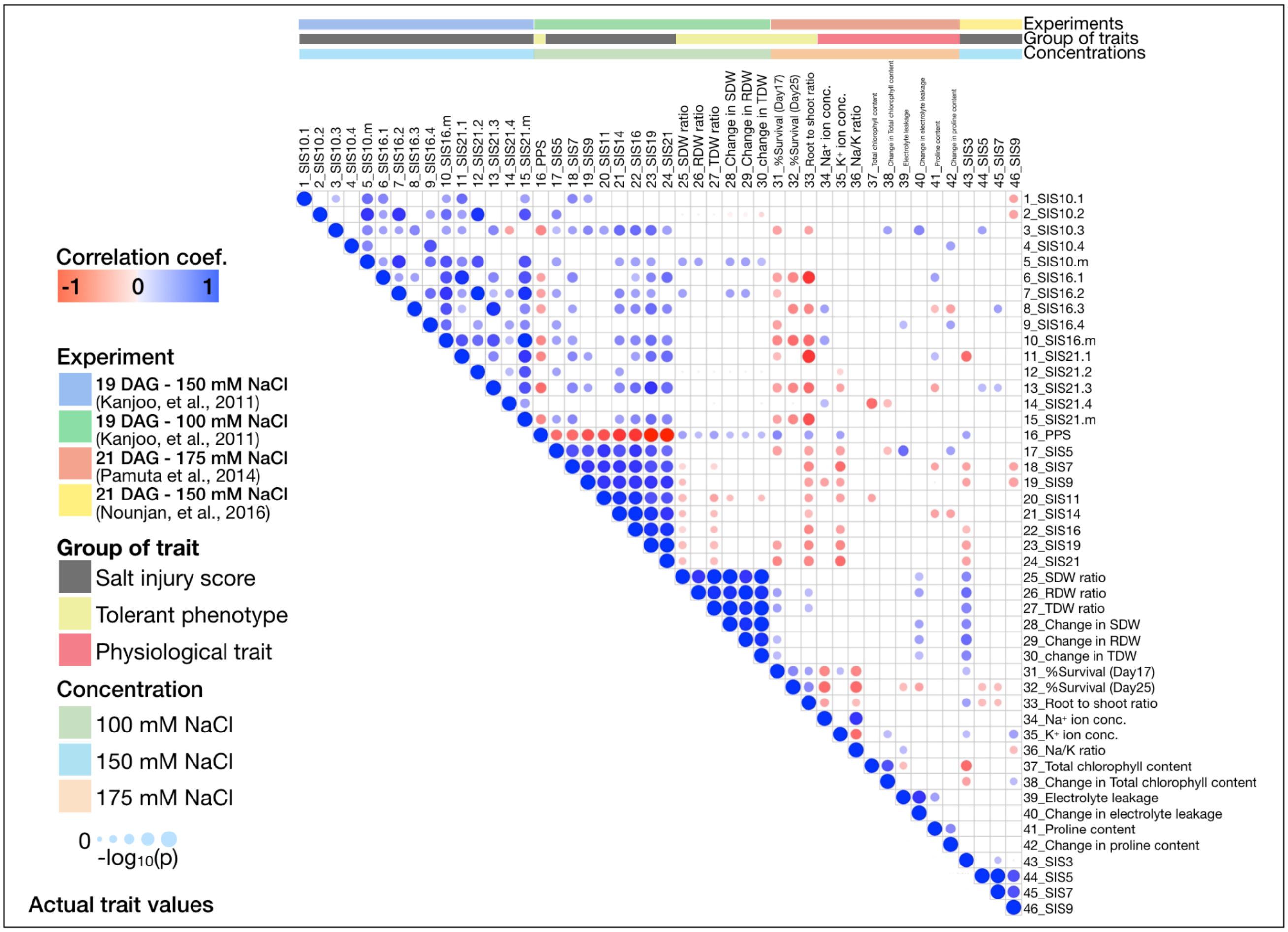

Figure S18. Spearman correlation matrix of phenotype scores of the 46 salinity-related traits of interest. The blue and red colours represent the correlation coefficient values. Dot sizes represent the p-values.

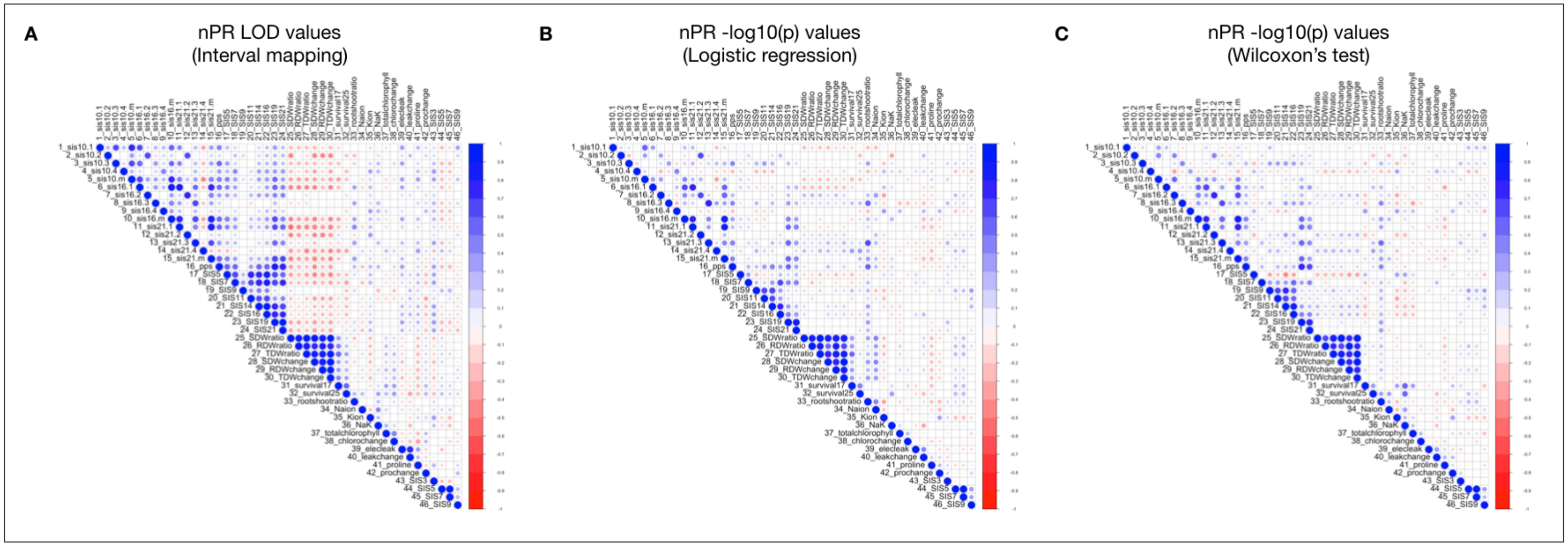

Figure S19. Spearman correlation matrix of the PR-normalised confidence scores obtained from: (A) Interval mapping, (B) Wilcoxon signed-rank test and (C) Logistic regression analysis of the 46 salt-responsive traits in this study. The blue and red colours represent the correlation coefficient values. Dot sizes represent the p-values.

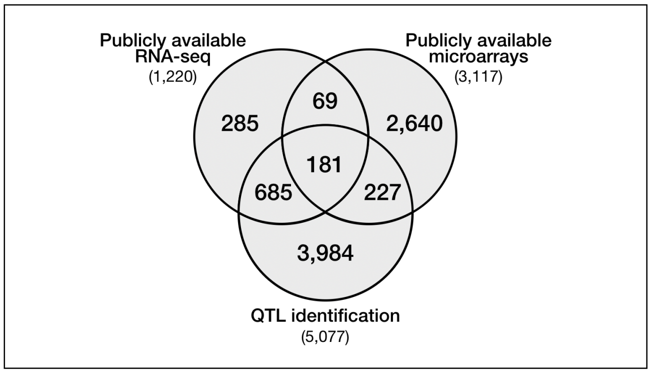

Figure S20. Numbers of salinity-related genes identified from our QTL identification pipeline, or from the publicly available microarray and RNA-seq datasets used in this study.

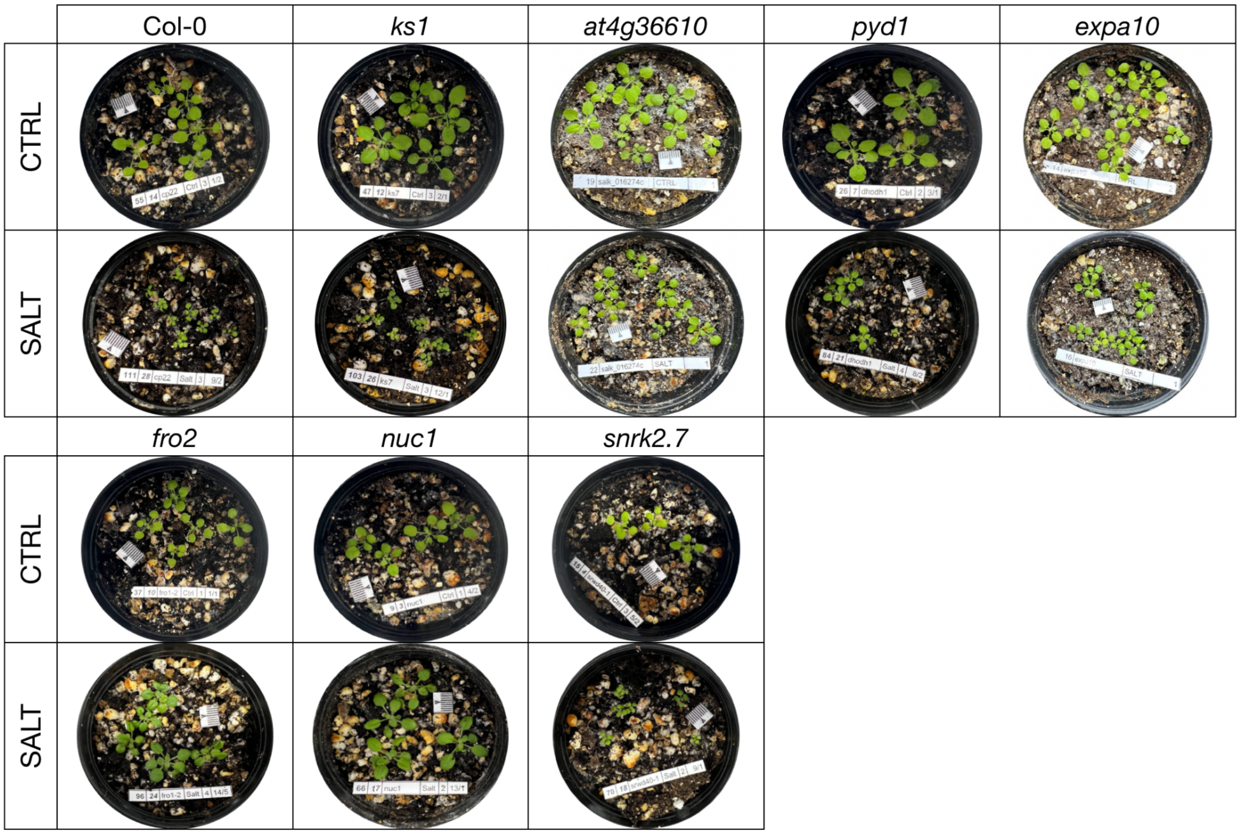

Figure S21. Morphological responses of wild-type (Col-0) and mutant Arabidopsis seedlings after 12 days grown under the control and salt-stress (250 mM NaCl) conditions.

frontiers

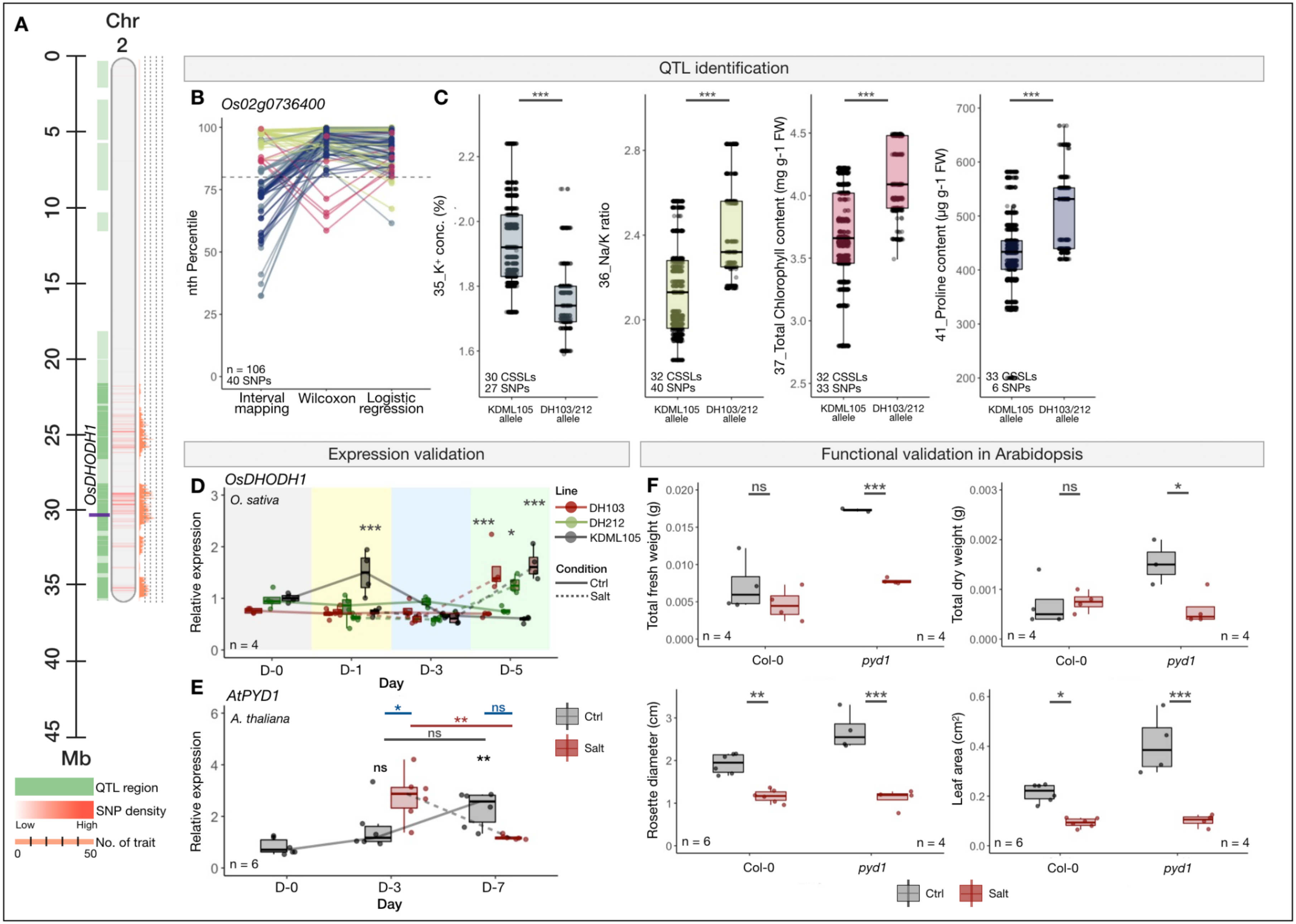

A
Chr 2
Mb
OsDHODH1
QTL region
SNP density
Low
High
No. of trait
QTL identification
B
Os02g0736400
nth Percentile
n = 106
40 SNPs
Interval mapping
Wilcoxon
Logistic regression
C
35_K+ conc. (%)
30 CSSLs
27 SNPs
36_Na/K ratio
32 CSSLs
40 SNPs
37_Total Chlorophyll content (mg g-1 FW)
32 CSSLs
33 SNPs
41_Proline content (µg g-1 FW)
33 CSSLs
6 SNPs
KDML105 allele
DH103/212 allele
Expression validation
Functional validation in Arabidopsis
D
OsDHODH1
O. sativa
Relative expression
n = 4
Day
Line
DH103
DH212
KDML105
Condition
Ctrl
Salt
E
AtPYD1
A. thaliana
Relative expression
n = 6
Day
Ctrl
Salt
F
Total fresh weight (g)
Total dry weight (g)
Rosette diameter (cm)
Leaf area (cm²)
Col-0
pyd1
n = 4
n = 6
Ctrl
Salt

Figure S22. Experimental validations of *OsDHODH1* in rice (*O. sativa*) and its orthologue in Arabidopsis (*A. thaliana*). (A) Genomic location of *OsDHODH1* in the QTL region on Chromosome 2 of *O. sativa* (Nipponbare cv.). (B) PR-normalised confidence scores of each SNP position within the *OsDHODH1* gene obtained from the three QTL identification methods. Colours representing the four traits are as described in C. (C) Phenotype scores of CSSLs with the alleles from salt-susceptible KDML105, or salt-tolerant DH103/DH212 cultivars in four salt-responsive traits (trait no. 35, 36, 37 and 41). Phenotype scores were obtained from Pamuta et al. (2014). (D) Expression analysis of *OsDHODH1* in KDML105, DH103 and DH212 rice cultivars under the control and salt stress (100 mM NaCl) condition. The salt treatment was conducted using 16-day-old rice seedlings. (E) Expression analysis of *OsDHODH1* orthologous gene in Arabidopsis, *AtPYD1*, under the control and salt stress (100 mM NaCl) condition. The salt treatment was conducted using 10-day-old Col-0 Arabidopsis seedlings. (F) Morphological responses, namely total fresh weight, total dry weight, rosette diameter and leaf area of WT (Col-0) and loss-of-function mutant line (*pyd1*). The experiment was conducted using 7-day-old seedlings and the measurement was done 12 days in the control or salt stress (250 mM NaCl) conditions. Error bars represent standard deviations from four biological replicates. Asterisks (*) represent the significant p-value (one-way ANOVA in D and E and t-test in F) between the control and salt stress conditions.

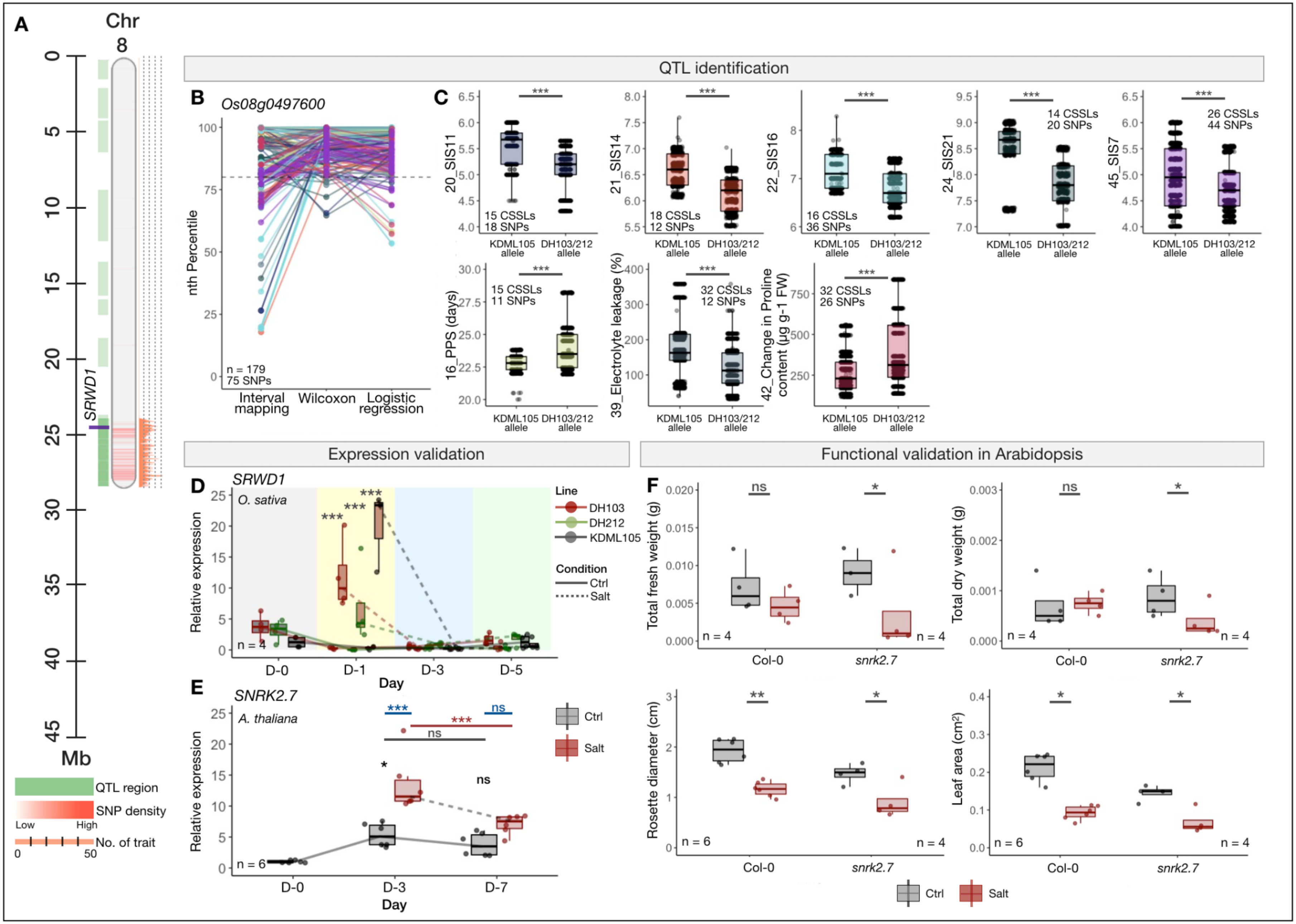

A
Chr 8
SRWD1
Mb
QTL region
SNP density
Low
High
No. of trait
QTL identification
B
Os08g0497600
nth Percentile
n = 179
75 SNPs
Interval mapping
Wilcoxon
Logistic regression
C
20_SIS11
15 CSSLs
18 SNPs
21_SIS14
18 CSSLs
12 SNPs
22_SIS16
16 CSSLs
36 SNPs
24_SIS21
14 CSSLs
20 SNPs
45_SIS7
26 CSSLs
44 SNPs
16_PPS (days)
15 CSSLs
11 SNPs
39_Electrolyte leakage (%)
32 CSSLs
12 SNPs
42_Change in Proline content (µg g-1 FW)
32 CSSLs
26 SNPs
KDML105 allele
DH103/212 allele
Expression validation
D
SRWD1
O. sativa
Relative expression
n = 4
Day
Line
DH103
DH212
KDML105
Condition
Ctrl
Salt
E
SNRK2.7
A. thaliana
n = 6
Ctrl
Salt
Functional validation in Arabidopsis
F
Total fresh weight (g)
Total dry weight (g)
Rosette diameter (cm)
Leaf area (cm²)
Col-0
snrk2.7
n = 4
n = 6

Figure S23. Experimental validations of *OsSRWD1* in rice (*O. sativa*) and its orthologue in Arabidopsis (*A. thaliana*). (A) Genomic location of *OsSRWD1* in the QTL region on Chromosome 8 of *O. sativa* (Nipponbare cv.). (B) PR-normalised confidence scores of each SNP position within the *OsSRWD1* gene obtained from the three QTL identification methods. Colours representing the four traits are as described in C. (C) Phenotype scores of CSSLs with the alleles from salt-susceptible KDML105, or salt-tolerant DH103/DH212 cultivars in 8 salt-responsive traits (trait no. 6, 20, 21, 22, 24, 39, 42 and 45). Phenotype scores were obtained from Kanjoo et al. (2011), Pamuta et al. (2014) and Nuanjan et al. (2016). (D) Expression analysis of *OsSRWD1* in KDML105, DH103 and DH212 rice cultivars under the control and salt stress (100 mM NaCl) condition. The salt treatment was conducted using 16-day-old rice seedlings. (E) Expression analysis of *OsSRWD1* orthologous gene in Arabidopsis, *AtSNRK2.7*, under the control and salt stress (100 mM NaCl) condition. The salt treatment was conducted using 10-day-old Col-0 Arabidopsis seedlings. (F) Morphological responses, namely total fresh weight, total dry weight, rosette diameter and leaf area of WT (Col-0) and loss-of-function mutant line (*snrk2.7*). The experiment was conducted using 7-day-old seedlings and the measurement was done 12 days in the control or salt stress (250 mM NaCl) conditions. Error bars represent standard deviations from four biological replicates. Asterisks (*) represent the significant p-value (one-way ANOVA in D and E and t-test in F) between the control and salt stress conditions.

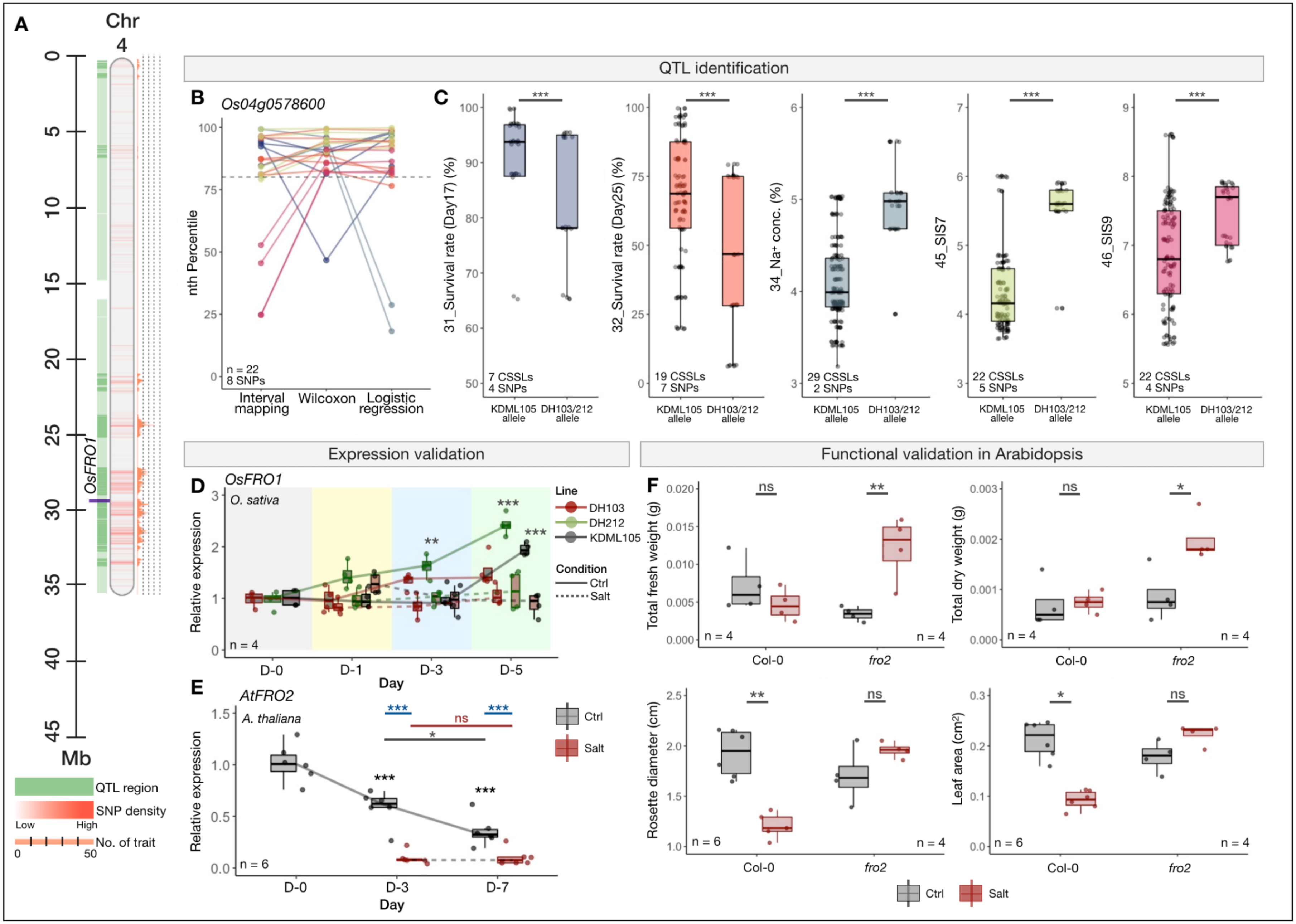

A
Chr 4
OsFRO1
Mb
QTL region
SNP density
Low
High
No. of trait
QTL identification
B
Os04g0578600
nth Percentile
n = 22
8 SNPs
Interval mapping
Wilcoxon
Logistic regression
C
31_Survival rate (Day17) (%)
7 CSSLs
4 SNPs
32_Survival rate (Day25) (%)
19 CSSLs
7 SNPs
34_Na+ conc. (%)
29 CSSLs
2 SNPs
45_SIS7
22 CSSLs
5 SNPs
46_SIS9
22 CSSLs
4 SNPs
KDML105 allele
DH103/212 allele
Expression validation
D
OsFRO1
O. sativa
Relative expression
n = 4
D-0
D-1
D-3
D-5
Day
Line
DH103
DH212
KDML105
Condition
Ctrl
Salt
E
AtFRO2
A. thaliana
Relative expression
n = 6
D-0
D-3
D-7
Day
Ctrl
Salt
Functional validation in Arabidopsis
F
Total fresh weight (g)
Total dry weight (g)
Rosette diameter (cm)
Leaf area (cm²)
Col-0
fro2
n = 4
n = 6
Ctrl
Salt

Figure S24. Experimental validations of *OsFRO1* in rice (*O. sativa*) and its orthologue in Arabidopsis (*A. thaliana*). (A) Genomic location of *OsFRO1* in the QTL region on Chromosome 4 of *O. sativa* (Nipponbare cv.). (B) PR-normalised confidence scores of each SNP position within the *OsFRO1* gene obtained from the three QTL identification methods. Colours representing the four traits are as described in C. (C) Phenotype scores of CSSLs with the alleles from salt-susceptible KDML105, or salt-tolerant DH103/DH212 cultivars in five salt-responsive traits (trait no. 31, 32, 34, 45 and 46). Phenotype scores were obtained from Pamuta et al. (2014) and Nuanjan et al. (2016). (D) Expression analysis of *OsFRO1* in KDML105, DH103 and DH212 rice cultivars under the control and salt stress (100 mM NaCl) condition. The salt treatment was conducted using 16-day-old rice seedlings. (E) Expression analysis of *OsFRO1* orthologous gene in Arabidopsis, *AtFRO2*, under the control and salt stress (100 mM NaCl) condition. The salt treatment was conducted using 10-day-old Col-0 Arabidopsis seedlings. (F) Morphological responses, namely total fresh weight, total dry weight, rosette diameter and leaf area of WT (Col-0) and loss-of-function mutant line (*fro2*). The experiment was conducted using 7-day-old seedlings and the measurement was done 12 days in the control or salt stress (250 mM NaCl) conditions. Error bars represent standard deviations from four biological replicates. Asterisks (*) represent the significant p-value (one-way ANOVA in D and E and t-test in F) between the control and salt stress conditions.

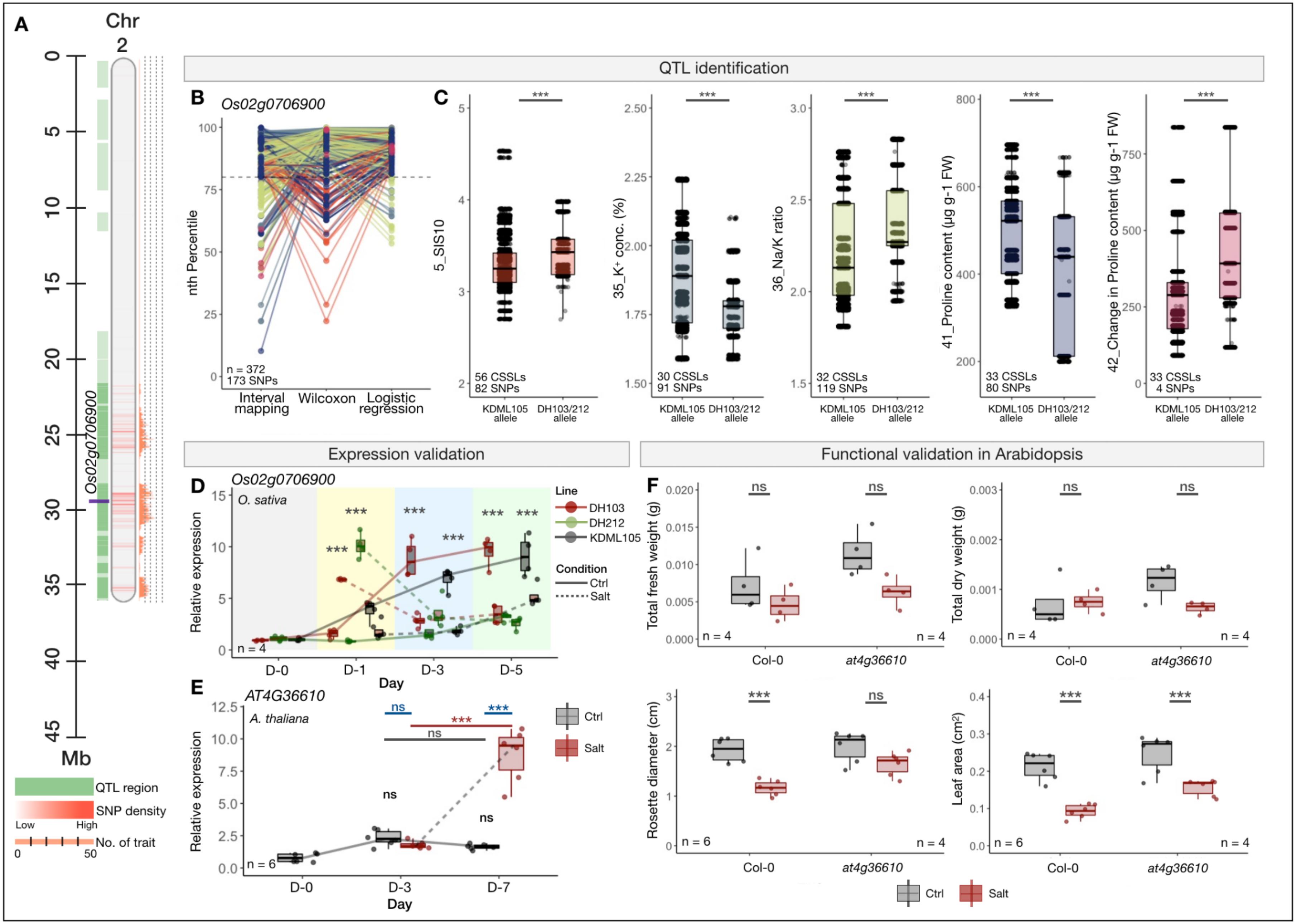
A
Chr 2
Mb
Os02g0706900
QTL region
SNP density
Low
High
No. of trait
QTL identification
B
Os02g0706900
nth Percentile
n = 372
173 SNPs
Interval mapping
Wilcoxon
Logistic regression
C
5_SIS10
56 CSSLs
82 SNPs
35_K⁺ conc. (%)
30 CSSLs
91 SNPs
36_Na/K ratio
32 CSSLs
119 SNPs
41_Proline content (μg g-1 FW)
33 CSSLs
80 SNPs
42_Change in Proline content (μg g-1 FW)
33 CSSLs
4 SNPs
KDML105 allele
DH103/212 allele
Expression validation
D
Os02g0706900
O. sativa
Relative expression
Day
Line
DH103
DH212
KDML105
Condition
Ctrl
Salt
n = 4
E
AT4G36610
A. thaliana
Relative expression
Day
Ctrl
Salt
n = 6
Functional validation in Arabidopsis
F
Total fresh weight (g)
Total dry weight (g)
Rosette diameter (cm)
Leaf area (cm²)
Col-0
at4g36610
Ctrl
Salt

Figure S25. Experimental validations of *Os02g0706900* in rice (*O. sativa*) and its orthologue in Arabidopsis (*A. thaliana*). (A) Genomic location of *Os02g0706900* in the QTL region on Chromosome 2 of *O. sativa* (Nipponbare cv.). (B) PR-normalised confidence scores of each SNP position within the *Os02g0706900* gene obtained from the three QTL identification methods. Colours representing the five traits are as described in C. (C) Phenotype scores of CSSLs with the alleles from salt-susceptible KDML105, or salt-tolerant DH103/DH212 cultivars in four salt-responsive traits (trait no. 5, 35, 36, 41 and 42). Phenotype scores were obtained from Kanjoo et al. (2011) and Pamuta et al. (2014). (D) Expression analysis of *Os02g0706900* in KDML105, DH103 and DH212 rice cultivars under the control and salt stress (100 mM NaCl) condition. The salt treatment was conducted using 16-day-old rice seedlings. (E) Expression analysis of *Os02g0706900* orthologous gene in Arabidopsis, *AT4G36610*, under the control and salt stress (100 mM NaCl) condition. The salt treatment was conducted using 10-day-old Col-0 Arabidopsis seedlings. (F) Morphological responses, namely total fresh weight, total dry weight, rosette diameter and leaf area of WT (Col-0) and loss-of-function mutant line (*At4g36610*). The experiment was conducted using 7-day-old seedlings and the measurement was done 12 days in the control or salt stress (250 mM NaCl) conditions. Error bars represent standard deviations from four biological replicates. Asterisks (*) represent the significant p-value (one-way ANOVA in D and E and t-test in F) between the control and salt stress conditions.

frontiers

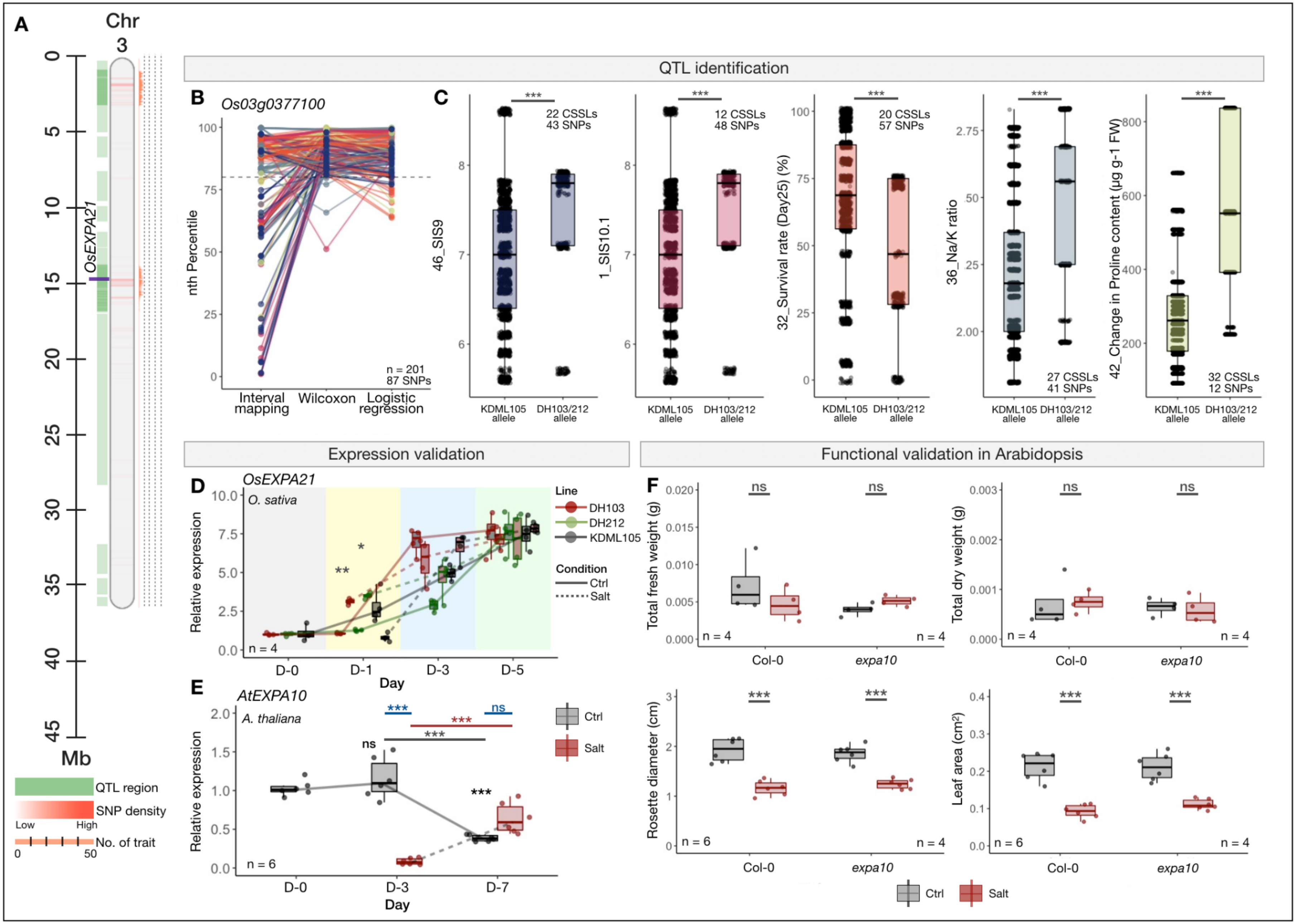

A
Chr 3
OsEXPA21
Mb
QTL region
SNP density
Low High
No. of trait
QTL identification
B
Os03g0377100
nth Percentile
n = 201
87 SNPs
Interval mapping
Wilcoxon
Logistic regression
C
46_SIS9
22 CSSLs
43 SNPs
1_SIS10.1
12 CSSLs
48 SNPs
32_Survival rate (Day25) (%)
20 CSSLs
57 SNPs
36_Na/K ratio
27 CSSLs
41 SNPs
42_Change in Proline content (µg g-1 FW)
32 CSSLs
12 SNPs
KDML105 allele
DH103/212 allele
Expression validation
D
OsEXPA21
O. sativa
Relative expression
Line
DH103
DH212
KDML105
Condition
Ctrl
Salt
n = 4
D-0
D-1
D-3
D-5
Day
E
AtEXPA10
A. thaliana
Relative expression
Ctrl
Salt
n = 6
D-0
D-3
D-7
Day
Functional validation in Arabidopsis
F
Total fresh weight (g)
Total dry weight (g)
Rosette diameter (cm)
Leaf area (cm²)
Col-0
expa10
n = 4
n = 6
Ctrl
Salt

Figure S26. Experimental validations of *OsEXPA21* in rice (*O. sativa*) and its orthologue in Arabidopsis (*A. thaliana*). (A) Genomic location of *OsEXPA21* in the QTL region on Chromosome 2 of *O. sativa* (Nipponbare cv.). (B) PR-normalised confidence scores of each SNP position within the *OsEXPA21* gene obtained from the three QTL identification methods. Colours representing the four traits are as described in C. (C) Phenotype scores of CSSLs with the alleles from salt-susceptible KDML105, or salt-tolerant DH103/DH212 cultivars in five salt-responsive traits (trait no. 1, 32, 36, 42 and 46). Phenotype scores were obtained from Kanjoo et al. (2011), Pamuta et al. (2014) and Nuanjan et al. (2016). (D) Expression analysis of *OsEXPA21* in KDML105, DH103 and DH212 rice cultivars under the control and salt stress (100 mM NaCl) condition. The salt treatment was conducted using 16-day-old rice seedlings. (E) Expression analysis of *OsEXPA21* orthologous gene in Arabidopsis, *AtEXPA10*, under the control and salt stress (100 mM NaCl) condition. The salt treatment was conducted using 10-day-old Col-0 Arabidopsis seedlings. (F) Morphological responses, namely total fresh weight, total dry weight, rosette diameter and leaf area of WT (Col-0) and loss-of-function mutant line (*expa10*). The experiment was conducted using 7-day-old seedlings and the measurement was done 12 days in the control or salt stress (250 mM NaCl) conditions. Error bars represent standard deviations from four biological replicates. Asterisks (*) represent the significant p-value (one-way ANOVA in D and E and t-test in F) between the control and salt stress conditions.
